# Impact of renin–angiotensin–aldosterone system inhibition on mortality in critically ill COVID-19 patients with pre-existing hypertension: a prospective cohort study

**DOI:** 10.1186/s12872-022-02565-1

**Published:** 2022-03-23

**Authors:** Kei Sato, Nicole White, Jonathon P. Fanning, Nchafatso Obonyo, Michael H. Yamashita, Vinesh Appadurai, Anna Ciullo, Meryta May, Elliott T. Worku, Leticia Helms, Shinichiro Ohshimo, Dafsah A. Juzar, Jacky Y. Suen, Gianluigi Li Bassi, John F. Fraser, Rakesh C. Arora, COVID-19 Critical Care Consortium Investigators, Gianluigi Li Bassi, Jacky Y. Suen, Heidi J. Dalton, John Laffey, Daniel Brodie, Eddy Fan, Antoni Torres, Davide Chiumello, Alyaa Elhazmi, Carol Hodgson, Shingo Ichiba, Carlos Luna, Srinivas Murthy, Alistair Nichol, Pauline Yeung Ng, Mark Ogino, Eva Marwali, Ian Yang, Grad Dip, Giacomo Grasselli, Robert Bartlett, Aidan Burrell, John F. Fraser

**Affiliations:** 1 Critical Care Research Group, The Prince Charles Hospital, Level 3, Clinical Sciences Building, Chermside, Brisbane, QLD 4032 Australia; 2 Faculty of Medicine, University of Queensland, Brisbane, QLD Australia; 3 Australian Centre for Health Services Innovation (AusHSI) and Centre for Healthcare Transformation, Queensland University of Technology (QUT), Brisbane, QLD Australia; 4 Nuffield Department of Population Health, University of Oxford, Oxford, UK; 5 Wellcome Trust Centre for Global Health Research, Imperial College London, London, UK; 6 Initiative to Develop African Research Leaders/KEMRI-Wellcome Trust Research Programme, Kilifi, Kenya; 7 Section of Cardiac Surgery, Department of Surgery, Max Rady College of Medicine, University of Manitoba, Winnipeg, MB Canada; 8 Department of Cardiology, The Prince Charles Hospital, Brisbane, QLD Australia; 9 Division of Emergency Medicine, Department of Surgery, University of Utah Health, Salt Lake City, UT 84132 USA; 10 Department of Microbiology, Sullivan Nicolaides Pathology, Brisbane, QLD Australia; 11 Adult Intensive Care Services, The Prince Charles Hospital, Brisbane, QLD Australia; 12 Department of Emergency and Critical Care Medicine, Graduate School of Biomedical and Health Sciences, Hiroshima University, Hiroshima, Japan; 13 Intensive Cardiovascular Care Unit, National Cardiovascular Center Harapan Kita, Jakarta, Indonesia; 14 Division Intensive & Emergency Cardiovascular Care, Department Cardiology and Vascular Medicine, Faculty of Medicine, University of Indonesia, Jakarta, Indonesia; 15 Institut d’Investigacions Biomediques August Pi i Sunyer, Barcelona, Spain

**Keywords:** Angiotensin-converting enzyme inhibitors, Angiotensin receptor blockers, Severe acute respiratory syndrome coronavirus 2, COVID-19, Critical care

## Abstract

**Background:**

The influence of renin–angiotensin–aldosterone system (RAAS) inhibitors on the critically ill COVID-19 patients with pre-existing hypertension remains uncertain. This study examined the impact of previous use of angiotensin-converting enzyme inhibitors (ACEi) and angiotensin receptor blockers (ARB) on the critically ill COVID-19 patients.

**Methods:**

Data from an international, prospective, observational cohort study involving 354 hospitals spanning 54 countries were included. A cohort of 737 COVID-19 patients with pre-existing hypertension admitted to intensive care units (ICUs) in 2020 were targeted. Multi-state survival analysis was performed to evaluate in-hospital mortality and hospital length of stay up to 90 days following ICU admission.

**Results:**

A total of 737 patients were included—538 (73%) with pre-existing hypertension had received ACEi/ARBs before ICU admission, while 199 (27%) had not. Cox proportional hazards model showed that previous ACEi/ARB use was associated with a decreased hazard of in-hospital death (HR, 0.74, 95% CI 0.58–0.94). Sensitivity analysis adjusted for propensity scores showed similar results for hazards of death. The average length of hospital stay was longer in ACEi/ARB group with 21.2 days (95% CI 19.7–22.8 days) in ICU and 6.7 days (5.9–7.6 days) in general ward compared to non-ACEi/ARB group with 16.2 days (14.1–18.6 days) and 6.4 days (5.1–7.9 days), respectively. When analysed separately, results for ACEi or ARB patient groups were similar for both death and discharge.

**Conclusions:**

In critically ill COVID-19 patients with comorbid hypertension, use of ACEi/ARBs prior to ICU admission was associated with a reduced risk of in-hospital mortality following adjustment for baseline characteristics although patients with ACEi/ARB showed longer length of hospital stay.

*Clinical trial registration* The registration number: ACTRN12620000421932; The date of registration: 30, March 2020; The URL of the registration: https://www.australianclinicaltrials.gov.au/anzctr/trial/ACTRN12620000421932.

**Supplementary Information:**

The online version contains supplementary material available at 10.1186/s12872-022-02565-1.

## Background

The effect of renin–angiotensin–aldosterone system (RAAS) therapy on an individual’s susceptibility to, and severity of, COVID–19 has been a source of debate throughout the COVID-19 pandemic [1–3]. The biological rationale for this arises from the understanding that severe acute respiratory syndrome coronavirus-2 (SARS-CoV-2), the viral agent responsible for COVID-19, enters human target cells by binding to the membrane-bound mono-carboxypeptidase—angiotensin-converting enzyme 2 (ACE-2)—resulting in both internalization and degradation of the enzyme [4–6]. ACE-2 expression is especially high in respiratory epithelium [7]—the main route of SARS-CoV-2 entry into the body.

Mechanistically, treatment with RAAS inhibitors—like angiotensin-converting enzyme inhibitors (ACEi) and angiotensin receptor blockers (ARBs)—is known to induce the upregulation of ACE-2 expression, and it is around this that speculation hinges and has resulted in conflicting hypotheses [1–3, 8, 9]. On one hand, RAAS inhibitors could promote more severe COVID-19, with upregulated ACE-2 increasing the substrate for SARS-CoV-2 infectivity and severity [10, 11]. Conversely, ACE-2 upregulation may protect the lung via its downstream breakdown of angiotensin II and by increasing the expression of angiotensin-1–7 and 1–9, both of which have vasodilatory and anti-inflammatory effects. This controversy has resulted in the release of statements, from health regulatory authorities and scientific societies, recommending that patients should not discontinue ACEi/ARB therapy in the absence of conclusive evidence of harm [12].

The aim of this study was to examine the role of ACEi/ARB exposure on outcomes among COVID-19 patients with pre-existing hypertension admitted to intensive care units (ICUs). Outcomes included in-hospital mortality (primary outcome), length of ICU stay and general ward stay. We used prospectively-collected data from the international COVID-19 Critical Care Consortium incorporating ExtraCorporeal Membrane Oxygenation for 2019 novel Coronavirus Acute Respiratory Disease (COVID-19–CCC/ECMOCARD) [13].

## Methods

### Study design and subject participation

Study data were extracted for analysis from the COVID-19-CCC/ECMOCARD registry, the rationale and design of which have been detailed in Additional file 1: Document S1 and previous publication [13]. COVID-19-CCC/ECMOCARD is an international observational cohort study involving 354 hospitals spanning 54 countries across six continents. All participating sites obtained local ethics committee approval, and waivers of informed consent were granted for all patients. Recruiting sites and all contributors/collaborators are listed in Additional file 1: Document S2. The COVID-19-CCC collaborates through the International Severe Acute Respiratory and Emerging Infection Consortium (ISARIC) and their Short PeRiod IncideNce sTudy of Severe Acute Respiratory Infection (SPRINT-SARI). De-identified data were collected prospectively (but not necessarily consecutively) for enrolled patients and stored via the REDCap (Vanderbilt/NIH/NCATS UL1 TRooo445 v.10.0.23) electronic data capture tool hosted at the University of Oxford in the United Kingdom and the University of Queensland in Australia.

Inclusion criteria were: (1) age ≥ 18 years, (2) clinically suspected or laboratory confirmed SARS-CoV-2 infection, (3) admission to an ICU at any time during hospitalisation, (4) hypertension recorded as a pre-existing comorbidity at the time of hospital admission, and (5) knowledge of whether they had previously received (taken within 14 days of hospital admission) any antihypertensive therapy. Patients who met all the criteria from (1) to (5) were enrolled. Patients with clinically suspected COVID-19 who returned a negative result for SARS-CoV-2 infection by Polymerase Chain Reaction or next generation sequencing were excluded. Hypertension was defined based on the standardised definition specified in the COVID-19 ISARIC case report form as someone having elevated arterial blood pressure diagnosed clinically, > 140 mmHg systolic or > 90 mmHg diastolic (yes, no, unknown). Patients with pre-existing hypertension not on antihypertensive therapy were excluded.

Patients with pre-existing hypertension (regardless of the blood pressure on admission or during hospital stay) then were divided into two groups based on reported use of antihypertensive therapy within two weeks of hospital admission, as collected by the COVID-19 ISARIC case report form. Patients receiving ACEi and/or ARB therapy were defined as the ACEi/ARB group. Patients on other antihypertensive therapies other than ACEi and/or ARB were defined as the non-ACEi/ARB.

### Data collection and outcome measures

For all enrolled patients, the following information was collected using an electronic case report form in Additional file 1: Document S3: demographics, comorbidities, medications, laboratory values, complications, and outcomes. Additional case report forms in Additional file 1: Document S4 were completed for patients who required mechanical ventilation or extracorporeal membrane oxygenation (ECMO). Analyses were performed on all eligible patients included in the database from December 1st, 2019 through December 30th, 2020. Outcomes included in-hospital mortality (primary outcome), length of ICU stay and length of general ward stay assessed up to 90 days following ICU admission.

### Statistical analysis

Baseline characteristics were summarized by descriptive statistics stratified by patient group. Characteristics covered patient demographics, comorbidities, admission signs and symptoms and laboratory results within the first day of ICU admission. Complications during hospitalization, the use of different management strategies in the first 28 days of ICU admission, and final outcomes at the end of the study were also summarized. Continuous variables were reported as medians with interquartile ranges (IQR). Categorial variables were reported as frequencies with percentages. The number of available observations were reported for all variables to show levels of data completeness. Hypothesis testing of between group differences in baseline characteristics was deemed inappropriate following recommendations for statistical reporting of observational studies [14].

Length of stay and in-hospital mortality were analysed as time-to-event outcomes using multi-state survival analysis. Modelling as time-to-event outcomes allowed us to include data on all patients regardless of outcome and accounted for death and discharged alive as competing risks. Outcomes were modelled up to 90 days following hospital admission. Independent right censoring was applied to patients who were still in hospital at 90 days, at their last known follow-up time or at date of transfer to another facility.

Expected length of stay was examined separately for each patient group using a multistate model, unadjusted for baseline characteristics. The model was defined by four states: ICU, General ward/Hospitalised, Discharged alive, Died (Additional file 2: Fig. S1). Patients entered the model through the general ward state, if not admitted to ICU on day 0 of hospitalisation, or the ICU state if admitted to ICU on the same day as hospital admission. Whilst in ICU, patients either died or returned to the general ward after being discharged from ICU. Following ICU discharge, patients either died or were discharged alive from hospital. Length of stay was estimated from expected times spent in the general ward and ICU states. Unadjusted estimates of cumulative morality risk at 30, 60 and 90 days from ICU admission were estimated from cumulative incidence functions starting in the ICU state, accounting for hospital discharge as a competing risk.

Follow-up analysis examined the influence of ACEi/ARB use on the hazards of death and discharged alive, accounting for baseline characteristics. Outcomes were analysed using a multi-state Cox proportional hazard model. Baseline characteristics included as model covariates were patient group, age, sex, body mass index (BMI), week of ICU admission, geographic region and major ethnicities (Black, Latin American, South Asian, White/Caucasian, Other including minority groups), selected comorbidities (diabetes, smoking, chronic cardiac disease, chronic kidney disease) and corticosteroid use during hospitalisation (yes, no). Chosen covariates were based on the previous research on COVID-19 outcomes in hypertensive patients [9]. Missing data in covariates (BMI 7%, Chronic cardiac disease < 1%, Chronic kidney diseases < 1%, Diabetes < 1%, Smoking 23%, Corticosteroids 7%) was assumed missing at random and imputed by multiple imputation using chained equations (MICE). Tests for proportionality based on Schoenfeld residuals were applied to all covariates [14]; covariates not satisfying the proportional hazards assumption were instead used to stratify the baseline hazard function. Model results were reported separately for death and discharged alive as pooled hazard ratios with 95% confidence intervals (CI).

We further considered adjusting for the influence of baseline characteristics on reported use of ACEi/ARB versus non-ACEi/ARB treatment(s) before admission. Analysis followed recommendations for inverse probability weighting applied for time-to-event outcomes [15]. Inverse probability weights were defined using propensity scores that estimated the probability of belonging to the ACEi/ARB group. Propensity scores considered the same baseline characteristics applied in the Cox proportional hazards model. Resulting propensity scores were then used to weight observations in a multi-state Cox model with patient group as the only covariate.

To evaluate differential effects between ACEi and ARB use, sensitivity analysis considered patient stratification into ACEi, ARB and non-ACEi/ARB groups; associations with the hazards of death and discharge were explored.

All analyses were completed in R 4.0.3. Code for multistate analysis of length of stay was adapted from a published study on COVID-19 patients [16].

## Results

### Patient characteristics

During the period of study, a total of 1193 patients with COVID-19 and pre-existing hypertension were admitted to COVID-19-CCC participating ICUs. Of these, 456 patients with missing data of antihypertensive therapy were excluded according to the inclusion criteria. The final cohort for statistical analysis comprised of 737 participants with pre-existing hypertension on antihypertensive therapy, from 98 study hospitals (Additional file 2: Fig. S2). The median age of patients was 65 years [IQR, 57–73] and 481 were male (65%). The median Sepsis-related Organ Failure Assessment (SOFA) score and Acute Physiology and Chronic Health Evaluation II (APACHE-II) score were 6 [IQR, 4–9] and 17 [IQR, 12–24], respectively.

### Baseline characteristics

A total of 538 patients (73%) reported use of ACEi/ARB therapy (median age 65 years [IQR, 57–73], 67% men) within the two weeks prior to ICU admission, while 199 (27%) did not (median age 66 years [IQR, 55–73], 62% men). Admission characteristics of the ACEi/ARB and non-ACEi/ARB groups are compared in Tables 1 and 2. Both groups included similar percentage of diabetes and chronic cardiac disease {ACEi/ARB group vs. non-ACEi/ARB group: Diabetes, 47% vs. 45%; chronic cardiac disease, 25% vs. 30%}. Chronic kidney disease was reported less in the ACEi/ARB group {15% vs. 26%}. The usage of calcium channel blocker (CCB) and β-blocker was less frequent in ACEi/ARB groups than non-ACEi/ARB groups {25% vs. 56%, and 24% vs. 55%, respectively}.

**Table 1:** Baseline characteristics

Characteristic	ACEi/ARB	Available number	Non-ACEi/ARB	Available number
*Demographics*				
Age (years), median (IQR)	65 (57–73)	538	66 (55–73)	199
Male, n (%)	358 (67)	538	123 (62)	199
BMI (kg/m^2^), median (IQR)	29.4 (26.2–34.0)	489	29.3 (24.9–34.0)	190
Ethnicity, n (%)				
Aboriginal	7 (1)	501	1 (1)	191
Arab	11 (2)	501	4 (2)	191
Black	58 (12)	501	48 (25)	191
East Asian	20 (4)	501	10 (5)	191
South Asian	21 (4)	501	13 (7)	191
West Asian	3 (1)	501	1 (1)	191
Latin American	102 (20)	501	17 (9)	191
Other	26 (5)	501	14 (7)	191
White	253 (50)	501	83 (43)	191
Geographic region, n (%)				
Africa	19 (4)	538	0 (0)	199
Asia	53 (10)	538	37 (19)	199
Australia	6 (1)	538	1 (1)	199
Europe	164 (30)	538	57 (29)	199
Latin America and the Caribbean	102 (19)	538	8 (4)	199
Northern America	194 (36)	538	96 (48)	199
*Admission signs and symptoms*				
Heart rate (beats/minute), median (IQR)	92 (80–105)	510	92 (79–106)	182
Systolic BP (mmHg), median (IQR)	130 (114–148)	508	127 (110–150)	184
Diastolic BP (mmHg), median (IQR)	72 (62–82)	508	70 (61–83)	184
Respiratory rate (breaths/minute), median (IQR)	25 (20–30)	485	24 (20–30)	177
Oxygen saturation (%), median (IQR)	91 (84–95)	511	94 (89–97)	187
Cough, n (%)	377 (75)	502	127 (70)	181
Fever, n (%)	408 (79)	514	140 (75)	186
Malaise, n (%)	275 (58)	478	77 (44)	175
Dyspnoea, n (%)	433 (82)	526	156 (82)	190
*Reported comorbidities*				
Smoking, n (%)	166 (40)	412	67 (44)	151
Diabetes, n (%)	250 (47)	534	88 (45)	197
Chronic cardiac disease, n (%)	133 (25)	534	59 (30)	198
Chronic pulmonary disease, n (%)	72 (13)	536	41 (21)	197
Chronic kidney disease, n (%)	80 (15)	535	51 (26)	197
Chronic neurological disorder, n (%)	34 (6)	534	17 (9)	197
Severe liver disease, n (%)	27 (5)	536	21 (11)	198
Malignant neoplasm, n (%)	32 (6)	535	13 (7)	197
*Reported use of anti-hypertensive drugs on admission*				
Diuretic, n (%)	92 (20)	458	46 (23)	199
Calcium channel blocker, n (%)	113 (25)	458	112 (56)	199
β-blocker, n (%)	111 (24)	458	110 (55)	199
α-blocker, n (%)	6 (1)	458	4 (2)	199

*ACEi* angiotensin-converting enzyme inhibitor, *ARB* angiotensin II receptor blocker, *BMI* body mass index

**Table 2:** Laboratory examinations within first 24 h of ICU admission

Characteristic	ACEi/ARB median (IQR)	Available number	Non-ACEi/ARB median (IQR)}	Available number
Haemoglobin (g/L)	12.7 (11.1–13.8)	414	11.4 (9.6–13.3)	165
Neutrophil (10^9^/L)	8.7 (5.7–11.9)	307	7.1 (4.2–11.0)	100
Lymphocyte (10^9^/L)	0.8 (0.5–1.2)	319	0.7 (0.4–1.1)	111
Platelets (10^9^/L)	220 (168–280)	395	190 (134–261)	162
C-reactive protein (mg/L)	133 (50–257)	125	118 (36–245)	59
Procalcitonin (ng/mL)	0.30 (0.17–0.94)	141	0.70 (0.25–1.67)	51
Bilirubin (μmol/L)	0.58 (0.35–0.90)	302	0.60 (0.40–1.00)	123
AST (U/L)	0.81 (0.57–1.25)	254	0.82 (0.57–1.20)	105
ALT (U/L)	0.61 (0.38–1.15)	257	0.52 (0.33–0.87)	109
Blood urea nitrogen (mmol/L)	2.1 (1.2–3.6)	359	2.1 (1.2–4.0)	153
Creatinine (μmol/L)	1.1 (0.8–1.6)	412	1.2 (0.9–2.2)	165
Sodium (mmol/L)	137 (134–140)	331	139 (135–142)	130
Potassium (mmol/L)	4.1 (3.7–4.6)	332	4.2 (3.7–4.7)	130

*ACEi* angiotensin-converting enzyme inhibitor, *ALT* alanine aminotransferase, *ARB* angiotensin II receptor blocker, *AST* aspartate aminotransferase

Details of patient management while in the ICU are summarised in Table 3. Corticosteroids and management of patients in the prone position were more often observed in ACEi/ARB group than non-ACEi/ARB group {prone position: 55% vs. 47%; corticosteroids: 57% vs. 47%}.

**Table 3:** ICU management within the first 28 days following ICU admission

Characteristic	ACEi/ARB n (%)	Available number	Non-ACEi/ARB n (%)	Available number
Antivirals	222 (52)	424	90 (57)	158
Antibiotics	501 (96)	522	185 (94)	196
Corticosteroids	247 (57)	437	78 (47)	167
Heparin	353 (87)	408	125 (84)	148
Prone position	290 (55)	527	93 (47)	198
Mechanical ventilation	506 (96)	528	190 (96)	198
ECMO	79 (15)	527	26 (13)	198
Inhaled nitric oxide	56 (11)	527	22 (11)	198
CRRT	89 (18)	494	38 (21)	185
Vasoactive drugs	304 (62)	487	114 (62)	184
Cardiac assist devices	34 (7)	496	11 (6)	189
Transfused RBC	108 (24)	456	46 (26)	180
Transfused platelets	18 (4)	456	5 (3)	180
Transfused plasma	23 (5)	456	11 (6)	180

*ACEi* angiotensin-converting enzyme inhibitor, *ARB* angiotensin II receptor blocker, *CRRT* continuous renal replacement therapies, *ECMO* extracorporeal membrane oxygenation

**Fig. 1:**
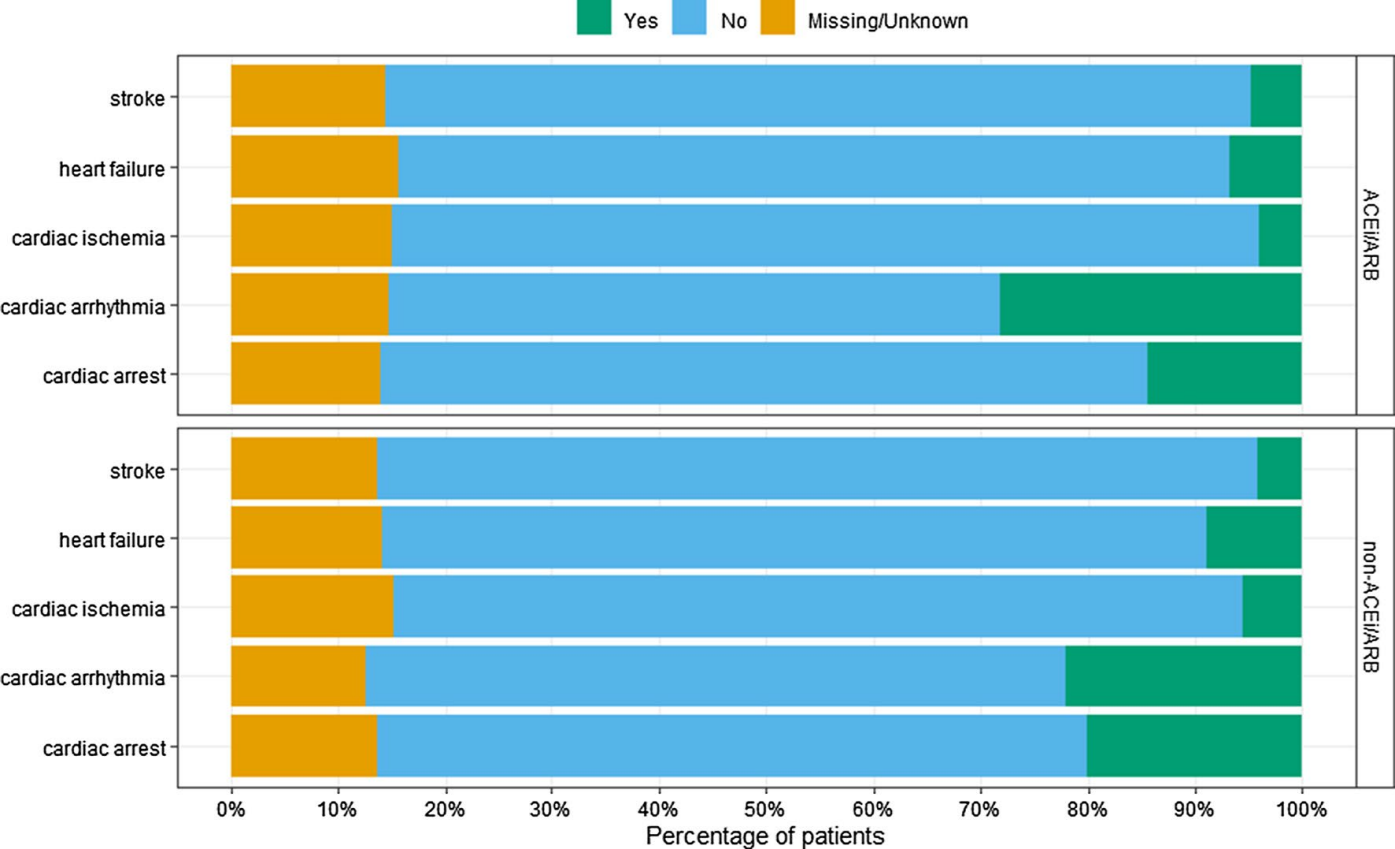
Descriptive statistics for complications recorded at any time during hospitalization, by patient group. *ACEi* angiotensin-converting enzyme inhibitor, *ARB* angiotensin II receptor blocker

Descriptive statistics for complications recorded at any time during hospitalization are summarised in Additional file 2: Table S1 and Fig. 1. Across selected complications, cardiac arrythmias were more frequent in the ACEi/ARB group {ACEi/ARB group vs. non-ACEi/ARB group: 33% vs. 25%, p = 0.068}.

Final outcomes at the end of the study are summarised in Table 4. Death in hospital was observed in 258 patients (48%) in the ACEi/ARB group and in 109 patients (55%) in the non-ACEi/ARB group. Although the main cause of death was similar in the two groups, death due to septic shock was less observed in ACEi/ARB group than non-ACEi/ARB group {6% vs. (14%)}.

**Table 4:** Final outcomes at the end of study

Outcome	ACEi/ARB n (%)	Available number	Non-ACEi/ARB n (%)	Available number
Died in hospital	258 (48)	538	109 (55)	199
Discharged alive from hospital	226 (42)	538	68 (34)	199
Transferred to another facility	7 (1)	538	1 (1)	199
Outcome not finalised	47 (9)	538	21 (11)	199
Recorded cause of death				
Cardiac Failure	16 (6)	258	4 (4)	109
Cerebrovascular accident	3 (1)	258	3 (3)	109
Haemorrhagic shock	3 (1)	258	0 (0)	109
Multi-organ failure	85 (33)	258	29 (27)	109
Respiratory failure	100 (39)	258	47 (43)	109
Septic shock	14 (5)	258	15 (14)	109
Other	19 (7)	258	7 (6)	109
Missing	18 (7)	258	4 (4)	109

Cause of death information is provided for patients known to have died in hospital

*ACEi* angiotensin-converting enzyme inhibitor, *ARB* angiotensin II receptor blocker

### Length of ICU and hospital stay

**Fig. 2:**
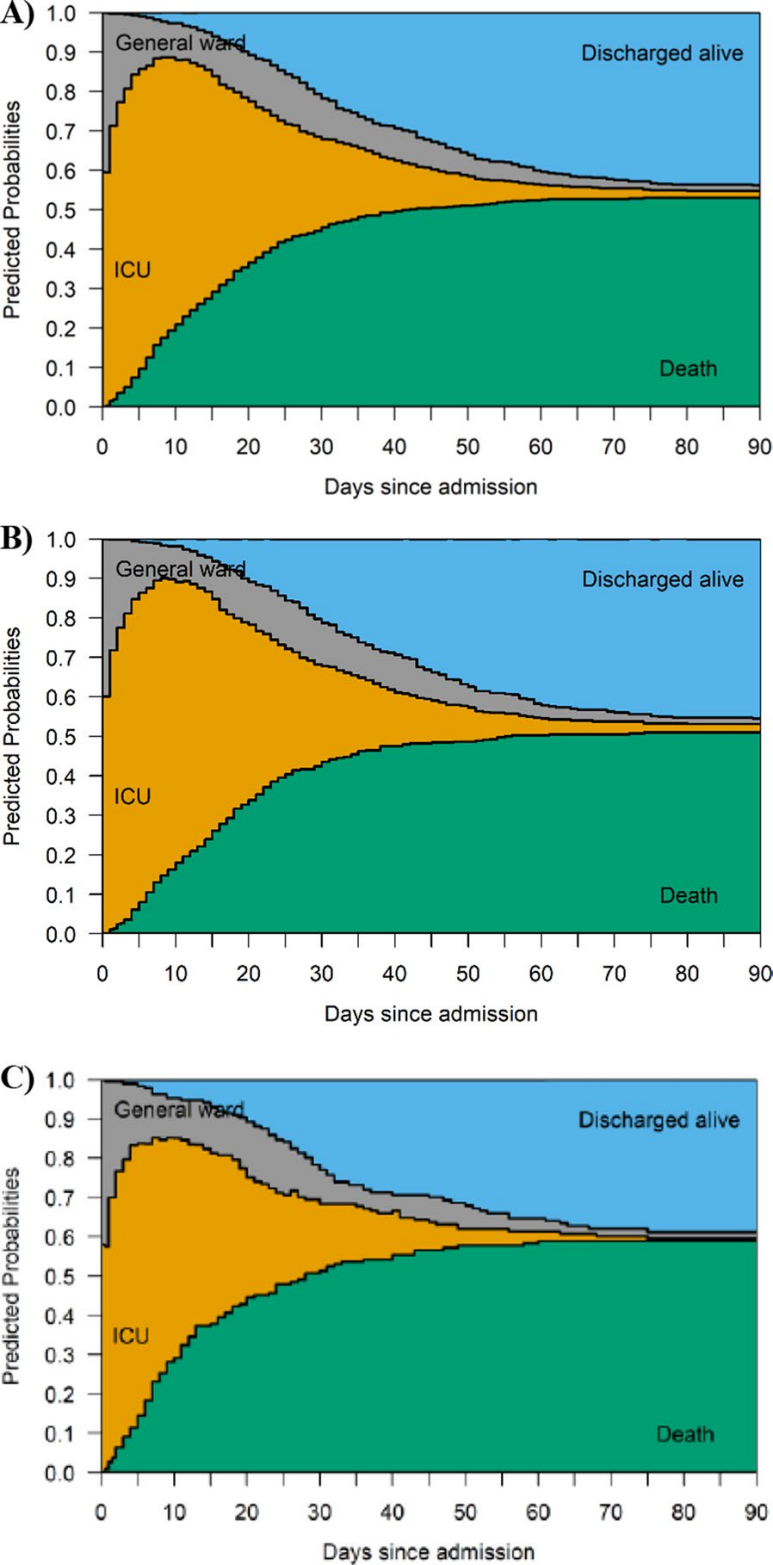
Multistate model results for expected ICU stay and hospital stay up to 90 days from hospital admission. **A** all included patients (n = 737); **B** ACEi/ARB group; and **C** Non-ACEi/ARB group. *ACEi* angiotensin-converting enzyme inhibitor, *ARB* angiotensin II receptor blocker, *CI* confidence intervals, *ICU* intensive care unit. Estimated probabilities at day 0 represent the proportions of patients admitted to ICU on the same day as hospital admission, and the patients who were admitted to the general ward prior to being transferred to the ICU at a later date during hospitalization

Results for expected ICU and general ward stay are summarised in Fig. 2 and Additional file 2: Table S2. Expected lengths of stay were longer in the ACEi/ARB group than non-ACEi/ARB group, with an average time of 21.2 days (95% CI 19.7–22.8) vs. 16.2 days (95% CI 14.1 to18.6) for ICU, and 6.7 days (95% CI 5.9–7.6) vs. 6.4 days (95% CI 5.1–7.9) in general ward, respectively.

### In-hospital mortality

**Fig. 3:**
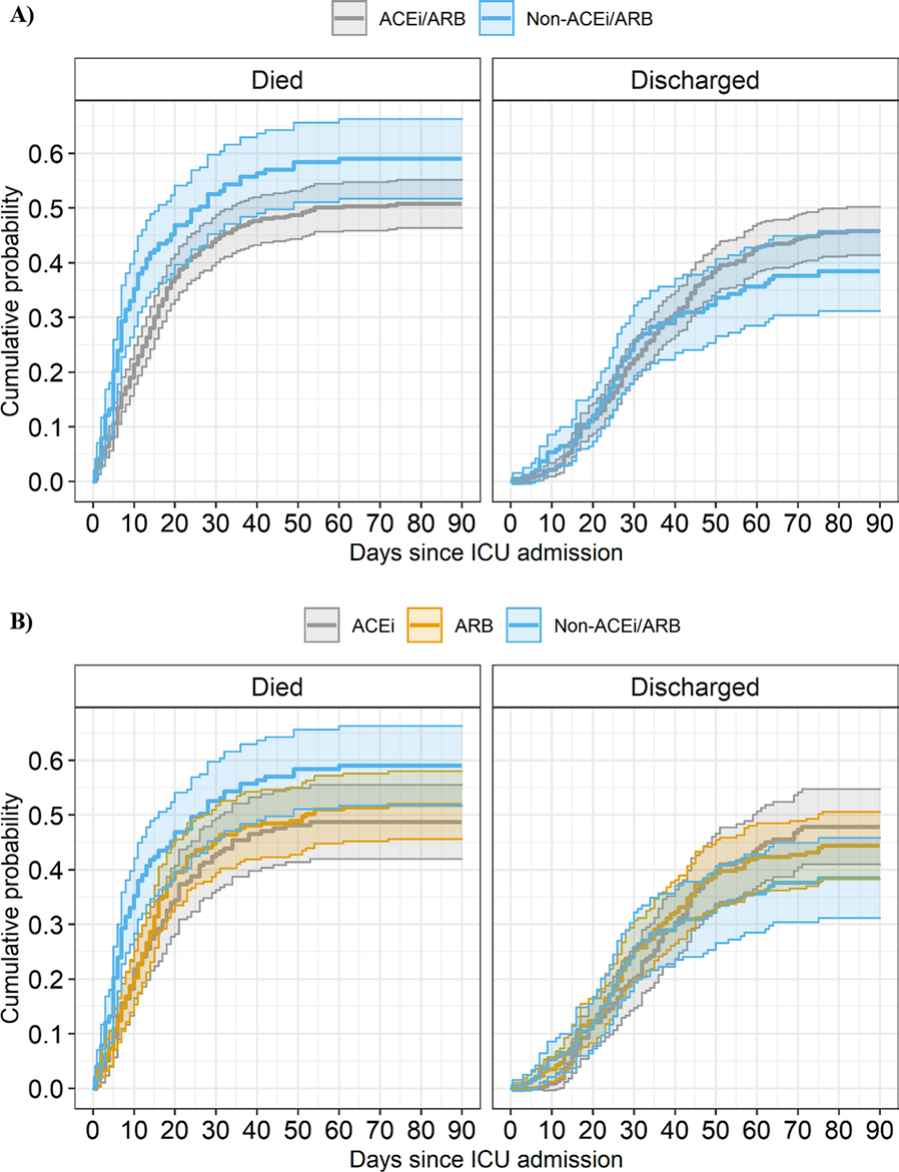
Unadjusted cumulative probabilities of death and discharge from ICU admission. **A** between ACEi/ARB and non-ACEi/ARB groups; **B** between ACEI, ARB and non-ACEI/ARB groups. Results are not adjusted for baseline characteristics. *ACEi* angiotensin-converting enzyme inhibitor, *ARB* angiotensin II receptor blocker, *ICU* intensive care unit

Cumulative incidence of mortality between patient groups indicated differences in mortality up to 30 days from ICU admission (43.5%, SE = 2.2% for ACEi/ARB, and 51.4%, SE = 3.7% for non-ACEi/ARB). By 90 days, expected mortality estimated from the multistate model was 51% (SE = 2.2%) and 59% (SE = 3.6%) for ACEi/ARB and non-ACEi/ARB groups, respectively (Additional file 2: Table S2). Unadjusted cumulative probabilities of death and discharged alive from ICU admission between ACEi/ARB and non-ACEi/ARB groups are shown in Fig. 3.

**Fig. 4:**
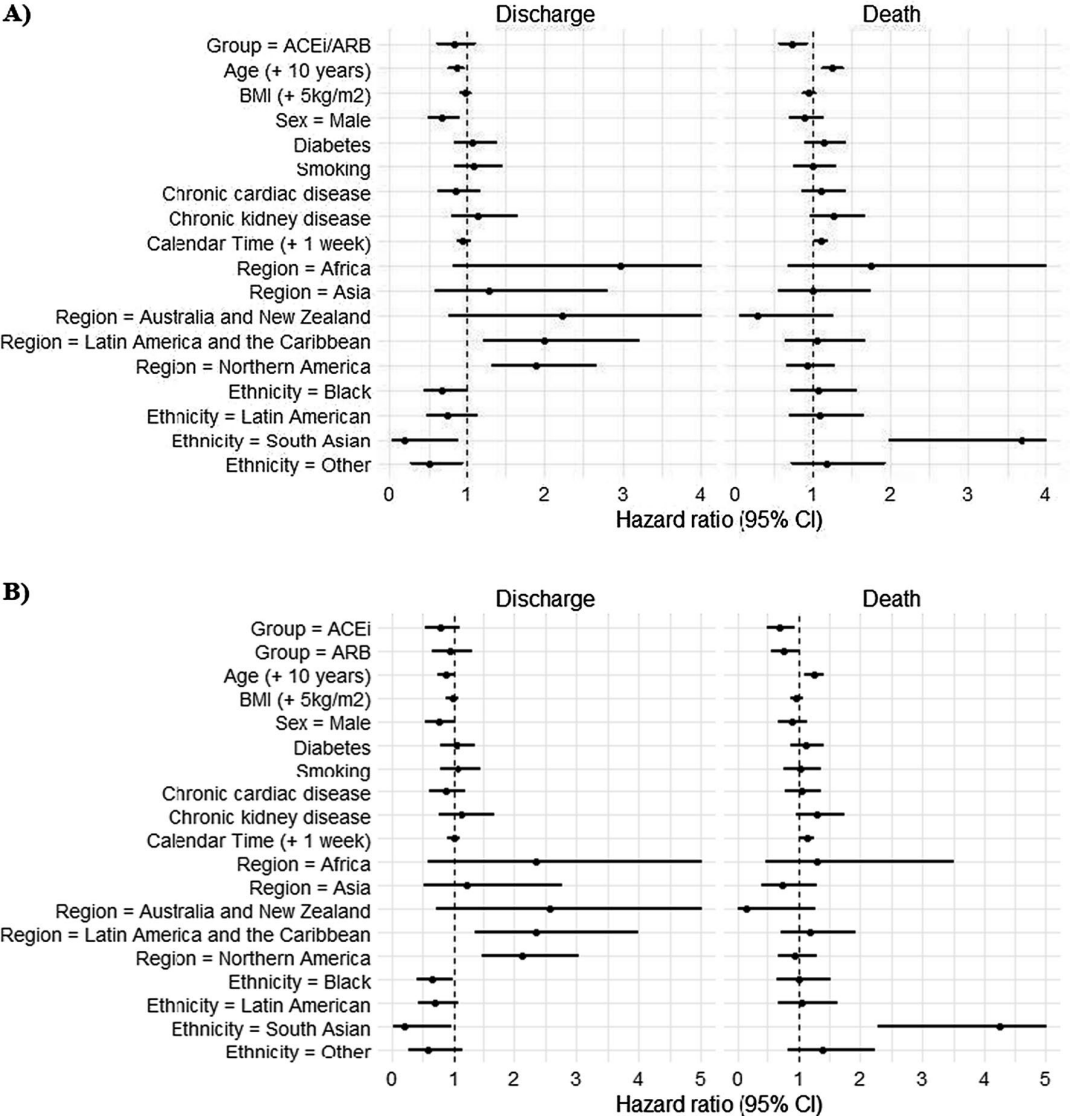
Forest plot of fixed effects included in multistate Cox regression models. **A** Primary analysis with ACEi/ARB versus non-ACEi/ARB groups as a fixed effect; **B** Sensitivity analysis where ACEi/ARB is split into ACEi and ARB groups (n = 41 excluded due to insufficient data to determine stratification). This accounts for competing risks of in-hospital death and hospital discharge up to 90 days from ICU admission. Week of ICU admission indicates calendar time. *ACEi* angiotensin-converting enzyme inhibitor, *ARB* angiotensin II receptor blocker, *BMI* body mass index, *CI* confidence intervals, *ICU* intensive care unit. Terms with an upper confidence limit greater than 5 have been truncated for presentation

Adjusted analyses from multistate Cox regression are presented in Fig. 4 and Additional file 2: Table S3. Modelling indicated ACEi/ARB use was associated with a lower hazard of in-hospital mortality (HR, 0.74, 95% CI 0.58–0.94, p = 0.015), but shared no association with the discharge hazard (HR, 0.86, 95% CI 0.64–1.5, p = 0.307). Adjustment by propensity scores showed that ACEi/ARB use was significantly associated with a lower hazard of death (HR,0.73, 95% CI 0.58–0.93, p = 0.009) (Additional file 2: Table S4). Baseline survival functions stratified by corticosteroid use indicated higher baseline survival among patients who received corticosteroids compared with those that did not, for both death and discharge (Additional file 2: Fig. S3). When ACEi use or ARB use was modelled separately, ACEi returned a statistically significant fixed effects for the hazard of death, compared with the non-ACEi/ARB group; HR 0.69 (95% CI 0.52–0.91, p = 0.01). The corresponding hazards ratio for the ARB group indicated reduced risk of mortality, however, estimate uncertainty did not imply statistical significance (HR 0.77 (95% CI 0.58–1.01, p = 0.062), for ACEi and ARB respectively (Additional file 2: Table S5). Results from propensity score adjustment returned similar estimates for the hazard of death, with hazard ratios equal to 0.74 (95% CI 0.56–0.96, p = 0.026) for the ACEi group and 0.75 (95% CI 0.57–0.97, p = 0.031) for the ARB group (Additional file 2: Table S6).

## Discussion

In this large, international, observational study of prospectively recruited patients with COVID-19 and comorbid hypertension requiring admission to an ICU, the previous use of ACEi/ARB prior to ICU admission was common. In this cohort, we made two important clinical observations. First, the previous use of ACEi/ARB was associated with a reduced risk of in-hospital mortality, compared with not being on either drug class, with the greatest separation between these two groups evident within the first 30 days after admission. Second, despite the improved in-hospital mortality, patients with ACEi/ARB showed longer length of ICU and general ward stay.

Previous use of ACEi/ARB was associated with a reduced risk of in-hospital mortality, compared with not being on either drug class. This is a study to examine mortality of ACEi/ARB vs. non-ACEi/ARB users among critically ill COVID-19 patients specifically managed in the ICU settings. Compared with previous research, our analysis accounted for potential confounders including cardiac comorbidities and corticosteroid use. In a previous study of a cohort of 187 patients with COVID-19, Guo et al. reported that the mortality of RAAS inhibitor users (36.8%; 6 of 19) was higher than that of non-users of RAAS inhibitors (25.6%; 43 of 168)^10^. However, it was uncertain if the higher mortality was related to RAAS inhibitors or a different background, where the RAAS inhibitor group might have a higher rate of comorbidities of cardiovascular disease. A recent meta-analysis involving 28,872 of COVID-19 patients, which showed a significant association between RAAS inhibition and reduced risk of death in the sub-cohort of hypertension [17], provides similar evidence to that reported in our results. Although cardiac arrhythmias were more common in the ACEi/ARB group in a current study corresponding with previous reports [18, 19], it did not impact rates of mortality. Furthermore, the frequency of other cardiac complications during admission (e.g. heart failure, cardiac ischemia and cardiac arrest) was similar between the two groups. As such, the benefit of RAAS inhibitors could be distinct from the well-established prognostic benefit that ACEi/ARB therapy has on cardiovascular diseases [20]. This is potentially related to the anti-inflammatory actions of angiotensin-1–7 and 1–9, both of which are increased by ACEi/ARB through upregulation of ACE-2 [21, 22]. Both have vasodilatory and anti-inflammatory effects through Mas receptors and angiotensin II type 2 receptors, respectively [21, 23]. Some researchers, like Gurwitz et al., even proposed RAAS inhibitors as a tentative treatment for COVID-19 aiming to increase ACE-2 [24] expecting anti-inflammatory effects. In addition, the lower rate of death due to septic shock in ACEi/ARB group in our study, corresponding with another study [9], may be due to the anti-inflammatory effect of ACEi/ARB. In 2020, Hsu et al. conducted a retrospective, propensity score-matched study targeting 3168 sepsis patients with prior use of RAAS inhibitors, but unrelated to COVID-19 infection. They reported that the short-term (up to 90 days) mortality after sepsis was substantially lower among those who were already established on RAAS inhibitor treatment when sepsis occurred [25]. Evidence is limited, but some experimental studies suggested that angiotensin II has a pro-inflammatory effect and causes endothelial and microvascular dysfunctions [26, 27]. RAAS inhibitors may also reduce inflammatory cytokines thus preventing sepsis-related adverse effects by reducing angiotensin II through ACE-2 upregulation.

Despite the improved in-hospital mortality, patients with ACEi/ARB showed a longer length of ICU and general ward stay. In the retrospective study, targeting from non-severe to severe hospitalized COVID-19 patients, Li et al. reported that ACEi/ARB group (n = 115) and non-ACEi/ARB group (n = 247) did not have a significant difference in hospital stay {median 19 days [IQR 13–27] and median 19 days [IQR 11–27], respectively} in contrast to this study. However, when they compared the length of hospital stay of COVID-19 patients with hypertension (n = 362) between survivors (n = 285) and non-survivors (n = 77), the data showed that survivors had a trend to stay longer {median 19 days [IQR 13–26]} than non-survivors {median 15 days [IQR 6–30], p = 0.73} [28]. This may potentially be because the non-survivors could have had more severe disease and died earlier than the survivors. This interpretation is similar to that reported by Rees et al. in a systematic review showing that COVID-19 patients who were discharged alive tended to stay longer than those who died during admission [29].

This is an international report investigating any association between ACEi/ARB use and outcomes in a large group of critically ill COVID-19 patients specifically managed in the ICU settings. The inferences are, therefore, not limited by clinical practices specific to single-country studies. Except for differences such as the rates of corticosteroid administration and prone positioning, the two treatment groups were well matched, in terms of baseline characteristics and the clinical management they received.

Some of the limitations exist in this study. First, as data for our study were collected using a standardized case report form, we were unable to assess the sensitivity of our findings to alternative case definitions when deriving our cohort for analysis. For example, our definition of pre-existing hypertension deviates from the definition by the American College of Cardiology and the American Heart Association (130/80 mmHg) [30]. Similarly, data collected on the current use of antihypertensive therapies was defined as reported use up to two weeks prior to hospital admission. Whilst the use of a standardized case report form allowed for consistent data collection, more detailed information on the timing of medication taken prior to hospitalization and during hospitalization was not available. Our results should therefore be interpreted within the context of patients known to be receiving active treatment for existing hypertension in the lead up to hospitalization for COVID-19 infection, and were likely to have continued receiving therapies as indicated whilst hospitalized. Future studies that account for the continued administration versus discontinuation of antihypertensive therapies as part of COVID-19 management would build upon our findings on the lasting impacts of therapy.

Second, limited data availability on SOFA score and APACHE-II score meant that adjustment for disease severity at time of ICU admission was not possible. However, considering that over 95% of patients in both ACEi/ARB group and non-ACEi/ARB group required mechanical ventilation, it is certain that patients enrolled were critically ill patients requiring ICU management. Third, whilst we adjusted for corticosteroid use in time-to-event analyses, we were unable to investigate the timing and dosage of corticosteroids as these data were not collected by the study case report form. Our results on corticosteroid use were suggestive of a reduced mortality risk in severe COVID-19 patients at least within 28 days following hospital admission, being consistent with previous reports [31, 32]. Without further information on timing and dosage, it is possible that our results overestimate the risk of mortality between study groups, and this should be pursued by future studies. Finally, the voluntary nature of site participation means that our data could be skewed favouring centres with sufficient resources to enter data.

## Conclusions

In critically ill COVID-19 patients with pre-existing hypertension, the previous use of ACEi/ARBs prior to ICU admission was associated with a reduced risk of in-hospital mortality within 90 days from ICU admission, although patients with ACEi/ARB showed longer length of hospital stay. Naturally, the potential survival benefit that we observed requires replication, especially in randomized clinical trials and meta-analyses, to confirm its legitimacy.

## Supplementary Information

**Additional file 1: Document S1.** The rationale and design of the COVID-19-CCC/ECMOCARD registry (Protocol).**Document S2**. Participating sites.**Document S3**. Case report form regarding demographics, comorbidities, medications, laboratory values, complications, and outcomes.**Document S4**. Additional case report form regarding mechanical ventilation and ExtraCorporeal Membrane Oxygenation.

**Additional file 2: Figure S1.** Multistate model structure.**Figure S2**. Flow diagram.**Figure S3**. Stratified baseline survival functions by corticosteroid use from Cox proportional hazards models.**Table S1**. Complications at any time during hospitalization.**Table S2**. Expected length of stay estimated from the multistate model.**Table S3**. Multi-state Cox proportional hazards model. **Table S4**. Propensity score adjusted estimates for the hazards of death and discharge.**Table S5**. Sensitivity analysis, Multi-state Cox proportional hazards model.**Table S6**. Sensitivity analysis, Propensity score adjusted estimates for the hazards of death and discharge.

## Data Availability

The data that support the findings of this study are available from the COVID-19-CCC/ECMOCARD research group but restrictions apply to the availability of these data, which were used under license for the current study, and so are not publicly available. Data are however available from the authors upon reasonable request and with permission of the COVID-19-CCC/ECMOCARD research group.

## Abbreviations

ACE-2: Angiotensin-converting enzyme 2
ACEi: Angiotensin-converting enzyme inhibitor
APACHE-II: Acute Physiology and Chronic Health Evaluation II
ARB: Angiotensin receptor blockers
ARDS: Acute respiratory distress syndrome
BMI: Body mass index
CCB: Calcium channel blocker
CCC: Critical care consortium
CI: Confidence interval
COVID-19: Coronavirus disease-19
ECMO: Extracorporeal membrane oxygenation
ECMOCARD: ExtraCorporeal Membrane Oxygenation for 2019 novel Coronavirus Acute Respiratory Disease
HR: Hazard ratio
ICU: Intensive care unit
IQR: Interquartile ranges
ISARIC: The International Severe Acute Respiratory and Emerging Infection Consortium
MICE: Multiple imputation using chained equations
RAAS: Renin-angiotensin-aldosterone system
SARS-CoV-2: Severe acute respiratory syndrome coronavirus 2
SE: Standard error
SOFA: Sepsis-related Organ Failure Assessment
SPRINT-SARI: Short PeRiod IncideNce sTudy of Severe Acute Respiratory Infection
