# Supplementary material for: Impact of renin–angiotensin–aldosterone system inhibition on mortality in critically ill COVID-19 patients with pre-existing hypertension: a prospective cohort study
Source: BMC Cardiovasc Disord. 2022 Mar 23;22:123. doi: 10.1186/s12872-022-02565-1 (PMC8942148; doi:10.1186/s12872-022-02565-1)
Supplement: Supplementary file 1 — Additional file 1: Document S1. The rationale and design of the COVID-19-CCC/ECMOCARD registry (Protocol).Document S2. Participating sites.Document S3. Case report form regarding demographics, comorbidities, medications, laboratory values, complications, and outcomes.Document S4. Additional case report form regarding mechanical ventilation and ExtraCorporeal Membrane Oxygenation. [file 12872_2022_2565_MOESM1_ESM.pdf]

SUPPLEMENTAL MATERIAL

(Additional File 1)

Document S1: The rationale and design of the COVID-19-CCC/ECMOCARD registry (Protocol)

Document S2: Participating sites

Document S3: Case report form regarding demographics, comorbidities, medications, laboratory values, complications, and outcomes

Document S4: Additional case report form regarding mechanical ventilation and ExtraCorporeal Membrane Oxygenation

Document S1. The rationale and design of the COVID-19-CCC/ECMOCARD registry (Protocol)

CONFIDENTIAL

Covid-19 Critical Care Consortium Observational Study

Incorporating the
ExtraCorporeal Membrane Oxygenation for 2019 novel Coronavirus Acute Respiratory Disease

v. 1.2.8

Chief Investigators:

A/Prof Gianluigi LI BASSI
University of Queensland
Level 3 | Clinical Sciences Building
Chermside Qld 4032
Australia
T:+61 7 3139 6880
Mobile: +61 0421273217
Email: g.libassi@uq.edu.au

Dr. Jacky SUEN
University of Queensland
Level 3 | Clinical Sciences Building
Chermside Qld 4032
Australia
T:+61 7 3139 6880
Mobile: +61 400128961
Email: j.suen1@uq.edu.au

Prof. John Fraser
President Elect Asian-Pacific Extracorporeal Life Support
University of Queensland
Level 3 | Clinical Sciences Building
Chermside Qld 4032 – Australia

Prof Heidi Dalton
Inova Fairfax Hospital
3300 Gallows Rd
Falls Church, VA 22042-3307
United States
T:+1 703-776-6041
Email: heidi.dalton26@gmail.com

Prof Adrian BARNETT
Queensland University of Technology
Faculty of Health,
School - Public Health and Social Work,
Research - Public Health
T: +61 7 3138 6010
Email: a.barnett@qut.edu.au

Dr Sally SHRAPNEL
University of Queensland
School of Mathematics and Physics
Faculty of Science
Australia
T: +61 7 336 56931
Email: s.shrapnel@uq.edu.au

ECMOCARD Research Coordinator:

Research Program Support Team

Critical Care Research Group

Level 3 | Clinical Sciences Building

The Prince Charles Hospital

Chermside Qld 4032

Australia

Email: ecmocard@health.qld.gov.au

ECMOCARD Coordinating Centres:

Extracorporeal Life Support Organisation And Asia-Pacific Life Support Organisation

Critical Care Research Group

Level 3 | Clinical Sciences Building

The Prince Charles Hospital

Chermside Qld 4032

Australia

Email: ecmocard@health.qld.gov.au

COVID-19 Critical Care Consortium Steering Committee

President: Robert Bartlett, Department of Surgery, University of Michigan, Ann Arbor, MI, USA.

Committee:

1. **Aidan Burrell,** Australian and New Zealand Intensive Care Research Centre/ The Alfred Hospital/Monash University, Melbourne, Australia.
2. **Daniel Brodie**, Columbia University College of Physicians and Surgeons/New York-Presbyterian Hospital, New York, USA
3. **Davide Chiumello**, San Paolo Hospital and University of Milan, Milan, Italy
4. **Heidi Dalton**, INOVA Fairfax Hospital, Falls Church, Virginia, USA
5. **John Fraser**, Critical Care Research Group, The Prince Charles Hospital, Chermside, Australia
6. **Alyaa Elhazmi,** King Faisal Specialist Hospital and Research Centre, Riyadh, Saudi Arabia
7. **Carol Hodgson**, Australian and New Zealand Intensive Care Research Centre/ The Alfred Hospital/Monash University, Melbourne, Australia.
8. **Trieu Huynh**, Hospital for Tropical Diseases, 764 Vo Van Kiet, Ho Chi Minh City, Vietnam
9. **Shingo Ichiba**, Department of Surgical Intensive Care Medicine, Nippon Medical School Hospital.
10. **John Laffey**, Regenerative Medicine Institute (REMEDI) at CÚRAM Centre for Research in Medical Devices, Biomedical Sciences Building, National University of Ireland Galway, Galway, Ireland; Department of Anaesthesia and Intensive Care Medicine, Galway University Hospitals, and School of Medicine, Clinical Sciences Institute, National University of Ireland, Galway, Ireland
11. **Gianluigi Li Bassi**, Critical Care Research Group, The Prince Charles Hospital, Chermside, Australia
12. **Carlos Luna**, Department of Medicine, Pulmonary Diseases Division, Hospital de Clínicas, Universidad de Buenos Aires, Buenos Aires, Argentina.
13. **Srinivas Murthy**, BC Children's Hospital, Department of Pediatrics, University of British Columbia, Vancouver, Canada.
14. **Alistair Nichol**, Critical Care Medicine in University College Dublin, Director of the Irish Critical Care-Clinical Trails Network, Consultant in St Vincent's University Hospital, Dublin
15. **Jacky Suen**, Critical Care Research Group, The Prince Charles Hospital, Chermside, Australia
16. **Mark Ogino,** Department of Paediatrics, Division of Neonatology, Nemours Alfred I duPont Hospital for Children, Wilmington, DE, USA; Sidney Kimmel Medical College, Thomas Jefferson University, Philadelphia, PA, USA.
17. **Antoni Torres,** Hospital Clinic and University of Barcelona, Barcelona, Spain

18. **Antonio Pesenti,** Fondazione IRCCS Ca' Granda Ospedale Maggiore Policlinico, Dept of Pathophysiology and Transplantation University of Milan, Milano Italy.
19. **Pauline Yeung,** Division of Respiratory and Critical Care Medicine, Department of Medicine, The University of Hong Kong/ L345, Adult Intensive Care Unit, Queen Mary Hospital, 102 Pokfulam Road, Hong Kong.
20. **Eddy Fan**, Interdepartmental Division of Critical Care Medicine, and Institute of Health Policy Management and Evaluation University of Toronto, Canada

Summary

**Scientific Title**	**Covid-19 Critical Care Consortium**<br>**Incorporating the**<br>**E**xtra**C**orporeal **M**embrane **O**xygenation for 2019 novel **C**oronavirus **A**cute **R**espiratory **D**isease (ECMOCARD)
**Study Design**	Prospective/Retrospective multi-centre short period incidence observational study of patients in participating hospitals and intensive care units (ICUs) with 2019 novel coronavirus (COVID-19).
**The Collaborative**	**In response to the COVID-19 outbreak and to assist in pandemic planning both locally and globally, a research collaborative has been assembled.** The collaborative consists of investigators from the Asia-Pacific extracorporeal life support organization (APELSO) in collaboration with centres within the SPRINT-SARI and ISARIC Network. In Australia, this study will be also complemented through collaboration with the “National registry on the treatment and outcomes of patients requiring ECMO” (EXCEL Registry).
**Study Aim and Objectives**	To describe clinical features; severity of pulmonary dysfunction; incidence of ICU admission, coagulatory and thrombotic derangement, cardiac dysfunction, neurological impact, kidney injury, use of mechanical ventilation, ECMO technical characteristics; duration of ECMO; complications; and survival of patients with COVID-19.
**Inclusions/Exclusions**	All patients admitted to ICU with clinical suspicion or laboratory confirmed COVID-19 infection by real-time PCR and/or next-generation sequencing will be included.

	Patients receiving mechanical ventilation or ECMO for other concomitant causes will be excluded.
**Consent**	Given the negligible risk associated with this study and the timely nature in which the data needs to be collected, a waiver of consent is sought.
**Study Setting**	International multi-centre study, conducted in all collaborating hospitals/ICU-based research networks globally.
**Sample Size**	All patients with confirmed COVID-19 infection admitted to ICUs at the collaborative centres
**Study Start Date**	From the commencement of COVID-19 global epidemic
**Study Duration**	Until completion of COVID-19 global epidemic, as judged by the World Health Organization
**Data collection processes**	Patients will be studied from time of ICU admission until hospital discharge or up to 28 days post ICU admission, whichever occurs later. All clinical information will only be recorded if taken as part of routine clinical practice at each site. Only de-identifiable data will be submitted centrally (REDCap hosted at Oxford University for International centres and at Monash University for Australian centres). A specific Case Report Form (CRF) will be used by participating sites to collect data set of ICU, mechanical ventilation and ECMO data. An optional Basic CRF will also be available for sources with limited resources for data collection. Data for COVID-19 Critical Care Consortium and ISARIC/SPRINT SARI observational studies will be concomitantly collected. Data will be recorded into REDcap through standard data collection or interactive augmented human experience via digital interaction by voice or touch monitors or digital transcription of CRF hard copies. In Australia, patients concomitantly included into the EXCEL registry, EXCEL data will be requested to complement COVID-19 Critical Care Consortium observational study data and reduce daily workload.

Table of contents

COVID-19 CRITICAL CARE CONSORTIUM STEERING COMMITTEE .... ERROR! BOOKMARK NOT DEFINED.

SUMMARY 4

TABLE OF CONTENTS 6

INTRODUCTION 8

INTERNATIONAL SEVERE ACUTE RESPIRATORY AND EMERGING INFECTION CONSORTIUM (ISARIC) 8
SHORT PERIOD INCIDENCE STUDY OF SEVERE ACUTE RESPIRATORY INFECTION (SPRINT-SARI) 9
CORONAVIRUSES 10
2019 NOVEL CORONAVIRUS (COVID-19) 10

OBJECTIVES 14

HYPOTHESIS 14
AIMS 14

MATERIALS AND METHODS 14

STUDY DESIGN 14
RESEARCH CENTRES 15
STUDY POPULATION 15
Inclusion Criteria *15*
Exclusion Criteria *15*
Co-enrolment *16*

ETHICS 16

GUIDING PRINCIPLES 16
COMPLY WITH ALL LOCAL REQUIREMENTS 16

CONFIDENTIALITY OF PATIENT DATA 17

RULE OF TRANSFER 17
INTERNATIONAL WAIVER OF INFORMED CONSENT 18
INFORMED CONSENT IN AUSTRALIA 18

DATA COLLECTION 18

ISARIC DATA COLLECTION 18
COVID-19 CRITICAL CARE CONSORTIUM OBSERVATIONAL STUDY DATA COLLECTION 19
COVID-19 CRITICAL CARE CONSORTIUM OBSERVATIONAL STUDY BASIC DATA COLLECTION (OPTIONAL) 20
COAGULATION DISORDERS AND THROMBOSIS SUB-STUDY DATA COLLECTION (OPTIONAL) 22
NEUROLOGY SUB-STUDY DATA COLLECTION (OPTIONAL) 23
CARDIAC SUB-STUDY DATA COLLECTION (OPTIONAL) 23
ACUTE KIDNEY INJURY SUB-STUDY DATA COLLECTION (OPTIONAL) 24
SCREENING LOG 30
DATA QUALITY 30
DATA MANAGEMENT 31
MONITORING 32
COLLECTED PARAMETERS 32
Demographics and Medical History *32*
COVID-19 infection *32*
Clinical parameters upon commencement of invasive mechanical ventilation *33*
Daily assessment of clinical parameters during invasive mechanical ventilation *33*
Clinical features before commencement of ECMO *34*

ECMO characteristics ... 35
ECMO adverse effects ... 35
Daily assessments for Coagulation Disorders and Thrombosis Sub-study ... 35
Main outcomes ... 36
Sub-studies ... 36

DATA ANALYSIS ... 38

REFERENCE LIST ... 39

REGULATION, ETHICS AND GOVERNANCE ... 42

CONFLICT OF INTEREST ... 42
DATA COLLECTION AND SITE MONITORING PLAN ... 42
Data Collection ... 42
Site Monitoring ... 42
Compensations ... 43
DATA ACCESS ... 43

FEASIBILITY ... 43

DISSEMINATION AND PUBLICATION ... 43

PUBLICATION POLICY ... 43
AUTHORSHIP POLICY ... 44

Introduction

The ExtraCorporeal Membrane Oxygenation for 2019 novel Coronavirus Acute Respiratory Disease (ECMOCARD Trial) will be carried out within the network and web-based case collection forms of the ISARIC consortium's SPRINT-SARI study and in Australian and New Zealand centres, upon conclusion of the epidemics, potentially complemented through the study "A comprehensive national registry on the treatment and outcomes of patients requiring ECMO" (EXCEL Registry).

International Severe Acute Respiratory and Emerging Infection Consortium (ISARIC)

The International Severe Acute Respiratory and Emerging Infection Consortium (ISARIC) was formed in 2011, in response to global recognition of the unmet need for timely and effective clinical research during outbreaks of emerging infectious disease with epidemic or pandemic potential. ISARIC represents a new paradigm for effective, coordinated, and timely collaborative clinical research during rapidly emerging threats to public health. It is collaboration among clinicians, clinical researchers, epidemiologists, ethicists, statisticians, laboratory-based clinicians, basic scientists, and public health experts. The mission of ISARIC is to develop operational readiness and to co-ordinate the conduct of essential clinical research to characterise and respond to new epidemic or pandemic infectious disease threats, thereby informing and guiding evidence-based optimal management. ISARIC is facilitating the coordination of SPRINT-SARI, which supports ISARIC's goal of improving the effectiveness of clinical researching globally during a pandemic by:

1. Establishing protocols, with standardised definitions and study methods, for conducting time-critical research during outbreaks of emerging infectious diseases;
2. Coordinating a large number of globally diversified hospitals and/or ICU-based networks with pre-existing ethics, administrative, regulatory and logistics in place, sufficient to implement study protocols, especially including regions where this type of clinical research has traditionally not been performed;
3. Identifying and solving barriers to pandemic research, including those identified in SPRINT-SARI;
4. Studying SARI globally, providing evidence on SARI microbiology, treatment and outcome in both resource-rich and resource-poor settings;
5. Allowing ISARIC to evaluate its research capacity and capabilities; and

6. Assisting ISARIC to maintain network stakeholders during inter-pandemic periods.

Short PeRiod IncideNce sTudy of Severe Acute Respiratory Infection (SPRINT-SARI)

Severe acute respiratory infection (SARI) continues to be of major relevance to public health worldwide. In the last 10 years there have been multiple SARI outbreaks around the world. The 2009 H1N1 pandemic was estimated to result in more than 200,000 respiratory deaths globally[1–3]. The World Health Organization (WHO) defines SARI as an acute respiratory infection of recent onset (within 10 days) requiring hospitalisation, manifested by fever (≥38oC) or a history of fever and cough [4–6]. There is international consensus that it is important to undertake observational studies of patients with SARI as an essential component of pandemic and epidemic research preparedness. The primary aim of the SPRINT-SARI study was to establish a research response capability for future epidemics / pandemics through a global SARI observational study. The secondary aim of this study was to describe the clinical epidemiology and microbiology profiles of patients with SARI. The tertiary aim of this study was to assess the Ethics, Administrative, Regulatory and Logistic (EARL) barriers to conducting pandemic research on a global level. SPRINT-SARI was designed as a multi-centre, prospective, short period incidence observational study of patients in participating hospitals and intensive care units (ICUs) with SARI. The study period was planned to occur, in both Northern and Southern hemispheric winters. The study period comprised a 5 to 7-day cohort study in which patients meeting a SARI case-definition, who are newly admitted to the hospitals/ICUs at participating sites, will be included in the study. The study was planned to be conducted in 20 to 40-hospital/ ICU-based research networks globally. All clinical information and sample data were planned to only be recorded if taken as part of the routine clinical practice at each site and only fully anonymised and re-identifiable data will be submitted centrally. The primary outcome of SPRINT-SARI was to test the feasibility of conducting a global study of SARI.

Secondary Outcomes:

1. Incidence of SARI
2. Disease severity and risk factors for severe disease due to SARI
3. Case Fatality Proportion of SARI
4. Duration of ICU/hospital stay due to SARI
5. Microbiology of SARI, including variability in testing

6. Treatments received during hospitalization for SARI

7. Evaluate impact on incidence of alternative case-definitions of SARI

8. Evaluate the operational characteristics of this study, including CRF, Completion Guidelines, and entry criteria to provide information by which iterative improvement in study design can be achieved.

9. Explore the feasibility of extrapolation of results obtained at participating sites to population levels

Coronaviruses

Coronaviruses are a family of enveloped, single-stranded, positive-strand RNA viruses classified within the Nidovirales. Coronaviruses may infect mammals and birds, triggering respiratory, enteric, hepatic, and neurologic diseases[7]. Six coronavirus species are known to cause human disease. The coronaviruses 229E, OC43, NL63, and HKU1 are prevalent worldwide and most commonly cause only marginal respiratory symptoms. Two other strains, the severe acute respiratory syndrome coronavirus (SARS-CoV) and Middle East respiratory syndrome coronavirus (MERS-CoV) have originated from animal to human transmission and have caused more serious, sometimes fatal, respiratory illnesses. In previous years, SARS-CoV[8,9] and MERS-CoV[10,11], have caused serious respiratory infections, with mortality rates of 10% for SARS-CoV[12] and 37% for MERS-CoV[13].

2019 Novel Coronavirus (COVID-19)

In late December, 2019, in Wuhan, Hubei, China, a new respiratory syndrome emerged with clinical signs resembling viral pneumonia and person-to-person transmission[14]. Prompt diagnostic methods, through deep sequencing analysis from lower respiratory tract samples, corroborated emergence of a novel coronavirus, namely the 2019 novel coronavirus (COVID-19). In particular, Na Zhu and collaborators[15] were able to isolate the virus from bronchoalveolar lavage (BAL) from patients with pneumonia of unknown cause, who were in Wuhan on December 21, 2019 or later, and who had been present at the Huanan Seafood Market. RNA extracted from BAL fluid from the patients was used as a template to clone and sequence a genome using a combination of Illumina sequencing and nanopore sequencing. More than 20,000 viral reads from individual specimens were obtained, and most contigs matched to the genome from lineage B of the genus betacoronavirus —

showing more than 85% identity with a bat SARS-like CoV (bat-SL-CoVZC45, MG772933.1) genome. Virus isolation from the clinical specimens was performed with human airway epithelial cells and Vero E6 and Huh-7 cell lines. 2019-nCoV–infected human airway epithelial cultures were examined with light microscopy and with transmission electron microscopy 6 days after inoculation. Cytopathic effects were observed 96 hours after inoculation on surface layers of human airway epithelial cells and lack of cilium beating was seen with light microcopy (Fig. 1).

Figure 1

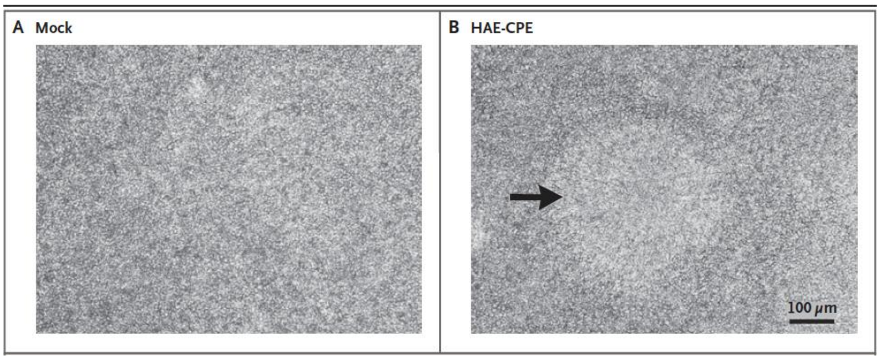

Figure 1: Cytopathic effect of the novel coronavirus, as reported in previous publication[15]

Through transmission electron microscopy, the authors were able to image the COVID-19 particles, that generally appeared spherical, of 60 to 140 nm, with some pleomorphism and distinctive spikes, about 9 to 12 nm (Fig. 3), and gave virions the appearance of a solar corona. This morphology corroborated the Coronaviridae family.

Figure 2

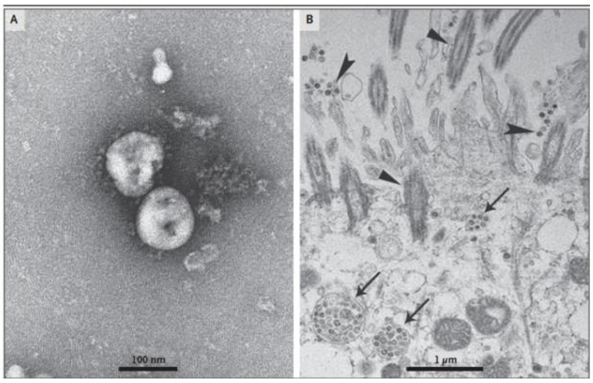

Figure 2: A: COVID-19 particles are depicted. B: COVID-19 in human airway epithelium, as reported in previous publicaition[15].

Finally, investigators carried out inclusive phylogenetic analysis that showed that COVID-19 falls into the genus betacoronavirus, which includes coronaviruses as SARS-CoV, bat SARS-like CoV, and others from humans, bats, and other wild animals.

Thus far, more than 111,000 confirmed cases, including health-care workers, have been identified worldwide, and several exported cases have been confirmed in other provinces in China, Thailand[16], Japan[17], South Korea[18], Germany, Italy[19], France, Iran[20], USA[21] and many other countries[22]. An early case report in 41 patients with laboratory-confirmed COVID-19 infection in Wuhan has been reported[23]. The median age of the patients was 49 years and mostly men (73%). Among those, 32% were admitted to the ICU because they required high-flow nasal cannula or higher-level oxygen support measures to correct hypoxaemia. Less than half had underlying diseases, including diabetes (20%), hypertension (15%), and cardiovascular diseases (15%). On admission, 98% of the patients had bilateral multiple lobular and subsegmental areas of consolidation (Figure 3)[24].

Figure 3

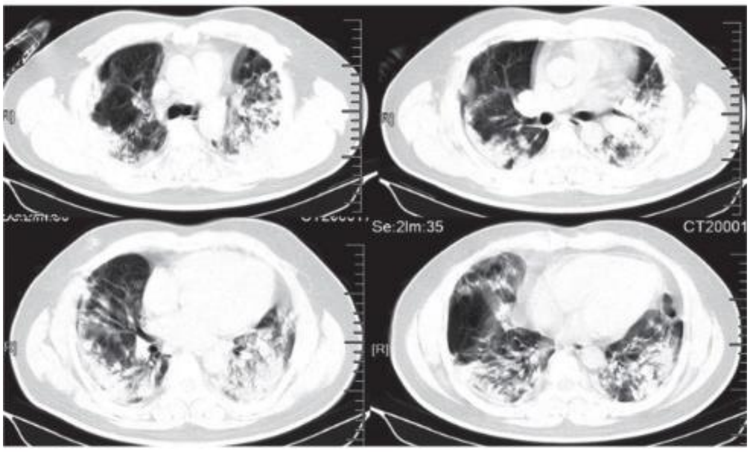

Figure 3 Caption: Transverse chest CT images from a 40-year-old man showing bilateral multiple lobular and subsegmental areas of consolidation on day 15 after symptom onset. Transverse chest CT images from a 53-year-old woman showing bilateral ground-glass opacity and subsegmental areas of consolidation on day 8 after symptom onset, adapted from[23]

Importantly, acute respiratory distress syndrome (ARDS) developed in 29% of the patients, while acute cardiac injury in 12%, and secondary infection in 10%. Invasive mechanical ventilation was required in 10% of those patients, ***and two of them (5%) had refractory hypoxaemia and received extracorporeal membrane oxygenation (ECMO)***.

In a later retrospective report by Wang and collaborators[25], clinical characteristics of 138 patients with COVID-19 infection were described. Those patients were admitted at Zhongnan Hospital of Wuhan University in Wuhan, China, from January 1 to January 28, 2020. The median age was 56 years and clinical signs of the infection comprised fever (98.6%), fatigue (69.6%), and dry cough (59.4%). Interestingly, lymphopenia occurred in 70.3% of the patients, prolonged prothrombin time 58%, and elevated lactate dehydrogenase 39.9%. ICU admission was required in 26.1% of the patients for acute respiratory distress syndrome (61.1%), arrhythmia (44.4%), and shock (30.6%). Among these patients, 11.1% received high-flow oxygen therapy, 41.7% non-invasive ventilation, and 47.2% invasive ventilation. ***ECMO support was needed in 11% of the patients admitted to the ICU***. During the period of follow-up, overall mortality was 4.3%.

Objectives

Hypothesis

We hypothesize that a significant percentage of patients with COVID-19 infection will require admission to the intensive care unit, mechanical ventilation and ECMO for refractory hypoxemia, in addition a substantial proportion of patients will present coagulation disorders and thrombosis.

Aims

This is a multi-centre international study in patients with COVID-19 who require admission to the intensive care unit, mechanical ventilation and/or ECMO to characterize the following features:

1. Incidence of ICU admission, use of mechanical ventilation and ECMO
2. Risk factors
3. Clinical features
4. Coagulation disorders and thrombosis
5. Severity of respiratory failure
6. Need for non-invasive and invasive mechanical ventilation and ECMO
7. Settings of invasive mechanical ventilation
8. ECMO technical characteristics
9. Duration of ECMO
10. Complications
11. ICU survival
12. Hospital survival.
13. Requirements and the time frame for approvals in each participating network region

Materials and Methods

Study Design

This is an international multi-centre, prospective/retrospective observational study of patients in participating hospitals and ICUs with COVID-19 infection. The study will be conducted at 20 to 90 hospital networks globally and will aim to recruit as many patients as possible. The aim is to recruit all eligible patients at each study location and there is no maximum number of patients that can be recruited from any one site. Patients will be studied from time of ICU admission up to

28 days or until hospital discharge, whichever occurs later. Information will be collected on demographics, co-existing illnesses, severity of illness, source and type of clinical specimens (upper versus lower respiratory tract and collection date), results of microbiological tests. ECMOCARD will specifically focus on collecting data of mechanical ventilation and ECMO and administration of other major therapies (including vasoactive therapies, hypoxaemia rescue therapies, and dialysis), administration of antibiotics and antivirals (and adjunctive therapies, e.g. immunomodulators, corticosteroids) and outcomes at ICU (if applicable), hospital discharge and 28 days. Furthermore, there will be additional optional CRFs to collect data for the following sub-studies (further information is provided in the Data Collection section of this protocol):

- Coagulation Disorders and Thrombosis sub-study
- Neurology sub-study
- Cardiac sub-study
- Acute Kidney Injury sub-study

Research centres

This is a collaborative effort among investigators of the Asia-Pacific Extracorporeal Life Support Organization (APELSO) in collaboration with centres within the SPRINT-SARI and ISARIC Network.

Study Population

We plan to recruit as many patients as possible of the patients with COVID-19 infection admitted to the ICU, in as many locations as possible, who meet the inclusion criteria with no-exclusion criteria at the participating sites. It is anticipated that each participating Institution could contribute between 5 and 50 patients. Each site's recruitment will be determined by the incidence of the disease during the study period, and their ability to collect the required data.

Inclusion Criteria

1. Clinical suspicion or laboratory-confirmed COVID-19 infection by real-time PCR and/or next-generation sequencing
2. Admission to an intensive care unit

Exclusion Criteria

1. Patients treated with mechanical ventilation for other concomitant causes

2. Patients treated with ECMO for other concomitant causes

Co-enrolment

This is an observational study. Co-enrolment with other studies including interventional clinical trials is accepted.

Ethics

Guiding Principles

The Chief Investigators and study management team are responsible for ensuring the study is performed in accordance with the protocol. This study is to be performed in accordance with the ethical principles of the Declaration of Helsinki (June 1964, most recently amended in October 2013), and the most recent, relevant ethical conduct of research guidelines published in the country of the participating site. The Principal Investigator at each site is responsible for maintenance of a securely held enrolment log linking the patient hospital record number and the study number as per their countries research guidelines.

Comply with all local requirements

National or regional Co-ordinators in their defined location will be responsible for clarifying the requirements for ethics approval. It is the responsibility of the site Chief Investigator and Research Co-ordinator to ensure ethics approval has been granted prior to commencing the study and all local requirements are addressed. Each participating site will require ethics approval for this protocol and data collection of the ECMOCARD and ISARIC SPRINT-SARI CRF (RAPID, CORE, SUPPLEMENTARY TO CORE, DAILY and EPIDEMIOLOGY) and any other study documents relevant to their region. When possible, each participating study site will be supported by the ECMOCARD, Project Officer with their application. The Principal Investigator will produce progress reports, and any other required documentation for the local independent Ethics Committee in accordance with their guidelines. It is the responsibility of the Chief Investigator at each participating hospital to keep an up to date record of all correspondence and applicable documentation with the local Independent Ethics Committee. We will be collecting data on the requirements and the time frame for approvals in each participating network region.

Confidentiality of patient data

No identifying data will be entered into the central database. Participants' names will not be collected, and confidentiality of information in medical records will be preserved. The confidentiality of the participant will be maintained unless disclosure is to comply with the law. To adhere to international ethical review board requirements and facilitate global ECMOCARD and SPRINT-SARI ISARIC data polling/sharing the CLiRes Data Management System will convert all dates entered (DD/MM/YYYY) into the eCRF into a re-identifiable format (D1, D2) at a system level. The original entered data (DD/MM/YYYY) will only be accessible by the site Research Co-ordinator and the site Principal Investigator using their unique database account details. ***In Australia, re-identifiable data will be entered into a central REDCap database hosted by Monash University and harmonised with the SPRINT-SARI study.***

Rule of Transfer

It is proposed that if a patient is transferred from a facility participating in ECMOCARD and SPRINT-SARI to another facility that is also participating, the patient's previously allocated patient ID number will be documented in the CRF completed by the receiving hospital at time of admission. All sites participating in SPRINT-SARI will be asked to include a ECMOCARD and SPRINT-SARI study information sheet in the patients transferring documents, notifying the new hospital of the patient's inclusion in ECMOCARD and SPRINT-SARI, the patients re-identifiable participation number, the contact details of the Principle Investigator of ECMOCARD and SPRINT-SARI in the country and the ECMOCARD and SPRINT-SARI coordinating centre. If you are unsure if a patient has previously been enrolled in ECMOCARD and SPRINT-SARI please check to see if the patients transferring hospital and ward/unit are included in the participating sites list on the ECMOCARD and SPRINT-SARI website (www.sprintsari.org). Please use the patients existing ECMOCARD and SPRINT-SARI participant number at the new hospital when entering data into the paper and/or eCRF. Sites will not have access to any data collected outside their hospital; it is the responsibility of each hospital to enter data pertaining to their component of the patient's hospital admission. If a patient is transferred to a non-participating hospital, there will be no further data collection.

International waiver of informed consent

It is expected that this study will not require individual patient consent. This study is in effect a large-scale clinical audit, as all data is already recorded as part of routine clinical care, therefore justifying participant enrolment using a waiver of consent. Waiver of consent may be available for studies that submit only re-identifiable information and where involvement in the research carries no more than low risk. Any location that deems individual consent necessary can use potential forms reported in the Appendix A. In particular, only in patients who meet the inclusion/exclusion criteria, informed consent will be obtained directly from the patient, either before the study or retrospectively in case the patient is unconscious at the time of enrolment. If the patient is unable to provide a consent form upon admission, informed consent will be obtained by his/her next of kin.

Informed Consent in Australia

In Australia all patients admitted to the ICU and meeting all inclusion and no exclusion criteria will be included in ECMOCARD observational study. Their hospital data will be included under a waiver of consent, in line with the National Statement (chapter 2.3) and the NHMRC *Ethical Considerations in Quality Assurance and Evaluation Activities, 2014.*

Data for ECMOCARD and SPRINT SARI observational study will be concomitantly collected. In addition, to minimise workload for site staff, whenever possible, EXCEL data will be requested to complement ECMOCARD data. SPRINT-SARI and EXCEL have both been approved to recruit patients under a waiver of consent. Yet, it is important to emphasize that ethics approval certificate for Project 202/16 has the following special condition: “A waiver of the requirement for consent was granted for the collection and use of identifiable information during relevant epidemics and pandemics. An opt-out approach will be used at all other times.”

Data Collection

ISARIC Data Collection

As detailed in following paragraphs, we will collect data prospectively or retrospectively on patient demographics including age, sex, height, weight, and ethnicity, as well as the presence of predefined comorbidities. ***General data will be collected from each site using the SPRINT-SARI data tool, namely the WHO and ISARIC NOVEL CORONAVIRUS (nCoV) ACUTE RESPIRATORY INFECTION***

CLINICAL CHARACTERISATION (https://isaric.tghn.org/novel-coronavirus/). As shown in figure 4, SPRINT-SARI data collection will start upon admission to the Hospital. The CRF was assembled by ISARIC members on the basis of the WHO natural history protocol, INFINITE (ANZICS), MOSAIC and others[5,26]. The CRF was assembled to be a basic CRF with the aims of avoiding data duplication, and with the intention of being user friendly and applicable in all settings, regardless of the resources available[27]. The CRF has previously been used in Singapore, New Zealand, Saudi Arabia, Vietnam, and North America and adapted by a working group for the purposes of this study with ISARIC approval to all changes made. In 2020, with the emergence of the COVID-19 epidemics, the ISARIC CRF eCRF were modified in order to characterize patients with this infection. In addition, Chief Investigators of the ECMOCARD trial further improved the ISARIC CRF eCRF to specifically describe COVID-19 patients admitted to the ICU and undergoing mechanical ventilation and ECMO.

COVID-19 Critical Care Consortium observational study Data Collection

Streamlined data-collection instruments and procedures will be used in an attempt to minimise the work in study centres. Specifically, we will collect data on the timing of ICU admission, endotracheal intubation, mechanical ventilation and ECMO commencement in relation to presumed onset of symptoms and hospital admission. We will investigate whether invasive mechanical ventilation and ECMO treatment was commenced in the participating hospital or whether the patient was retrieved and transferred while receiving invasive mechanical ventilation and/or ECMO from a referral centre. Severity of illness before endotracheal intubation and before ECMO will be investigated by respiratory rate, severity of hypoxemia, hypercapnia, non-pulmonary vital organ support, ventilator settings, and use of rescue ARDS therapies in the 12 hours before ECMO commencement. Dynamics of invasive mechanical ventilation and ECMO treatment will be recorded and characterized from commencement of invasive mechanical ventilation up to discontinuation (Figure 4). We will also collect administration of antiviral and antibiotic medications. Finally, duration of mechanical ventilation, ECMO, ICU and hospital stay, ICU and hospital mortality will be documented. In patients who died during hospital admission, we characterized the mode of death from a list of predefined options. Each patient's ELSO Registry patient identification number will be collected so that each patient record may be linked with the data contained within the ELSO Registry, which will be made as a formal data request to ELSO following ELSO procedures to

complement that collected as part of the COVID-19 Critical Care consortium observational study. ***Of note, In Australian centres, patients enrolled into the study "A comprehensive national registry on the treatment and outcomes of patients requiring ECMO) (EXCEL Study) will be identified by the ECMOCARD eCRF. Likewise, in the EXCEL study eCRF, a specific question will be added to identify patients enrolled in the ECMOCARD. Thus, we will complement ECMOCARD CRF with data collected through the EXCEL study.***

Figure 4

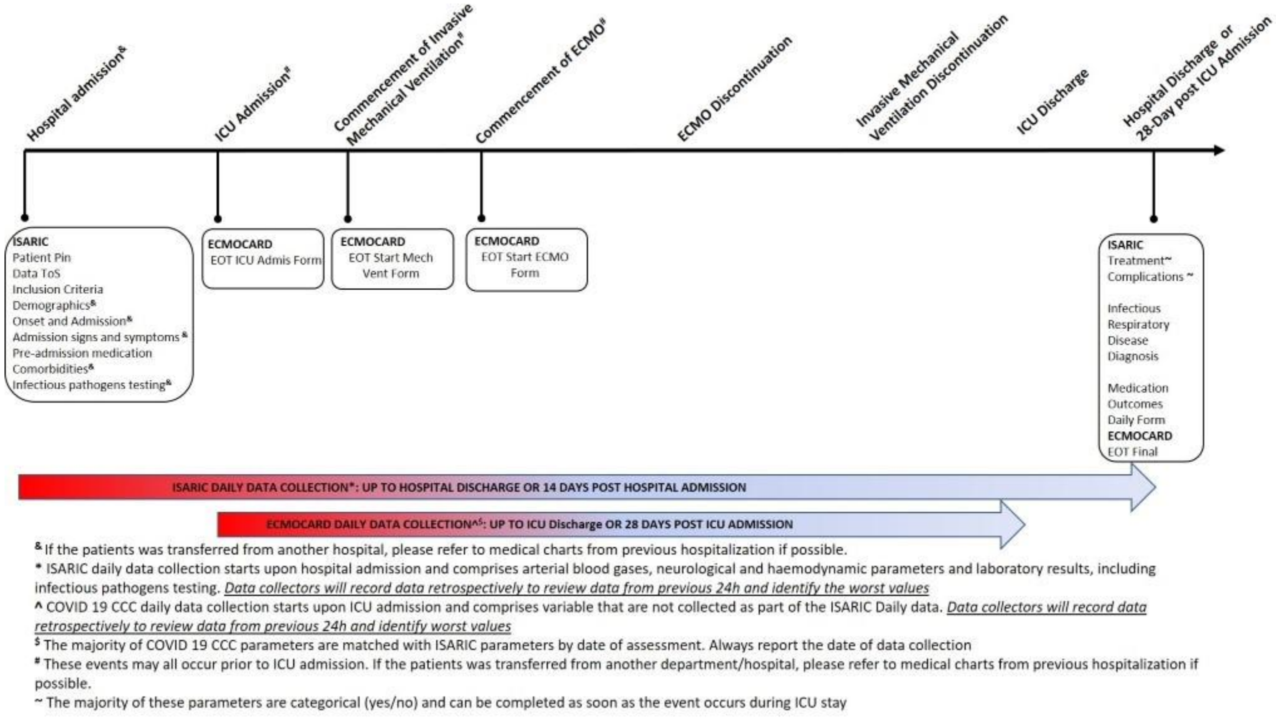

Figure 4 Caption: Follow-up schedule and assessments. ICU, intensive care unit; ECMO, extracorporeal membrane oxygenation.

COVID-19 Critical Care Consortium observational study Basic Data Collection (Optional)

In collaborating sites with limited resources for data collection a modified Basic CRF will be proposed. In particular, we will use a CRF with significant reduction in data collection frequency, while ensuring collection of valuable data to achieve research targets and analysis of clinically relevant outcomes. No new data variables will be collected as part of the Basic CRF, but the frequency of daily data collection will be reduced from 14 days from hospital admission and on the day of ICU admission (ISARIC Daily form on REDCap) and every day of mechanical ventilation

(ECMOCARD EOT Daily form on REDCap), to a maximal total of 7 non-consecutive days as per the following timepoints.

1) Upon hospital admission:
    - Inclusion Criteria form
    - Demographics form
    - Onset and Admission form
    - Admission Signs and Symptoms form
    - Pre-admission medication form
    - Comorbidities form
    - **Daily form**
2) Upon ICU admission
    - **Daily form**
    - EOT ICU Admis form
3) Four days after ICU admission.

    If patient is not mechanically ventilated:
    - **Daily form**

    If patient is mechanically ventilated:
    - **Daily form**
    - **EOT Daily form**
4) Upon commencement of mechanical ventilation:
    - **Daily form**
    - EOT Start Mech Vent form
    - **EOT Daily form**
5) Upon ECMO commencement:
    - **Daily form**
    - EOT Start ECMO form
    - **EOT Daily form**
6) Upon ECMO discontinuation:
    - **Daily form**

- **EOT Daily form**

7) Upon mechanical ventilation discontinuation:
    - **Daily form**
    - **EOT Daily form**

8) Upon hospital discharge or 28 days post ICU admission, whichever occurs later:
    - Treatment form
    - Complications form
    - Infectious Respiratory Disease Diagnosis form
    - Medication form
    - Outcome form
    - EOT Final form

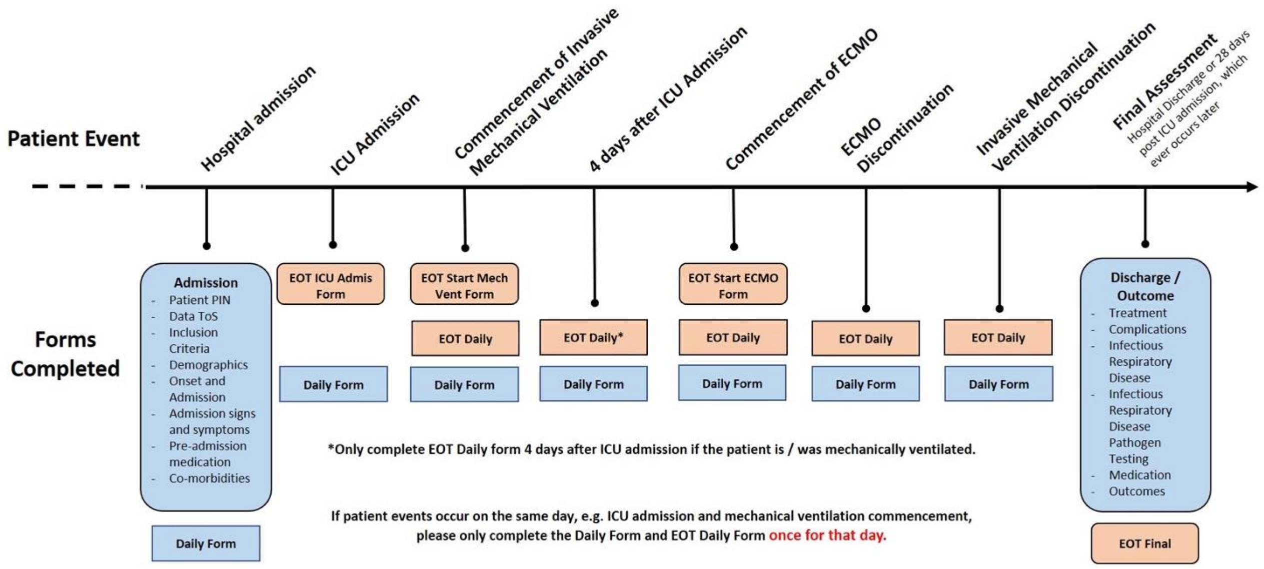

Figure 5 Caption: Basic case report form follow-up schedule and assessments showing the maximal number of assessments. ICU, intensive care unit; ECMO, extracorporeal membrane oxygenation.

Coagulation Disorders and Thrombosis Sub-study Data Collection (Optional)

In collaborative centres that routinely perform rotational thromboelastometry (ROTEM) or thromboelastography (TEG) in their clinical practice, we will carry out an additional observational sub-study to appraise coagulation disorders and/or pro-thrombotic risks in COVID-19 patients in the ICU. As detailed in following paragraphs, upon admission to ICU, and every 24 hours thereafter, we

will collect data prospectively or retrospectively on coagulation disorders and pro-thrombotic risks until discontinuation of mechanical ventilation or in case of patients who are not mechanically ventilated, until 7 days post-ICU discharge. In addition, in centres that routinely use ROTEM, within 1h from a clinically relevant thrombosis/embolism or bleeding event, and 6h prior to commencement of ECMO, we will perform an additional ROTEM assessment to record TRAPTEM AUC, A6 and MS parameters. Following IRB approval of the Covid-19 Critical Care Consortium incorporating ECMOCARD protocol version 1.2.7, ***data for the Coagulation Disorders and Thrombosis Sub-study will be collected from each collaborating site using the dedicated REDcap CRF, hosted at Oxford University***.

Neurology Sub-study Data Collection (Optional)

In collaborative centres with specific interest in the neurological impact of COVID-19, we will carry out an additional observational sub-study. As detailed in following paragraphs, we will collect data retrospectively on neurological comorbidities, central and peripheral nervous system complications during the hospital admission for COVID-19. In addition we will record crucial data on neuroimaging and markers of neurological injury. Finally, major outcomes and neurological function up to 28 days post ICU admission will be recorded. Following IRB approval of the Covid-19 Critical Care Consortium incorporating ECMOCARD protocol version 1.2.8, ***data for the Neurology Sub-study will be collected from each collaborating site using the dedicated REDcap CRF, hosted at Oxford University***.

Cardiac Sub-study Data Collection (Optional)

In collaborative centres with specific interest in the cardiac impact of COVID-19, we will carry out an additional observational sub-study. As detailed in following paragraphs, we will collect data retrospectively on cardiac comorbidities, cardiac complications during the hospital admission for COVID-19, including myocardial infarction, arrhythmias, cardiogenic shock, cardiac arrest and any cardiac support provided. In addition we will record essential echocardiography data and markers of cardiac injury. Major outcomes up to 28 days post ICU admission will be obtained from the main COVID-19 Critical Care Consortium observational study. Following IRB approval of the Covid-19 Critical Care Consortium incorporating ECMOCARD protocol version 1.2.8, ***data for the Cardiac Sub-***

study will be collected from each collaborating site using the dedicated REDcap CRF, hosted at Oxford University.

Acute kidney injury Sub-study Data Collection (Optional)

In collaborative centres with specific interest in the impact of COVID-19 on kidneys function, we will carry out an additional observational sub-study. As detailed in following paragraphs, we will retrospectively collect additional parameters to evaluate:

1. Number of COVID-19 patients developing acute kidney injury (AKI) as defined by AKIN/KDIGO network criteria[28], using creatinine and urine output as a definition
2. Influence of altered coagulation on AKI incidence and on mortality in COVID-19 AHRF/ARDS
3. Effect of MV modalities on AKI, specifically PEEP, proning and neuromuscular blockade
4. Outcomes of AKI in this population, including extent of recovery or renal function.
5. Mortality difference based on stages of AKIN/KDIGO network criteria AKI in patients with COVID-19 AHRF/ARDS

Of note, following IRB approval of the Covid-19 Critical Care Consortium incorporating ECMOCARD protocol version 1.2.8, ***data for the Cardiac Sub-study will be collected from each collaborating site using the dedicated REDcap CRF, hosted at Oxford University***.

Data collection methods

Each site will have the option to collect data via Option 1 alone ***OR*** Option 1 +2. The method chosen will be a decision made at a site level. The options for data collection are as follows:

OPTION 1: Standard Data Collection

Both the SPRINT-SARI ISARIC and ECMOCARD CRF will be made available at all participating sites as a paper CRF. The SPRINT-SARI ISARIC and ECMOCARD CRFs will be available in a variety of languages and will be translated into languages appropriate for all participating sites. The translation of the paper and electronic CRFs from English into the required language will be the responsibility of the national lead investigators and collaborators of the Critical Care Research group and checked for consistency by an appropriate investigator in the relevant country. All data will be collected by trained staff at each study site and these individuals will enter all required data described in the protocol into the CRFs directly from the source data. Trained staff at sites with the

IT capabilities can enter all required data directly into the protected online database, known as the eCRF; paper CRFs are the alternative option for direct data entry with subsequent transcription, upon completion, into the eCRF. Information recorded in the CRF should accurately reflect the participants' medical/hospital notes. The Research Coordinator or Site Investigator will have the ability to choose the process they use to enter data into the eCRF, where data may be entered at one time or intermittently. If used, the original paper based CRF will be stored within a locked office in each study site. The intent of this process is to improve the quality of the clinical study by providing prompt feedback to the Investigators on the progress of the submitted data and to enhance the ability to collect early safety information in a more timely fashion to fully comply with the intent of GCP requirements. ***Data from International countries will be entered into an online eCRF database managed by the Oxford University Clinical Research Unit (OUCRU) for the SPRINT-SARI ISARIC and ECMOCARD tiers. Data from Australia will be entered into an online eCRF database managed by Monash University, and will be complemented with data from*** SPRINT SARI observational study (ALFRED HREC Reference 202/16) and EXCEL (ALFRED HREC Reference 534/18))***.*** In countries unable to upload data on a centralised database the right to retain a local database on a national server is available with aggregated completely anonymised data exported centrally for analysis. Each site will be identified via a 3-digit network code, a 3-digit site code, and each patient will be assigned a 4-digit sequential patient code making up the patient ID number at time of originally enrolment in SPRINT- SARI. The site-code will be specified as to whether it is an ICU, hospital ward, or other facility. The site code is obtained by registering on the eCRF, data management system. Patient numbers should be assigned sequentially for each site beginning with 0001. In the case of a single site recruiting patients on different wards, or where it is otherwise difficult to assign sequential numbers, it is acceptable to assign numbers in blocks. Alpha characters can also be used (e.g. Intensive Care Unit will assign A001 onwards, in-patient ward will assign B001 onwards). ***The full patient identification number will therefore be a 10-digit number, with the format of the following: network code - site code – individual patient code [_][_][_]-[_][_][_]-[_][_][_][_](eg. 001-012-0001). The register of patient names and study numbers will not leave the participating hospital. Access to the data entry system will be protected by username and password.*** Username and password will be assigned during the registration process for individual

Research Coordinators or Site Investigators. All electronic data transfer between study site and database will be username and password protected. Each centre will maintain a trial file including a protocol, ethics approval documentation, and paper CRFs. A participant list will be used in each study site to match identifier codes in the database to individual patients in order to record clinical outcomes and supply any missing data points. The Participant List is maintained locally and is not to be transferred to any other location. The Research Coordinator will compile an enrolment log including the patient's name, age, hospital identification number and unique study number. Subsequent data will be identified by the unique study number only. The enrolment log and study data will be kept separately.

OPTION 2: Interactive augmented data collection

We will use platforms and solutions provided by Amazon to collect data and transfer data into the REDcap web application. Data will be collected through 1) voice commands; 2) digital video monitor interface and 3) through digital transcription of parameters collected via SPRINT-SARI/ECMOCARD paper CRFs. Similar to option 1, only de-identified information will be collected, encrypted and transferred directly to the REDCAP database. No data or information of any kind will be directed elsewhere. Amazon Web Services will not have any direct interaction with the enhanced user-interface once it is implemented and will only act in an external consultancy capacity. Data will be fully encrypted from data ingestion into Amazon cloud, up to de-encryption into the REDcap web application. Thus Amazon platform will only channel, without being able to codify, data from hospitals into the REDcap system.

Data collection methods (Coagulation Disorders and Thrombosis sub-study)

As for the Coagulation Disorders and Thrombosis Sub-study, the CRF will be also made available at all collaborating sites as a paper CRF. The Coagulation Disorders and Thrombosis Sub-study CRF will be only available in English. Data will be collected by trained staff at each study site and these individuals will enter all required data described in the protocol into the CRFs directly from laboratory results, ROTEM or TEG reports. All data will only be collected if done as part of standard clinical practice. If clinical tests are not routinely completed at the study time points, this data is not required. Trained staff at sites with the IT capabilities can enter all required data directly

into the protected online database hosted at Oxford University, known as the eCRF; paper CRFs are the alternative option for direct data entry with subsequent transcription, upon completion, into the eCRF. Information recorded in the CRF should accurately reflect the participants' laboratory results, ROTEM or TEG reports. The Research Coordinator or Site Investigator will have the ability to choose the process they use to enter data into the eCRF, where data may be entered at one time or intermittently. If used, the original paper based CRF will be stored within a locked office in each study site. The intent of this process is to improve the quality of the clinical study by providing prompt feedback to the Investigators on the progress of the submitted data and to enhance the ability to collect early safety information in a more timely fashion to fully comply with the intent of GCP requirements. ***Data will be entered into an online eCRF database managed by Oxford University.*** In Countries unable to upload data on a centralised database the right to retain a local database on a national server is available with aggregated completely anonymised data exported centrally for analysis. ***The full patient SPRINT-SARI/ECMOCARD identification number will be recorded to match results of the Coagulation Disorders and Thrombosis Sub-study with SPRINT-SARI/ECMOCARD records. The register of patient names and study numbers will not leave the participating hospital. Access to the data entry system will be protected by username and password.*** Username and password will be assigned by Oxford University, during the registration process for individual Research Coordinators or Site Investigators. All electronic data transfer between study site and database will be username and password protected. The Participant List of the Coagulation Disorders and Thrombosis Sub-study is maintained locally and is not to be transferred to any other location.

Data collection methods (Neurology sub-study)

As for the Neurology Sub-study, the CRF will be also made available at all collaborating sites as a paper CRF. The Neurology Sub-study CRF will be only available in English. Data will be collected by trained staff at each study site and these individuals will enter all required data described in the protocol into the CRFs directly from medical charts, laboratory results and medical imaging reports. All data will only be collected if done as part of standard clinical practice. If clinical tests are not routinely completed at the study time points, this data is not required. Trained staff at sites with the IT capabilities can enter all required data directly into the protected online database hosted at

Oxford University, known as the eCRF; paper CRFs are the alternative option for direct data entry with subsequent transcription, upon completion, into the eCRF. Information recorded in the CRF should accurately reflect the participants' details recorded in medical charts, laboratory results and medical imaging reports. The Research Coordinator or Site Investigator will have the ability to choose the process they use to enter data into the eCRF, where data may be entered at one time or intermittently. If used, the original paper based CRF will be stored within a locked office in each study site. The intent of this process is to improve the quality of the clinical study by providing prompt feedback to the Investigators on the progress of the submitted data and to enhance the ability to collect early safety information in a more timely fashion to fully comply with the intent of GCP requirements. ***Data will be entered into an online eCRF database managed by the Oxford University.*** In Countries unable to upload data on a centralised database the right to retain a local database on a national server is available with aggregated completely anonymised data exported centrally for analysis. ***The full patient SPRINT-SARI/ECMOCARD identification number will be recorded to match results of the Neurology Sub-study with SPRINT-SARI/ECMOCARD records. The register of patient names and study numbers will not leave the participating hospital. Access to the data entry system will be protected by username and password.*** Username and password will be assigned by the Oxford University during the registration process for individual Research Coordinators or Site Investigators. All electronic data transfer between study site and database will be username and password protected. The Participant List of the Neurology Sub-study is maintained locally and is not to be transferred to any other location.

Data collection methods (Cardiac sub-study)

As for the Cardiac Sub-study, the CRF will be also made available at all collaborating sites as a paper CRF. The Cardiac Sub-study CRF will be only available in English. Data will be collected by trained staff at each study site and these individuals will enter all required data described in the protocol into the CRFs directly from medical charts, laboratory results, and medical imaging reports. All data will only be collected if done as part of standard clinical practice. If clinical tests are not routinely completed at the study time points, this data is not required. Trained staff at sites with the IT capabilities can enter all required data directly into the protected online database hosted at Oxford University, known as the eCRF; paper CRFs are the alternative option for direct data entry

with subsequent transcription, upon completion, into the eCRF. Information recorded in the CRF should accurately reflect the participants' details recorded in medical charts, laboratory results, and medical imaging reports. The Research Coordinator or Site Investigator will have the ability to choose the process they use to enter data into the eCRF, where data may be entered at one time or intermittently. If used, the original paper based CRF will be stored within a locked office in each study site. The intent of this process is to improve the quality of the clinical study by providing prompt feedback to the Investigators on the progress of the submitted data and to enhance the ability to collect early safety information in a more timely fashion to fully comply with the intent of GCP requirements. ***Data will be entered into an online eCRF database managed by Oxford University.*** In Countries unable to upload data on a centralised database the right to retain a local database on a national server is available with aggregated completely anonymised data exported centrally for analysis. ***The full patient SPRINT-SARI/ECMOCARD identification number will be recorded to match results of the Neurology Sub-study with SPRINT-SARI/ECMOCARD records. The register of patient names and study numbers will not leave the participating hospital. Access to the data entry system will be protected by username and password.*** Username and password will be assigned by Oxford University during the registration process for individual Research Coordinators or Site Investigators. All electronic data transfer between study site and database will be username and password protected. The Participant List of the Cardiac Sub-study is maintained locally and is not to be transferred to any other location.

Data collection methods (Acute Kidney Injury sub-study)

As for the Acute Kidney Injury Sub-study, the CRF will be also made available at all collaborating sites as a paper CRF. The Acute Kidney Injury Sub-study CRF will be only available in English. Data will be collected by trained staff at each study site and these individuals will enter all required data described in the protocol into the CRFs directly from medical charts, laboratory results, and medical imaging reports. All data will only be collected if done as part of standard clinical practice. If clinical tests are not routinely completed at the study time points, this data is not required. Trained staff at sites with the IT capabilities can enter all required data directly into the protected online database hosted at Oxford University, known as the eCRF; paper CRFs are the alternative option for direct data entry with subsequent transcription, upon completion, into the

eCRF. Information recorded in the CRF should accurately reflect the participants' details recorded in medical charts, laboratory results, and medical imaging reports. The Research Coordinator or Site Investigator will have the ability to choose the process they use to enter data into the eCRF, where data may be entered at one time or intermittently. If used, the original paper based CRF will be stored within a locked office in each study site. The intent of this process is to improve the quality of the clinical study by providing prompt feedback to the Investigators on the progress of the submitted data and to enhance the ability to collect early safety information in a more timely fashion to fully comply with the intent of GCP requirements. ***Data will be entered into an online eCRF database managed by Oxford University.*** In Countries unable to upload data on a centralised database the right to retain a local database on a national server is available with aggregated completely anonymised data exported centrally for analysis. ***The full patient SPRINT-SARI/ECMOCARD identification number will be recorded to match results of the Neurology Sub-study with SPRINT-SARI/ECMOCARD records. The register of patient names and study numbers will not leave the participating hospital. Access to the data entry system will be protected by username and password.*** Username and password will be assigned by Oxford University during the registration process for individual Research Coordinators or Site Investigators. All electronic data transfer between study site and database will be username and password protected. The Participant List of the Acute Kidney Injury Sub-study is maintained locally and is not to be transferred to any other location.

Screening log

No screening log will be maintained.

Data quality

Several procedures to ensure data quality and protocol standardisation will help to minimise bias. These include:

1. Online meetings for all research coordinators will be held to ensure consistency in procedures;
2. A detailed data dictionary will define the data to be collected on the case report form;

3. Quality checks will be built into the data management system and there will be quality checks of critical data points entered into the CRFs to ensure standardization and validity of the data collected;

An achievable data set will be fundamental to the success of the study. We have identified the key data points whilst not discouraging centres from participating through an excessive burden of data collection. Data queries may be generated, depending on resource availability. Any information that is not available for the investigator will not be considered as missing. No assumptions will be made for missing data.

Data management

Data entry and data management will be coordinated by ISARIC and ECMOCARD steering committee, including programming and data management support. On behalf of the management committee, ANZIC-RC and ISARIC will act as custodian of the data. The University of Queensland will receive data from the data custodians via data sharing agreements. The management committee of the trial will take responsibility for the content and integrity of any data. There will be periodic assessments of data burden to ensure that the infrastructure is organized to handle large amounts of incoming data in small time periods. SPRINT-SARI and ECMOCARD will adhere to the research and data sharing policies of ISARIC, Sample and Data Sharing Policy, Version 4, 21 July 2014. ***Clinical investigators contributing to the research efforts will be given full recognition for their efforts and will be given the opportunity to access data. Ownership of any data transferred to the eCRF will be retained by the site that contributed it.*** Networks will retain the right to request raw data for all sites included in their network for research purposes, provided that the research proposal has been reviewed and approved by the management committee, ISARIC and ECMOCARD following publication of the primary manuscript. All analysis of pooled data will be undertaken with the explicit agreement of each site who contributed. ISARIC and ECMOCARD will retain the right to use all pooled data for scientific and other purposes. All members of the study group will have the right to access the pooled data for research purposes provided the research proposal has been reviewed and deemed satisfactory by the management committee following publication of the primary manuscript. Only summary data will be presented publicly. Individual patient data provided by participating sites will remain the property of the respective institution. Of note, a data

management plan will be developed to address researchers' intentions related to generation, collection, access, use, analysis, disclosure, storage, retention, disposal, sharing and re-use of data and information, the risks associated with these activities and any strategies for minimising those risks.

Monitoring

Data monitoring will be conducted on a randomly selected subset (up to 5%) of cases, through discussion with the local site investigator to discuss data collection techniques. Direct site visits will not be feasible, given the scope of the study.

Collected Parameters

The following parameters will be assessed and recorded based on the follow-up schedule and assessments reported in Figure 4.

Demographics and Medical History

1. Personal Data
2. Medical History and comorbidities, including type of anti-hypertensive medications
3. Smoking habits
4. Chronic alcohol abuse
5. Intravenous drug abuse
6. Immuno-competency status

COVID-19 infection

1. Date of first signs of infection
2. Date of hospital admission
3. Date of ICU admission
4. Date of invasive mechanical ventilation
5. Blood gases before commencement of invasive mechanical ventilation
6. Use of continuous renal replacement therapy before commencement of invasive mechanical ventilation
7. Use of vasoactive drugs before commencement of invasive mechanical ventilation
8. Use of cardiac-assist devices before commencement of invasive mechanical ventilation
9. Acute physiology and chronic health evaluation (APACHE II) score upon ICU admission

10. Use of anti-viral treatment
11. Use of antibiotics
12. Cutaneous manifestations

Clinical parameters upon commencement of invasive mechanical ventilation

1. Date of invasive mechanical ventilation commencement
2. Use of prone position
3. Use of neuromuscular blockade
4. Use of recruitment manoeuvres
5. Use of inhaled nitric oxide
6. Use of bicarbonate
7. Blood gases
8. Ventilatory mode
9. Inspiratory fraction of oxygen
10. Respiratory rate
11. Tidal volume (ml/Kg of ideal body weight)
12. Positive end-expiratory pressure
13. Airway plateau pressure

Daily assessment of clinical parameters during invasive mechanical ventilation

1. Date of assessment
2. Use of prone position
3. Use of neuromuscular blockade
4. Use of recruitment manoeuvres
5. Use of inhaled nitric oxide
6. Blood gases
7. Ventilatory mode
8. Inspiratory fraction of oxygen
9. Respiratory rate
10. Tidal volume (ml/Kg of ideal body weight)
11. Positive end-expiratory pressure

12. Airway plateau pressure
13. Haemoglobin
14. White blood cells
15. AST
16. ALT
17. Lactate
18. Creatinine
19. Ferritin
20. D-dimer
21. Troponins
22. BNP
23. Use of continuous renal replacement therapy
24. Use of vasoactive drugs
25. Use of anticoagulants
26. Transfused blood products
27. Infectious complications
28. Haemorrhagic complications

Clinical features before commencement of ECMO

1. Date of ECMO commencement
2. Use of prone position
3. Use of neuromuscular blockade
4. Use of recruitment manoeuvres
5. Use of inhaled nitric oxide
6. Use of bicarbonate
7. Blood gases
8. Ventilatory mode
9. Inspiratory fraction of oxygen
10. Respiratory rate
11. Tidal volume (ml/Kg of ideal body weight)

12. Positive end-expiratory pressure
13. Airway plateau pressure

ECMO characteristics

1. Type and manufacturer of centrifugal blood pump driven circuit
2. Type and manufacturer of low-resistance oxygenator
3. Type of ECMO: venous-venous or venous-arterial
4. Peripheral access: femoral, jugular, both
5. ECMO blood flow rate day 0, and every 24 hours thereafter
6. ECMO gas flow rate day 0, and every 24 hours thereafter
7. Anticoagulation during ECMO
8. Frequency of ECMO circuit change
9. Ventilatory settings on ECMO
10. Vasoactive support on ECMO
11. Organ dysfunctions on ECMO

ECMO adverse effects

1. Transfused blood during ECMO
2. Transfused plasma during ECMO
3. Transfused platelets during ECMO
4. Transfused cryoprecipitates during ECMO
5. Type and source of infectious complications
6. Type and source of haemorrhagic complications
7. Other complications

Daily assessments for Coagulation Disorders and Thrombosis Sub-study

1. SPRINT-SARI/ECMOCARD patient number
2. Date of assessment
3. Lactate dehydrogenase
4. Ferritin
5. D-dimer
6. Fibrinogen

7. Activated clotting time
8. Activated partial thromboplastin time
9. International normalised ration
10. Plasma free haemoglobin
11. ROTEM parameters (EXTEM, FIBTEM, INTEM, HEPTEM, TRAPTEM, NATEM if patients undergoing treatment with low molecular weight heparin and ECATEM if patients undergoing treatment with direct thrombin inhibitors)
12. TEG parameters

Main outcomes

1. Date of ECMO discontinuation
2. Date of invasive mechanical ventilation discontinuation
3. Date of ICU Discharge
4. Date of Hospital Discharge
5. Mortality at 28 days
6. Main cause of death

Sub-studies

As mentioned above, site investigators will have the option to collect additional clinical data on the impact of COVID-19 on coagulation disorders and thrombosis, neurology, cardiac and acute kidney injury as part of specific sub-studies focusing on these clinical features. The following parameters per each sub-study will be assessed and recorded.

Coagulation disorders and thrombosis sub-study

Investigators interested in coagulation disorders and thrombosis sub-study will collect daily following parameters, if available as part of standard clinical practice:

1. Laboratory parameters (PT aPTT INR ACT LDH; Fibrinogen; Plasma Free Hemoglobin Anti-Xa; Ferritin; D-Dimer; IL-6; CRP; Lupus Anticoagulant Protein C; Von Willebrand Factor Antigen; Antithrombin; Ristocetin)
2. Rotem or TEG parameters

3. Medications and dosing (Heparin; Heparin infusion (IV); Low Molecular weight heparin; Warfarin; Rivaroxaban; Apixaban; Aspirin; Argatroban; Bivalrudin; DDAVP; AMICAR (epsilon-Aminocaproic acid); Tranexamic Acid; Protamine; Andexanet Alfa)
4. Bleeding and thrombosis events
5. Administered blood products

Neurology sub-study

Investigators interested in the neurology sub-study will collect following parameters, if available as part of standard clinical practice:

1. Previous chronic neurological disorders
2. Modified Rankin scale
3. Central nervous system complications during ICU stay (ischemic stroke; intracranial haemorrhage; hypoxic ischemic brain injury; meningitis/encephalitis; transverse myelitis; seizure; delirium)
4. Peripheral nervous system complications during ICU stay (Guillan-Barre syndrome; critical illness myopathy-neuropathy; hypogeusia/hyposmia)
5. Management of above-mentioned complications
6. Results of neuro-imaging assessments
7. Biomarkers
8. Withdrawal of treatment and modified RANKIN scale at ICU discharge and 28 days thereafter

Cardiac sub-study

Investigators interested in the cardiology sub-study will collect following parameters, if available as part of standard clinical practice:

1. Previous chronic cardiac disorders (ischemic heart disease; angina; heart failure; arrhythmias; permanent pacemaker/implanted cardia defibrillator/previous cardiac resynchronization therapy; heart transplant; mechanical circulatory support device; congenital heart disease; cardiomyopathy; previous cardiac arrest)
2. Cardiac complications during ICU stay and management during and post event (acute myocardial infarction; myocarditis; Takotsubo cardiomyopathy; new onset arrythmias; cardiac arrest)

3. Medical therapy of shock state
4. Mechanical circulatory support
5. Results of echocardiography
6. Biomarkers
7. Administered blood products

Acute kidney injury sub-study

In patients in whom mild acute kidney injury develops (serum creatinine rise >20% from baseline; or upper normal level where no evidence of chronic renal failure) Investigators interested in the acute kidney injury sub-study will collect, the following parameters, if available as part of standard clinical practice:

1. *Upon ICU admission*: Baseline renal function at or prior hospital admission (serum creatinine; urine specific gravity; proteinuria; haematuria) and medications prior to ICU admission (NSAIDS; Aminoglycoside; Vancomycin; Diuretics; ACEI/ARBs)
2. *Daily:* Medications (NSAIDS; Aminoglycoside; Vancomycin; Diuretics; ACEI/ARBs); laboratory and clinical parameters (fluid and drug volume infused in last 24hrs; carboxyhaemoglobin); dialysis features (main indication; type; anticoagulation; calcium; complications)
3. *Final outcomes:* Dialysis-dependent status at ICU and hospital discharge

Data Analysis

The global analysis of SPRINT-SARI/ECMOCARD and Coagulation Disorders and Thrombosis Sub-study categorical variables will be described as proportions and will be compared using chi-square or Fisher's exact test. Continuous variables will be described as mean and standard deviation if normally distributed or median and inter-quartile range if not normally distributed. Comparisons of continuous variables will be performed using one-way ANOVA or Mann-Whitney test, as appropriate. A logistic regression model will be performed to assess independent association between prognostic factors and outcomes, taking into account the hierarchical nature of the data. Significance will be set at $p<0.05$.

Reference List

1. Bolotin S, Pebody R, White PJ, et al. A new sentinel surveillance system for severe influenza in England shows a shift in age distribution of hospitalised cases in the post-pandemic period. *PLoS One*. 2012;7(1). doi:10.1371/journal.pone.0030279
2. Dawood FS, Iuliano AD, Reed C, et al. Estimated global mortality associated with the first 12 months of 2009 pandemic influenza A H1N1 virus circulation: A modelling study. *Lancet Infect Dis*. 2012;12(9):687-695. doi:10.1016/S1473-3099(12)70121-4
3. Simonsen L, Spreeuwenberg P, Lustig R, et al. Global Mortality Estimates for the 2009 Influenza Pandemic from the GLaMOR Project: A Modeling Study. *PLoS Med*. 2013;10(11). doi:10.1371/journal.pmed.1001558
4. Huang QS, Baker M, McArthur C, et al. Implementing hospital-based surveillance for severe acute respiratory infections caused by influenza and other respiratory pathogens in New Zealand. *West Pacific Surveill response J WPSAR*. 2014;5(2):23-30. doi:10.5365/WPSAR.2014.5.1.004
5. Critical Care Services and 2009 H1N1 Influenza in Australia and New Zealand. *N Engl J Med*. 2009;361(20):1925-1934. doi:10.1056/NEJMoa0908481
6. Guery B, Poissy J, El Mansouf L, et al. Clinical features and viral diagnosis of two cases of infection with Middle East Respiratory Syndrome coronavirus: A report of nosocomial transmission. *Lancet*. 2013;381(9885):2265-2272. doi:10.1016/S0140-6736(13)60982-4
7. Weiss SR, Navas-Martin S. Coronavirus Pathogenesis and the Emerging Pathogen Severe Acute Respiratory Syndrome Coronavirus. *Microbiol Mol Biol Rev*. 2005;69(4):635-664. doi:10.1128/mmbr.69.4.635-664.2005
8. Drosten C, Günther S, Preiser W, et al. Identification of a Novel Coronavirus in Patients with Severe Acute Respiratory Syndrome. *N Engl J Med*. 2003;348(20):1967-1976. doi:10.1056/NEJMoa030747
9. Ksiazek TG, Erdman D, Goldsmith CS, et al. A Novel Coronavirus Associated with Severe Acute Respiratory Syndrome. *N Engl J Med*. 2003;348(20):1953-1966. doi:10.1056/NEJMoa030781
10. Zaki AM, Van Boheemen S, Bestebroer TM, Osterhaus ADME, Fouchier RAM. Isolation of a

novel coronavirus from a man with pneumonia in Saudi Arabia. *N Engl J Med*. 2012;367(19):1814-1820. doi:10.1056/NEJMoa1211721

11. de Groot RJ, Baker SC, Baric RS, et al. Middle East Respiratory Syndrome Coronavirus (MERS-CoV): Announcement of the Coronavirus Study Group. *J Virol*. 2013;87(14):7790-7792. doi:10.1128/jvi.01244-13
12. WHO | Summary of probable SARS cases with onset of illness from 1 November 2002 to 31 July 2003. *WHO*. 2015.
13. WHO | Middle East respiratory syndrome coronavirus (MERS-CoV). *WHO*. 2020.
14. Chan JF-W, Yuan S, Kok K-H, et al. A familial cluster of pneumonia associated with the 2019 novel coronavirus indicating person-to-person transmission: a study of a family cluster. *Lancet*. January 2020. doi:10.1016/S0140-6736(20)30154-9
15. Zhu N, Zhang D, Wang W, et al. A Novel Coronavirus from Patients with Pneumonia in China, 2019. *N Engl J Med*. January 2020:NEJMoa2001017. doi:10.1056/NEJMoa2001017
16. WHO | Novel Coronavirus – Thailand (ex-China). *WHO*. 2020.
17. WHO | Novel Coronavirus – Japan (ex-China). *WHO*. 2020.
18. WHO | Novel Coronavirus – Republic of Korea (ex-China). *WHO*. 2020.
19. Spina S, Marrazzo F, Migliari M, Stucchi R, Sforza A, Fumagalli R. The response of Milan's Emergency Medical System to the COVID-19 outbreak in Italy. *Lancet (London, England)*. 2020;0(0). doi:10.1016/S0140-6736(20)30493-1
20. Ebrahim SH, Memish ZA. COVID-19: preparing for superspreader potential among Umrah pilgrims to Saudi Arabia. *Lancet*. 2020;0(0). doi:10.1016/S0140-6736(20)30466-9
21. Holshue ML, DeBolt C, Lindquist S, et al. First Case of 2019 Novel Coronavirus in the United States. *N Engl J Med*. 2020;382(10):929-936. http://www.nejm.org/doi/10.1056/NEJMoa2001191. Accessed March 10, 2020.
22. Sun K, Chen J, Viboud C. Early epidemiological analysis of the coronavirus disease 2019 outbreak based on crowdsourced data: a population-level observational study. *Lancet Digit Heal*. 2020;0(0). doi:10.1016/S2589-7500(20)30026-1
23. Huang C, Wang Y, Li X, et al. Clinical features of patients infected with 2019 novel coronavirus in Wuhan, China. *Lancet*. 2020;395(10223):497-506. doi:10.1016/S0140-

6736(20)30183-5

24. Shi H, Han X, Jiang N, et al. Radiological findings from 81 patients with COVID-19 pneumonia in Wuhan, China: a descriptive study. *Lancet Infect Dis*. 2020;0(0). doi:10.1016/S1473-3099(20)30086-4
25. Wang D, Hu B, Hu C, et al. Clinical Characteristics of 138 Hospitalized Patients With 2019 Novel Coronavirus-Infected Pneumonia in Wuhan, China. *JAMA*. February 2020. doi:10.1001/jama.2020.1585
26. André T, Boni C, Mounedji-Boudiaf L, et al. Oxaliplatin, fluorouracil, and leucovorin as adjuvant treatment for colon cancer. *N Engl J Med*. 2004;350(23):2343-2351. doi:10.1056/NEJMoa032709
27. Dunning JW, Merson L, Rohde GGU, et al. Open source clinical science for emerging infections. *Lancet Infect Dis*. 2014;14(1):8-9. doi:10.1016/S1473-3099(13)70327-X
28. Chawla LS, Bellomo R, Bihorac A, et al. Acute kidney disease and renal recovery: Consensus report of the Acute Disease Quality Initiative (ADQI) 16 Workgroup. *Nat Rev Nephrol*. 2017;13(4):241-257. doi:10.1038/nrneph.2017.2

Regulation, Ethics and Governance

Protocol and any following amendment to the original protocol will be translated to the main language of the collaborative institution and submitted for the approval of each institutional review board (IRB). All protocols of the study will require approval by each institutional review board, before enrolment of patients. Sites should apply for a waiver of consent to be granted given the negligible risk nature of the study and the need for rapid data collection to inform pandemic responses globally. Sites wishing to participate in each sub-study will be required to provide the Covid-19 Critical Care Consortium Research Coordinator with an IRB approval certificate. In particular IRB approval of protocol version 1.2.7 will be required to participate to the Coagulation Disorders and Thrombosis study; while, IRB approval of protocol version 1.2.8 will be required to participate to Neurology, Cardiac or Acute Kidney Injury Sub-studies. Only after IRB approval certificate will be provided, sites will be granted access to the relevant sub-study REDCap databases.

Conflict of interest

The investigators of the APELSO network DO NOT have any significant financial or personal interest that would reasonably appear to be affected by the proposed research activities.

Data collection and Site Monitoring plan

Data Collection

Data will be collected in dedicated electronic forms and/or hard copies as provided by the SPRINT-SARI and ISARIC Organisations (APPENDIX B) and the ECMOCARD Steering Committee (APPENDIX C). A custom-designed electronic case report form has been developed in REDcap, which is hosted at the University of Oxford and for all Australian centres will be hosted at Monash University, Melbourne, Australia. A custom-designed electronic case report form has been developed in REDcap for the Coagulation Disorders and Thrombosis Sub-study, which is hosted at the University of Queensland. Hard copies and electronic data will be kept for at least 7 years following the conclusion of the study. Each investigator will be responsible to collect and preserve data obtained at his/her collaborative institution.

Site Monitoring

Periodic conference calls will be organized with all investigators or investigators of specific collaborative centres to monitor the quality of the data collected, address specific issues in data

collection and prepare future publications. Data queries will be generated by the Consortium Data Management Team and disseminated to sites for review and correction in the REDCap database as appropriate.

Compensations

No compensation will be offered to collaborating institutions.

Data Access

All essential documentation of the SPRINT-SARI/ECMOCARD and the Coagulation Disorders and Thrombosis Sub-study will be stored in an Investigator Study File (ISF), which will be held by the Critical Care Research Group (CCRG), University of Queensland. On completion of the study, this information will be archived by the CCRG. Following the publication of the primary and secondary outcomes, additional analyses could be undergone on the data collected. In the event of publications arising from these analyses, those responsible will need to provide the Chief Investigator with a copy of the manuscript for approval prior to submission.

Feasibility

This is a multi-centre study performed within the COVID-19 Critical Care Consortium, which comprises the SPRINT-SARI, ISARIC, ELSO and APELSO networks of clinical research institutions, during an emergent new respiratory infection caused by the new COVID-19 virus. The study will be conducted in intensive care units with broad experience in mechanical ventilation, ECMO and coagulation disorders and thrombosis. Further intra-mural and extra-mural collaborations beyond the COVID-19 Critical Care Consortium and SPRINT-SARI, ISARIC and APELSO networks will be potentially pursued to promptly achieve goals. In summary, the COVID-19 Critical Care Consortium multidisciplinary and international research team of collaborators provides ideal conditions to perform reported study.

Dissemination and Publication

Publication policy

Ownership of the data arising from the study resides with the study teams. Data requested from SPRINT-SARI and EXCEL investigators will resides with their own study teams. After the study, results will be analysed and tabulated, and a study report will be prepared. This report will be made

available to the study collaborators and the relevant IRBs. The study findings will be presented at national and international meetings. We plan to publish our study findings in a high-quality peer reviewed journal. SPRINT-SARI and EXCEL studies will be fully acknowledged in all publications and presentations.

Authorship policy

Authorship will be determined according to the internationally agreed criteria for authorship (www.icmje.org). Authorship of parallel studies conducted outside of the main trial will be according to the individuals involved in the study but must acknowledge the contribution of the involved investigators.

Document S2. A list of recruiting sites and all contributors/collaborators

A list of recruiting sites and all contributors and collaborators

Contributors

Prefix/First Name/Last Name	Site Name
Dr Tala Al-Dabbous<br>Dr Huda Alfoudri<br>Dr Mohammed Shamsah	Al Adan Hospital
Dr Subbarao Elapavaluru<br>Ashley Berg<br>Christina Horn	Allegheny General Hospital
Dr Stephan Schroll	Barmherzige Bruder Regansburg
Dr Jorge Velazco<br>Wanda Fikes<br>Ludmyla Ploskanych	Baylor Scott & White Health - Temple
Dr Dan Meyer<br>Maysoon Shalabi-McGuire<br>Trent Witt<br>Ashley Ehlers	Baylor University Medical Centre, Dallas
Dr Lorenzo Grazioli	Bergamo Hospital
Dr E. Wilson Grandin<br>Jose Nunez<br>Tiago Reyes	Beth Israel Deaconess Medical Centre
Dr Diarmuid O'Briain<br>Stephanie Hunter	Box Hill Hospital
Dr Mahesh Ramanan<br>Julia Affleck	Caboolture Hospital
Dr Sumeet Rai<br>Josie Russell-Brown<br>Mary Nourse	Canberra Hospital
Dr Mark Joseph	Carilion Clinic

Dr Brook Mitchell<br>Martha Tenzer	
Dr Ryuzo Abe	Chiba University Graduate School of Medicine
Dr Hwa Jin Cho<br>Dr In Seok Jeong	Chonnam National University Hospital
Dr Nicolas Brozzi	Cleveland Clinic - Florida
Omar Mehkri	Cleveland Clinic - Ohio
Dr Jerónimo Graf	Clinica Alemana De Santiago
Dr Roderigo Diaz<br>Camila Delgado<br>Joyce González<br>Maria Soledad Sanchez	Clinica Las Condez
Dr Diego Fernando Bautista Rincón<br>Melissa Bustamante Duque<br>Dr Angela Maria Marulanda Yanten	Clinica Valle de Lilli
Dr Daniel Brodie	Medical ICU, Columbia College of Physicians and Surgeons, New-York-Presbyterian Hospital, NY, NY, USA
Dr Desy Rusmawatiningtyas	Dr Sardjito Hospital (Paediatrics)
Gabrielle Ragazzo	Emory University Healthcare System
Dr Azhari Taufik<br>Dr Margaretha Gunawan<br>Dr Vera Irawany<br>Muhammad Rayhan<br>Dr Elizabeth Yasmin Wardoyo<br>Dr Nurindah S Trisnaningrum	Fatmawati Hospital
Dr Mauro Panigada<br>Dr Silvia Coppola<br>Dr Sebastiano Colombo<br>Dr Giacomo Grasselli<br>Dr Michela Leone<br>Dr Alberto Zanella	Fondazione IRCCS Policlinico of Milan (Fondazione IRCCS Ca' Granda Ospedale Maggiore Policlinico)
Prof Massimo Antonelli<br>Dr Simone Carelli	Fondazione Policlinico Universitario Agostino Gemelli IRCCS

Domenico L. Grieco	
Motohiro Asaki	Fujieda Municipal General Hospital
Dr Kota Hoshino	Fukuoka University
Dr Leonardo Salazar<br>Laura Duarte	Fundación Cardiovascular de Colombia
Prof John Laffey<br>Dr Bairbre McNicholas<br>Dr David Cosgrave	Galway University Hospitals
Dr Joseph McCaffrey<br>Allison Bone	Geelong Hospital
Dr James Winearls<br>Mandy Tallott	Gold Coast University Hospital
Dr David Thomson<br>Dr Christel Arnold-Day<br>Jerome Cupido<br>Zainap Fanie<br>Dr Malcom Miller<br>Dr Lisa Seymore<br>Dawid van Straaten	Groote Schuur Hospital
Dr Ibrahim Hassan<br>Dr Ali Ait Hssain<br>Jeffrey Aliudin<br>Al-Reem Alqahtani<br>Khoulod Mohamed<br>Ahmed Mohamed<br>Darwin Tan<br>Joy Villanueva<br>Ahmed Zaqout	Hamad General Hospital - Weill Cornell Medical College in Qatar
Dr Ethan Kurtzman<br>Arben Ademi<br>Ana Dobrita<br>Khadija El Aoudi	Hartford HealthCare

Juliet Segura	
Dr Gezy Giwangkancana	Hasan Sadikin Hospital (Adult)
Dr Shinichiro Ohshimo	Hiroshima University
Dr Koji Hoshino<br>Saito Hitoshi	Hokkaido University Hospital
Dr Javier Osatnik	Hospital Alemán
Dr Anne Joosten	Hospital Civil Marie Curie
Dr Antoni Torres<br>Ana Motos<br>Dr Minlan Yang	Hospital Clinic, Barcelona
Dr Carlos Luna	Hospital de Clínicas
Francisco Arancibia	Hospital del Tórax
Virginie Williams<br>Alexandre Noel	Hospital du Sacre Coeur (Universite de Montreal)
Dr Nestor Luque	Hospital Emergencia Ate Vitarte
Dr Trieu Huynh Trung<br>Sophie Yacoub	Hospital for Tropical Diseases
Marina Fantini	Hospital Mater Dei
Dr Ruth Noemi Jorge García<br>Dr Enrique Chicote Alvarez	Hospital Nuestra Señora de Gracia
Dr Anna Greti	Hospital Puerta de Hierro
Dr Adrian Ceccato	Hospital Universitari Sagrat Cor
Dr Angel Sanchez	Hospital Universitario Sant Joan d'Alacant
Dr Ana Loza Vazquez	Hospital Universitario Virgen de Valme
Dr Ferran Roche-Campo	Hospital Verge de la Cinta de Tortosa
Dr Divina Tuazon	Houston Methodist Hospital
Marcelo Amato<br>Luciana Cassimiro<br>Flavio Pola<br>Francis Ribeiro<br>Guilherme Fonseca	INCOR (Universidade de São Paulo)

Dr Heidi Dalton<br>Dr Mehul Desai<br>Dr Erik Osborn<br>Hala Deeb	INOVA Fairfax Hospital
Dr Antonio Arcadipane<br>Claudia Bianco<br>Raffaele Cuffaro<br>Gennaro Martucci<br>Giovanna Panarello<br>Giovanna Occhipinti<br>Matteo Rossetti<br>Chiara Vitiello	ISMETT
Dr Sung-Min Cho	Johns Hopkins
Dr Hiroaki Shimizu<br>Dr Naoki Moriyama	Kakogawa Acute Care Medical Center
Dr Jae-Burm Kim	Keimyung University Dong San Hospital
Dr Nobuya Kitamura	Kimitsu Chuo Hospital
Dr Alyaa Elhazmi<br>Dr Abdullah Al-Hudaib	King Faisal Specialist Hospital and Research Center
Dr Johannes Gebauer	Klinikum Passau
Dr Toshiki Yokoyama	Kouritu Tousei Hospital
Dr Abdulrahman Al-Fares<br>Esam Alamad<br>Fatma Alawadhi<br>Kalthoum Alawadi<br>Dr Sarah Buabbas	Al-Amiri and Jaber Al-Ahmed Hospitals, Kuwait Extracorporeal Life Support Program
Dr Hiro Tanaka	Kyoto Medical Centre
Dr Satoru Hashimoto<br>Masaki Yamazaki	Kyoto Prefectural University of Medicine
Tak-Hyuck Oh	Kyung Pook National University Chilgok Hospital
Dr Mark Epler<br>Dr Cathleen Forney	Lancaster General Health

Jared Feister<br>Katherine Grobengieser<br>Louise Kruse<br>Joelle Williamson	
Dr Eric Gnall<br>Dr Mara Caroline<br>Sasha Golden<br>Colleen Karaj<br>Sherry McDermott<br>Lynn Sher<br>Dr Timothy Shapiro<br>Lisa Thome<br>Mark Vanderland<br>Mary Welch	Lankenau Institute of Medical Research (Main Line Health)
Dr Matthew Brain<br>Sarah Mineall	Launceston General Hospital
Prof Luca Brazzi<br>Dr Gabriele Sales	Le Molinette Hospital (Ospedale Molinette Torino)
Dr Tawnya Ogston	Legacy Emanuel Medical Center
Dr Dave Nagpal<br>Karlee Fischer	London Health Sciences Centre
Dr Roberto Lorusso	Maastricht University Medical Centre
Prof Mariano Esperatti	Mar del Plata Medical Foundation Private Community Hospital
Dr Diarmuid O’Briain	Maroondah Hospital
Dr Edmund G. Carton	Mater Misericordiae University Hospital
Ayan Sen<br>Amanda Palacios<br>Deborah Rainey	Mayo Clinic College of Medicine
Cassandra Seefeldt<br>Dr Lucia Durham<br>Dr Octavio Falcucci<br>Amanda Emmrich	Medical College of Wisconsin (Froedtert Hospital)

Jennifer Guy<br>Carling Johns<br>Emily Neumann	
Dr Nina Buchtele<br>Dr Michael Schwameis	Medical University of Vienna
Dr Stephanie-Susanne Stecher<br>Delila Singh<br>Dr Michaela Barnikel<br>Lukas Arenz	Medical Department II, LMU Hospital Munich
Dr Akram Zaaqoq<br>Lan Anh Galloway<br>Caitlin Merley	MedStar Washington Hospital Centre
Prof Alistair Nichol	Monash University
Dr Marc Csete<br>Luisa Quesada<br>Isabela Saba	Mount Sinai Medical Centre
Dr Daisuke Kasugai<br>Hiroaki Hiraiwa<br>Taku Tanaka	Nagoya University Hospital
Dr Eva Marwali<br>Yoel Purnama<br>Dr Santi Rahayu Dewayanti<br>Dr Ardiyan<br>Dr Dafsah Arifa Juzar<br>Dr Debby Siagian	National Cardiovascular Center Harapan Kita, Jakarta, Indonesia
Yih-Sharng Chen	National Taiwan University Hospital
Dr Mark Ogino	Nemours Alfred I duPont Hospital for Children
Dr Indrek Ratsep<br>Lauri Enneveer<br>Kristo Erikson<br>Dr Getter Oigus<br>Andra-Maris Post	North Estonia Medical Centre

Piret Sillaots	
Frank Manetta	Northwell Health
Mamoru Komats	Obihiro-Kosei General Hospital
Dr S. Veena Satyapriya<br>Dr Amar Bhatt<br>Marco Echeverria<br>Juan Fiorda<br>Alicia Gonzalez<br>Dr Nahush A. Mokadam<br>Johnny McKeown<br>Joshua Pasek<br>Haixia Shi<br>Alberto Uribe	Ohio State University Medical Centre
Dr Rita Moreno	Oklahoma Heart Institute
Bishoy Zakhary	Oregon Health and Science University Hospital (OHSU)
Dr Marco Cavana<br>Dr Alberto Cucino	Ospedale di Arco (Trento hospital)
Prof Giuseppe Foti<br>Dr Marco Giani<br>Dr Vincenzo Russotto	Ospedale San Gerardo
Prof Davide Chiumello<br>Valentina Castagna	Ospedale San Paolo
Dr Andrea Dell’Amore	Padua University Hospital (Policlinico of Padova)
Dr Hoi-Ping Shum	Pamela Youde Nethersole Eastern Hospital
Dr Alain Vuysteke	Papworth Hospitals NHS Foundation Trust
Dr Asad Usman<br>Andrew Acker<br>Blake Mergler<br>Nicolas Rizer<br>Federico Sertic<br>Benjamin Smood<br>Alexandra Sperry	Penn Medicine (Hospital of the University of Pennsylvania)

Dr Madhu Subramanian	
Dr Erlina Burhan<br>Dr Navy Lolong<br>Dr Ernita Akmal<br>Prof Menaldi Rasmin<br>Bhat Naivedh<br>Dr Faya Sitompul	Persahabatan General Hospital
Dr Peter Barrett<br>Julia Daugherty<br>Dr David Dean	Piedmont Atlanta Hospital
Dr Antonio Loforte	Policlinico di S. Orsola, Università di Bologna
Dr Irfan Khan<br>Olivia DeSantis<br>Dr Mohammed Abraar Quraishi	Presbyterian Hospital Services, Albuquerque
Dr Dominic So<br>Darshana Kandamby	Princess Margaret Hospital
Dr Jose M. Mandei<br>Hans Natanael	Prof Dr R. D. Kandou General Hospital - Paediatric
Eka YudhaLantang<br>Anastasia Lantang	Prof Dr R. D. Kandou General Hospital - Adult
Surya Oto Wijaya	Prof D Sulianti Saroso Hospital
Anna Jung	Providence Saint John's Health Centre
George Ng<br>Dr Wing Yiu Ng	Queen Elizabeth Hospital, Hong Kong
Dr Pauline Yeung	The University of Hong Kong
Dr Alexis Tabah<br>Megan Ratcliffe<br>Maree Duroux	Redcliffe Hospital
Dr Shingo Adachi<br>Dr Shota Nakao	Rinku general medical center (and Senshu trauma and critical care center)
Dr Pablo Blanco<br>Ana Prieto	Rio Hortega University Hospital

Jesús Sánchez	
Dr Meghan Nicholson	Rochester General Hospital
Dr Warwick Butt<br>Alyssa Serratore<br>Carmel Delzoppo	Royal Children's Hospital
Dr Pierre Janin<br>Elizabeth Yarad	Royal North Shore Hospital
Dr Richard Totaro<br>Jennifer Coles	Royal Prince Alfred Hospital
Robert Balk<br>Samuel Fox<br>James Hays<br>Esha Kapania<br>Pavel Mishin<br>Andy Vissing<br>Garrett Yantosh	Rush University, Chicago
Dr Saptadi Yuliarto<br>Dr Kohar Hari Santoso<br>Dr Susanthy Djajalaksana	Saiful Anwar Malang Hospital (Brawijaya University) (Paediatrics)
Dr Arie Zainul Fatoni	Saiful Anwar Malang Hospital (Brawijaya University) (Adult)
Dr Masahiro Fukuda	Saiseikai Senri Hospital
Prof Keibun Liu	Saiseikai Utsunomiya Hospital
Prof Paolo Pelosi<br>Dr Denise Battaglini	San Martino Hospital
Dr Juan Fernando Masa Jiménez	San Pedro de Alcantara Hospital
Dr Sérgio Gaião	São João Hospital Centre, Porto
Jessica Buchner	Sentara Norfolk General Hospital
Dr Su Hwan Lee	Severance Hospital
Dr Tatsuya Kawasaki	Shizuoka Children's Hospital
Dr Pranya Sakiyalak<br>Prompak Nitayavardhana	Siriraj Hospital

Dr Tamara Seitz	Sozialmedizinisches Zentrum Süd - Kaiser-Franz-Josef-Spital
Rakesh Arora<br>David Kent	St Boniface Hospital (University of Mannitoba)
Dr Swapnil Parwar<br>Andrew Cheng<br>Jennene Miller	St George Hospital
Dr Shigeki Fujitani<br>Dr Naoki Shimizu	St Marianna Medical University hospital
Dr Jai Madhok<br>Dr Clark Owyang	Stanford University Hospital
Dr Hergen Buscher<br>Claire Reynolds	St Vincent's Hospital
Dr Olavi Maasikas<br>Dr Aleksandr Beljantsev<br>Vladislav Mihnovits	Tartu University Hospital
Dr Takako Akimoto<br>Mariko Aizawa<br>Dr Kanako Horibe<br>Ryota Onodera	Teine Keijinkai Hospital
Prof Carol Hodgson<br>Dr Aidan Burrell<br>Meredith Young	The Alfred Hospital
Timothy Smith	The Christ Hospital
Dr Timothy George	The Heart Hospital Baylor Plano, Plano
Dr Kiran Shekar<br>Niki McGuinness<br>Lacey Irvine	The Prince Charles Hospital
Brigid Flynn	The University of Kansas Medical Centre
Dr Keiki Shimizu	Tokyo Metropolitan Medical Center
Dr Eddy Fan<br>Kathleen Exconde	Toronto General Hospital
Dr Shingo Ichiba	Tokyo Women's Medical University Hospital

Leslie Lussier	Tufts Medical Centre (and Floating Hospital for Children)
Dr Gösta Lotz	Universitätsklinikum Frankfurt (University Hospital Frankfurt) (Uniklinik)
Dr Maximilian Malfertheiner<br>Esther Dreier<br>Dr Lars Maier	Universitätsklinikum Regensburg (Klinik für Innere Medizin II)
Dr Neurinda Permata Kusumastuti	University Airlangga Hospital (Paediatric)
Dr Colin McCloskey<br>Dr Al-Awwab Dabaliz<br>Dr Tarek B Elshazly<br>Josiah Smith	University Hospital Cleveland Medical Centre (UH Cleveland hospital)
Dr Konstanty S. Szuldrzynski<br>Dr Piotr Bielański	University Hospital in Krakow
Dr Yusuff Hakeem	University Hospitals of Leicester NHS Trust (Glenfield Hospital)
Dr Keith Wille	University of Alabama at Birmingham Hospital (UAB)
A/Prof Srinivas Murthy	University of British Columbia
Dr Ken Kuljit S. Parhar<br>Dr Kirsten M. Fiest<br>Cassidy Codan<br>Anmol Shahid	University of Calgary (Peter Lougheed Centre, Foothills Medical Centre, South Health Campus and Rockyview General Hospital)
Dr Mohamed Fayed<br>Dr Timothy Evans<br>Rebekah Garcia<br>Ashley Gutierrez<br>Hiroaki Shimizu	University of California, San Francisco-Fresno Clinical Research Centre
Dr Tae Song<br>Rebecca Rose	University of Chicago
Dr Suzanne Bennett<br>Denise Richardson	University of Cincinnati Medical Centre
Dr Giles Peek	University of Florida
Dr Lovkesh Arora<br>Kristina Rappapport<br>Kristina Rudolph	University of Iowa

Zita Sibenaller<br>Lori Stout<br>Alicia Walter	
Dr Daniel Herr<br>Nazli Vedadi	University of Maryland - Baltimore
Prof Robert Bartlett	University of Michigan Medical Center
Prof Antonio Pesenti	University of Milan
Dr Lace Sindt<br>Cale Ewald<br>Julie Hoffman<br>Sean Rajnic<br>Shaun Thompson	University of Nebraska Medical Centre
Dr Ryan Kennedy	University of Oklahoma Health Sciences Centre (OU)
Dr Matthew Griffee<br>Dr Anna Ciullo<br>Yuri Kida	University of Utah Hospital
Dr Ricard Ferrer Roca<br>Cynthia Alegre<br>Dr Sofia Contreras<br>Dr JordI Riera	Vall d'Hebron University Hospital, Barcelona
Dr Christy Kay<br>Irene Fischer<br>Elizabeth Renner	Washington University in St. Louis/ Barnes Jewish Hospital
Dr Hayato Taniguci	Yokohama City University Medical Center
Prof John Fraser<br>A/Prof Gianluigi Li Bassi<br>Dr Jacky Suen<br>Prof Adrian Barnett<br>Dr Nicole White<br>Dr Kristen Gibbons<br>Simon Forsyth<br>Amanda Corley	COVID-19 Critical Care Consortium

India Pearse<br>Samuel Hinton<br>Gabriella Abbate<br>Halah Hassan<br>Dr Silver Heinsar<br>Varun A Karnik<br>Dr Katrina Ki<br>Hollier F. O'Neill<br>Dr Nchafatso Obonyo<br>Dr Leticia Pretti Pimenta<br>Janice D. Reid<br>Dr Kei Sato<br>Dr Kiran Shekar<br>Aapeli Vuorinen<br>Dr Karin S. Wildi<br>Emily S. Wood<br>Dr Stephanie Yerkovich	
James Lee<br>Daniel Plotkin<br>Barbara Wanjiru Citarella<br>Laura Merson	ISARIC, Centre for Tropical Medicine and Global Health, University of Oxford, Oxford, UK

Collaborators

Prefix/First Name/Last Name	Site Name
Dr Emma Hartley	Aberdeen Royal Infirmary (Foresterhill Health Campus)

Bastian Lubis	Adam Malik Hospital
Takanari Ikeyama	Aichi Childrens Health and Medical Center
Balu Bhaskar	American Hospital
Dr Jae-Seung Jung	Anam Korea University Hospital
Sandra Rossi Marta<br>Fabio Guarracino	Azienda Ospedaliero Universitaria Parma
Prof Fabio Guarracino	Azienda Ospedaliero Universitaria Pisana
Stacey Gerle	Banner University Medical Centre
Emily Coxon	Baptist Health Louisville
Dr Bruno Claro	Barts Hospital
Dr. Gonzo Gonzalez-Stawinski	Baylor All Saints Medical Centre, Forth Worth
Daniel Loverde	Billings Clinic
Dr Vieri Parrini	Borgo San Lorenzo Hospital
Dr Angela McBride	Brighton and Sussex Medical School
Kathryn Negaard<br>Dr Phillip Mason	Brooke Army Medical Centre
Dr Angela Ratsch	Bundaberg Hospital
Ahmad Abdelaziz	Cairo University Hospital
Juan David Uribe	Cardio VID
Dr Adriano Peris	Careggi Hospital
Mark Sanders	Cedar Park Regional Medical Center
Dominic Emerson	Cedars-Sinai Medical Centre
Prof Pedro Povoa	Centro Hospitalar de Lisboa
Dr Roland Francis	Charite-Univerrsitatsmedizi n Berlin
Ali Cherif	Charles Nicolle University Hospital
Dr Sunimol Joseph	Children’s Health Ireland (CHI) at Crumlin
Dr Matteo Di Nardo	Children’s Hospital Bambino Gesù
Micheal Heard	Children's Healthcare of Atlanta- Egleston Hospital
Kimberly Kyle	Children's Hospital

Ray A Blackwell	Christiana Care Health System's Centre for Heart and Vascular Health
Dr Michael Piagnerelli<br>Dr Patrick Biston	CHU de Charleroi
Hye Won Jeong	Chungbuk National University Hospital
Reanna Smith	Cincinnati Children's
Yogi Prawira	Cipto Mangunkusumo Hospital
Dr Giorgia Montrucchio	Città della Salute e della Scienza Hospital – Turin, Italy
Nadeem Rahman<br>Vivek Kakar	Cleveland Clinic, Abu Dhabi
Dr Michael Piagnerelli<br>Dr Josefa Valenzuela Sarrazin	Clinica Las Condes
Dr Arturo Huerta Garcia	Clínica Sagrada Família
Dr Bart Meyns	Collaborative Centre Department Cardiac Surgery, UZ Leuven
Marsha Moreno	Dignity Health Medical Group- Dominican
Rajat Walia	Dignity Health St. Joseph's Hospital and Medical Center (SJHMC)
Dr Annette Schweda	Donaustauf hospital
Cenk Kirakli	Dr. Suat Seren Chest Diseases and Surgery Practice and Training Centre
Estefania Giraldo	Fundación Clinica Shaio (Shaio Clinic)
Dr Wojtek Karolak	Gdansk Medical University
Dr Martin Balik	General University Hospital
Elizabeth Pocock	George Washington University Hospital
Evan Gajkowski	Giesinger Medical Centre
Kanamoto Masafumi	Gunma University Graduate School of Medicine
Dr Nicholas Barrett	Guy's and St Thomas NHS Foundation Trust Hospital
Yoshihiro Takeyama	Hakodate City Hospital
Sunghoon Park	Hallym University Sacred Heart Hospital
Faizan Amin	Hamilton General Hospital
Dr Fina Meilyana Andriyani	Hasan Sadikin Hospital (Paediatric)
Dr Serhii Sudakevych	Heart Institute Ministry of Health of Ukraine

Dr Angela Ratsch	Hervey Bay Hospital
Patrícia Schwarz<br>Ana Carolina Mardini	Hospital de Clínicas de Porto Alegre
Ary Serpa Neto	Hospital Israelita Albert Einstein
Dr Andrea Villoldo	Hospital Privado de Comunidad
Alexandre Siciliano Colafranceschi	Hospital Pro Cardíaco
Dr Alejandro Ubeda Iglesias	Hospital Punta de Europa
Lívia Maria Garcia Melro<br>Giovana Fioravante Romualdo	Hospital Samaritano Paulista
Diego Gaia	Hospital Santa Catarina
Helmgton Souza	Hospital Santa Marta
Dr Diego Bastos	Hospital Cura D'ars Fortaleza
Filomena Galas	Hospital Sirio Libanes
Dr Rafael Máñez Mendiluce	Hospital Universitario de Bellvitge
Alejandra Sosa	Hospital Universitario Esperanza (Universidad Francisco Marroquin)
Dr Ignacio Martinez	Hospital Universitario Lucus Augusti
Hiroshi Kurosawa	Hyogo Prefectural Kobe Children's Hospital
Juan Salgado	Indiana University Health
Dr Beate Hugi-Mayr	Inselspital University Hospital
Eric Charbonneau	Institut Universitaire de Cardiologie et de Pneumologie de Quebec - Universite Laval
Vitor Salvatore Barzilai	Instituto de Cardiologia do Distrito Federal - ICDF
Veronica Monteiro	Instituto de Medicina Integral Prof. Fernando Figueira (IMIP)
Rodrigo Ribeiro de Souza	Instituto Goiano de Diagnostico Cardiovascular (IGDC)
Michael Harper	INTEGRIS Baptist Medical Center
Hiroyuki Suzuki	Japan Red Cross Maebashi Hospital
Celina Adams	John C Lincoln Medical Centre
Dr Jorge Brieva	John Hunter Hospital
George Nyale	Kenyatta National Hospital (KNH)
Jihan Fatani	King Abdullah Medical City Specialist Hospital

Dr Faisal Saleem Eltatar	
Dr. Husam Baeissa	King Abdullah Medical Complex
Ayman AL Masri	King Salman Hospital NWAF
Yee Hui Mok	KK Women's and Children's Hospital
Masahiro Yamane	KKR Medical Center
Hanna Jung	Kyung Pook National University Hospital
Rhonda Bakken	M Health Fairview
Dr Tim Felton	Manchester University NHS Foundation Trust - Wythenshawe
Lorenzo Berra	Massachusetts General Hospital
Gordan Samoukoviv<br>Dr Josie Campisi	McGill University Health Centre
Bobby Shah	Medanta Hospital
Arpan Chakraborty	Medica Super speciality Hospital
Monika Cardona	Medical University of South Carolina
Harsh Jain	Mercy Hospital of Buffalo
Dr Asami Ito	Mie University Hospital
Brahim Housni	Mohammed VI University hospital
Sennen Low	National Centre for Infectious Diseases
Dr. Koji Iihara	National Cerebral and Cardiovascular Center
Joselito Chavez	National Kidney and Transplant Institute
Dr Kollengode Ramanathan	National University Hospital, Singapore
Gustavo Zabert	National University of Comahue
Krubin Naidoo	Nelson Mandela Children's Hospital
Dr Ian Seppelt	Nepean Hospital
Marlice VanDyk<br>Sarah MacDonald	Netcare Unitas ECMO Centre
Randy McGregor	Northwestern Medicine
Teka Siebenaler	Norton Children's Hospital
Hannah Flynn	Novant Health (NH) Presbyterian Medical Centre

Julia Garcia-Diaz<br>Catherine Harmon	Ochsner Clinic Foundation
Kristi Lofton	Ochsner LSA Health Shreveport
Toshiyuki Aokage	Okayama University Hospital
Kazuaki Shigemitsu	Osaka City General Hospital
Dr Andrea Moscatelli	Ospedale Gaslini
Dr Giuseppe Fiorentino	Ospedali dei Colli
Dr Matthias Baumgaertel	Paracelsus Medical University Nuremberg
Serge Eddy Mba	Parirenyatwa General Hospital
Jana Assy	Pediatric and Neonatal Cardiac intensive care at the American University
Holly Roush	Penn State Heath S. Hershey Medical Centre
Kay A Sichting	Peyton Manning Children's Hospital
Dr Francesco Alessandri	Policlinico Umberto, Sapienza University of Rome
Debra Burns	Presbyterian Hospital, New York/ Weill Cornell Medical Centre
Ahmed Rabie	Prince Mohammed bin Abdulaziz Hospital
Dr Gavin Salt	Prince of Wales
Carl P. Garabedian	Providence Sacred Heart Children's Hospital
Dr Jonathan Millar<br>Dr Malcolm Sim	Queen Elizabeth II University Hospital
Dr Adrian Mattke	Queensland Children’s Hospital
Dr Danny McAuley	Queens University of Belfast
Jawad Tadili	Rabat university hospital
Dr Tim Frenzel	Radboud University Medical Centre
Aaron Blandino Ortiz	Ramón y Cajal University Hospital
Jackie Stone	Rapha Medical Centre
Dr Antony Attokaran	Rockhampton Hospital
Dr Michael Farquharson	Royal Adelaide Hospital
Dr Brij Patel	Royal Brompton &Harefield NHS Foundation Trust
Derek Gunning	Royal Columbian Hospital

Dr Kenneth Baillie	Royal Infirmary Edinburgh
Dr Pia Watson	Sahlgrenska University Hospital
Kenji Tamai	Saiseikai Yokohamashi Tobu Hospital
Dr Gede Ketut Sajinadiyasa<br>Dr Dyah Kanyawati	Sanglah General Hospital
Marcello Salgado	Santa Casa de Misericordia de Juiz de Fora
Assad Sassine	Santa Casa de Misericórdia de Vitoria
Dr Bhirowo Yudo	Sardjito Hospital
Scott McCaul	Scripps Memorial Hospital La Jolla
Bongjin Lee	Seoul National University Children's Hospital
Dr Young-Jae Cho<br>Dr Sang Min Lee	Seoul National University Hospital
Yoshiaki Iwashita	Shimane University Hospital
Laveena munshi	Sinai Health Systems (Mount Sinai Hospital)
Dr Neurinda Permata Kusumastuti<br>Dr Bambang Pujo Semedi	Soetomo General Hospital (FK UNAIR)
Dr Nicole Van Belle	St. Antonius Hospital
Daniel Marino	St. Christopher's Hospital for Children
Ignacio Martin-Loeches	St James’s University Hospital
Dr Hergen Buscher	St Vincent’s Hospital, Sydney
Dr Lenny Ivatt	Swansea Hospital
Chia Yew Woon	Tan Tock Seng Hospital
Hyun Mi Kang	The Catholic University of Seoul St Mary Hospital
Erskine James	The Medical Centre Navicent Health
Nawar Al-Rawas	Thomas Jefferson University Hospital
Tomoyuki Endo	Tohoku Medical and Pharmaceutical University
Dr Yudai Iwasaki	Tohoku University
Kenny Chan King-Chung	Tuen Mun Hospital
Dr Vadim Gudzenko	UCLA Medical Centre (Ronald Regan)

Dr Beate Hugi-Mayr	Universitätsspital Bern, Universitätsklinik für Herz- und Gefässchirurgie
Dr Fabio Taccone	Universite Libre de Bruxelles
Dr Fajar Perdhana	University Airlangga Hospital (Adult)
Yoan Lamarche	University de Montreal (Montreal Heart Institute)
Dr Joao Miguel Ribeiro	University Hospital CHLN
Dr Nikola Bradic	University Hospital Dubrava
Dr Klaartje Van den Bossche	University Hospital Leuven
Gurmeet Singh	University of Aberta (Mazankowski Heart Institute)
Dr Gerdy Debeuckelaere	University of Antwerp
Dr Henry T. Stelfox	University of Calgary and Alberta Health Services
Cassia Yi	University of California at San Diego
Jennifer Elia	University of California, Irvine
Shu Fang	University of Hong Kong
Thomas Tribble	University of Kentucky Medical Center
Shyam Shankar	University of Missouri
Dr Paolo Navalesi	University of Padova
Raj Padmanabhan	University of Pittsburgh Medical Centre
Bill Hallinan	University of Rochester Medical Centre (UR Medicine)
Luca Paoletti	University of South Carolina
Yolanda Leyva	University of Texas Medical Branch
Tatuma Fykuda	University of the Ryukyus
Jillian Koch	University of Wisconsin & American Family Children's Hospital
Amy Hackman	UT Southwestern
Lisa Janowaik	UTHealth (University of Texas)
Jennifer Osofsky	Vassar Brothers Medical Center (VBMC)
A/Prof Katia Donadello	Verona Integrated University Hospital
Josh Fine	WellSpan Health - York Hospital
Dr Benjamin Davidson	Westmead Hospital
Andres Oswaldo Razo Vazquez	Yale New Haven Hospital

Document S3. Case report form regarding demographics, comorbidities, medications, laboratory values, complications, and outcomes

World Health Organization ISARIC

COVID-19 CORE CASE REPORT FORM

ACUTE RESPIRATORY INFECTION CLINICAL CHARACTERISATION DATA TOOL

DESIGN OF THIS CASE REPORT FORM (CRF)

This CRF is set up in modules to be used for recording data on the ISARIC_nCov Core Database or for independent studies.

Module 1 and Module 2 complete on the first day of presentation/admission or on first day of COVID-19 assessment.
Module 2 also complete on first day of admission to ICU or high dependency unit. In addition, complete daily for as many days as resources allow up to a maximum of 14 days. Continue to follow-up patients who transfer between wards.
Module 3 (Outcome) complete at discharge or death

GENERAL GUIDANCE

- The CRF is designed to collect data obtained through examination, interview and review of hospital notes. Data may be collected prospectively or retrospectively if the patient is enrolled after the admission date.
- Participant Identification Numbers consist of a 5 digit site code and a 4 digit participant number. You can obtain a site code and registering on the data management system by contacting ncov@isaric.org. Participant numbers should be assigned sequentially for each site beginning with 0001. In the case of a single site recruiting participants on different wards, or where it is otherwise difficult to assign sequential numbers, it is acceptable to assign numbers in blocks or incorporating alpha characters. E.g. Ward X will assign numbers from 0001 or A001 onwards and Ward Y will assign numbers from 5001 or B001 onwards. Enter the Participant Identification Number at the top of every page.
- Printed paper CRFs may be used for later transfer of the data onto the electronic database.
- For participants who return for re-admission to the same site, **start a new form with the same Participant Identification Number**. Please check “YES-admitted previously” in the ONSET & ADMISSION section. Enter as 2 separate entries in the electronic database.
- For participants who transfer between two sites that are both collecting data on this form, it is preferred to have the data entered by a single site as a single admission, under the same Participant Identification Number. When this is not possible, the first site should record “Transfer to other facility” as an OUTCOME, and the second site should start a new form with a new patient number and indicate “YES-transferred” in ONSET & ADMISSION.
- Complete every line of every section, except where the instructions say to skip a section based on a response.
- Selections with circles (**O**) are single selection answers (choose one answer only). Selections with square boxes (☐) are multiple selection answers (choose as many answers as are applicable).
- Mark ‘Not done’ for any results of laboratory values that are not available, not applicable or unknown.
- Avoid recording data outside of the dedicated areas. Sections are available for recording additional information.
- If using paper CRFs, we recommend writing clearly in ink, using BLOCK-CAPITAL LETTERS.
- Place an (X) when you choose the corresponding answer. To make corrections, strike through (-------) the data you wish to delete and write the correct data above it. Please initial and date all corrections.
- Please keep all of the sheets for a single participant together e.g. with a staple or participant-unique folder.
- Please transfer all paper CRF data to the electronic database. All paper CRFs needs to be stored locally, do not send any forms to us. Data are accepted only via secure electronic database.
- Please enter data on the electronic data capture system at https://ncov.medsci.ox.ac.uk/. If your site would like to collect data independently, we are happy to support the establishment of locally hosted databases.
- Please contact us at ncov@isaric.org if you need help with databases, if you have comments and to let us know that you are using the forms.

Adapted from SPRINT SARI CRF by ISARIC. Licensed under a Creative Commons Attribution-ShareAlike 4.0 International License by ISARIC on behalf of Oxford University.

World Health Organization ISARIC

PARTICIPANT IDENTIFICATION #: [___][___][___][___][___]--- [___][___][___][___]

MODULE 1: PRESENTATION/ADMISSION CASE REPORT FORM

CLINICAL INCLUSION CRITERIA

Suspected or confirmed novel coronavirus (COVID-19) infection: ○YES ○NO

DEMOGRAPHICS

Clinical centre name:____**Country:**____

Enrolment date /first COVID-19 assessment date: [_D_][_D_]/[_M_][_M_]/[_2_][_0_][_Y_][_Y_]

Ethnic group *(check all that apply)*: ☐Arab ☐Black ☐East Asian ☐South Asian ☐ West Asian ☐Latin American ☐White

☐Aboriginal/First Nations ☐Other: ___________________________ ○Unknown

Employed as a Healthcare Worker? ○YES ○NO ○Unknown **Employed in a microbiology laboratory?** ○YES ○NO ○Unknown

Sex at Birth: ○Male ○Female ○Not specified/Unknown **Age** [___][___][___]years OR [___][___]months

Pregnant? ○YES ○NO ○Unknown **If YES: Gestational weeks assessment:** [___][___] weeks

POST PARTUM? ○YES ○NO ○Unknown *(if NO or Unknown skip this section)*

Pregnancy Outcome: ○Live birth ○Still birth **Delivery date:** [_D_][_D_]/[_M_][_M_]/[_2_][_0_][_Y_][_Y_]

Baby tested for COVID-19/SARS-CoV-2 infection? ○YES ○NO ○Unknown

If YES, result of test: ○Positive ○Negative ○Unknown (If Positive, complete a separate CRF for baby)

INFANT – Less than 1 year old? ○YES ○NO *(If NO skip this section)*

Birth weight: [___][___].[___]○kg or ○lbs ○Unknown

Gestational outcome: ○ Term birth (≥37wk GA) ○Preterm birth (<37wk GA) ○Unknown

Breastfed? ○YES-currently breastfeeding ○YES-breastfeeding discontinued ○NO ○Unknown

Vaccinations appropriate for age/country? ○YES ○NO ○Unknown

ONSET & ADMISSION

Onset date of first/earliest symptom: [_D_][_D_]/[_M_][_M_]/[_2_][_0_][_Y_][_Y_]

Most recent presentation/admission date at this facility: [_D_][_D_]/[_M_][_M_]/[_2_][_0_][_Y_][_Y_]

Was the patient admitted previously or transferred from any other facility during this illness episode?

○YES-admitted previously to this facility ○YES–transferred from other facility ○NO ○Unknown

SIGNS AND SYMPTOMS AT HOSPITAL ADMISSION *(first available data at presentation/admission – within 24 hours)*

Temperature: [____][____][____].[____]○°C *or* ○°F

HR: [____][____][____]beats per minute **RR:** [____][____]breaths per minute

Systolic BP: [___][___][___]mmHg **Diastolic BP:** [____][____][____]mmHg

Oxygen saturation: [___][___][___]% **On:** ○Room air ○Oxygen therapy ○Unknown

Sternal capillary refill time >2sec. ○YES ○NO ○Unknown **Height:** [___][___][___]cm **Weight:** [___][___][___]kg

Adapted from SPRINT SARI CRF by ISARIC. Licensed under a Creative Commons Attribution-ShareAlike 4.0 International License by ISARIC on behalf of Oxford University.

MODULE 1: PRESENTATION/ADMISSION CASE REPORT FORM

**SIGNS AND SYMPTOMS ON ADMISSION** *(Unk = Unknown)*			
History of fever	○YES ○NO ○Unk	Fatigue / Malaise	○YES ○NO ○Unk
Cough ○YES-non-productive ○YES-productive ○YES-with haemoptysis ○NO ○Unk		Anorexia	○YES ○NO ○Unk
		Altered consciousness/confusion	○YES ○NO ○Unk
Sore throat	○YES ○NO ○Unk	Muscle aches (myalgia)	○YES ○NO ○Unk
Runny nose (rhinorrhoea)	○YES ○NO ○Unk	Joint pain (arthralgia)	○YES ○NO ○Unk
Wheezing	○YES ○NO ○Unk	Inability to walk	○YES ○NO ○Unk
Shortness of breath	○YES ○NO ○Unk	Abdominal pain	○YES ○NO ○Unk
Lower chest wall indrawing	○YES ○NO ○Unk	Diarrhoea	○YES ○NO ○Unk
Chest pain	○YES ○NO ○Unk	Vomiting / Nausea	○YES ○NO ○Unk
Conjunctivitis	○YES ○NO ○Unk	Skin rash	○YES ○NO ○Unk
Lymphadenopathy	○YES ○NO ○Unk	Bleeding (Haemorrhage)	○YES ○NO ○Unk
Headache	○YES ○NO ○Unk	If YES, specify site(s):	
Loss of smell (Anosmia)	○YES ○NO ○Unk	Other symptom(s)	○YES ○NO ○Unk
Loss of taste (Ageusia)	○YES ○NO ○Unk	If YES, specify:	
Seizures	○YES ○NO ○Unk		

**PRE-ADMISSION MEDICATION** *(taken within 14 days of admission/presentation at healthcare facility)*	
Angiotensin converting enzyme inhibitors (ACE inhibitors)	○YES ○NO ○Unk
Angiotensin II receptor blockers (ARBs)	○YES ○NO ○Unk
Non-steroidal anti-inflammatory (NSAIDs)	○YES ○NO ○Unk
Oral steroids	○YES ○NO ○Unk If YES, agent(s):
Other immunosuppressant agents (not oral steroids)	○YES ○NO ○Unk If YES, agent(s):
Antivirals	○YES ○NO ○Unk If YES, agent(s):
Antibiotics	○YES ○NO ○Unk If YES, agent(s):
Other targeted COVID-19 Medications	○YES ○NO ○Unk If YES, agent(s):

**CO-MORBIDITIES AND RISK FACTORS** *(existing prior to admission and ongoing)*			
Chronic cardiac disease *(not hypertension)*	○YES ○NO ○Unk	Chronic hematologic disease	○YES ○NO ○Unk
Hypertension	○YES ○NO ○Unk	AIDS / HIV ○YES-on ART ○YES-not on ART ○NO ○Unk	
Chronic pulmonary disease *(not asthma)*	○YES ○NO ○Unk	Diabetes Mellitus ○YES-Type 1 ○YES -Type 2 ○NO ○Unk	
Asthma *(physician diagnosed)*	○YES ○NO ○Unk	Rheumatologic disorder	○YES ○NO ○Unk
Chronic kidney disease	○YES ○NO ○Unk	Dementia	○YES ○NO ○Unk
Obesity *(as defined by clinical staff)*	○YES ○NO ○Unk	Tuberculosis	○YES ○NO ○Unk
Moderate or severe liver disease	○YES ○NO ○Unk	Malnutrition	○YES ○NO ○Unk
Mild liver disease	○YES ○NO ○Unk	Smoking ○YES ○Never smoked ○Former smoker ○Unk	
Asplenia	○YES ○NO ○Unk	Other relevant risk factor(s)	○YES ○NO ○Unk
Chronic neurological disorder	○YES ○NO ○Unk	If YES, specify:	
Malignant neoplasm	○YES ○NO ○Unk		

Adapted from SPRINT SARI CRF by ISARIC. Licensed under a Creative Commons Attribution-ShareAlike 4.0 International License by ISARIC on behalf of Oxford University.

PARTICIPANT IDENTIFICATION #: [___][___][___][___][___]--- [___][___][___][___]

MODULE 2: DAILY CASE REPORT FORM

Complete on the day of admission or first COVID-19 investigation, and on the first day of ICU admission (if different from day of admission). In addition, depending on available resources, complete every day for a maximum of 14 days, or for days when biochemical results are available.

SIGNS AND SYMPTOMS *(Record the worst value between 00:00 to 24:00 on day of assessment)(worst=furthest from normal range)*

DATE OF ASSESSMENT *(DD/MM/YYYY)*: [_D_][_D_]/[_M_][_M_]/[__2_][__0_][_Y_][_Y_]

Temperature: [___][___].[___] O°C *or* O°F **HR:** [___][___][___]beats per minute **RR:** [___][___][___]breaths per minute

Systolic BP: [___][___][___]mmHg **Diastolic BP:** [___][___][___]mmHg **Oxygen saturation SaO_2** [___][___][___]%

Any supplemental oxygen: FiO_2 *(0.21-1.0)* [___].[___][___] or [___][___] % or [___][___]L/min

Sternal capillary refill time >2seconds OYES ONO OUnknown

AVPU: Alert [___] Verbal[___] Pain [___] Unresponsive [___] **Glasgow Coma Score (GCS / 15)** [___][___]

Is the patient currently receiving, or has received *(between 00:00 to 24:00 on day of assessment)*

High-flow nasal cannula oxygen therapy? OYES ONO OUnknown

Non-invasive ventilation *(Any)*? OYES ONO OUnknown **If YES:** OBIPAP OCPAP OOther OUnknown

Invasive ventilation? OYES ONO OUnknown

Prone positioning? OYES ONO OUnknown

Inhaled Nitric Oxide? OYES ONO OUnknown

Tracheostomy inserted? OYES ONO OUnknown

Extra corporeal life support (ECLS/ ECMO)? OYES ONO OUnknown **If YES:** OVV OAV OCentral OUnknown

Renal replacement therapy (RRT) or dialysis? OYES ONO OUnknown

Any vasopressor/inotropic support? OYES ONO OUnknown *(if NO, select NO for the next 3 questions)*

- **Dopamine <5µg/kg/min OR Dobutamine OR milrinone OR levosimendan:** OYES ONO
- **Dopamine 5-15µg/kg/min OR Epinephrine/Norepinephrine < 0.1µg/kg/min OR vasopressin OR phenylephrine**: OYES ONO
- **Dopamine >15µg/k/min OR Epinephrine/Norepinephrine > 0.1µg/kg/min**: OYES ONO

Neuromuscular blocking agents? OYES ONO OUnknown

Other intervention(s) or procedure(s)? OYES ONO OUnknown If YES, Specify: ____________________________

____

Current admission to ICU/ITU/IMC/HDU? OYES ONO OUnknown ***(Record the worst value on day of assessment)***

PaO_2 *(at time nearest to the FiO_2 recorded at top of page)* [___][___][___]OkPa *or* OmmHg ONot done

PaO_2 sample type: OArterial OCapillary OUnknown

From same blood gas record as PaO_2:

PCO_2 ____________OkPa *or* OmmHg | **pH** ____________ | **HCO_3-** ____________mEq/L | **Base excess** ____________ mmol/L

Richmond Agitation-Sedation Scale (RASS) [___] or **Riker Sedation-Agitation Scale (SAS)** [___] OUnknown

Mean Arterial Blood Pressure [___][___][___]mmHg OUnknown

Urine flow rate [___][___][___][___][___]mL/24 hours O Check if estimated OUnknown

Adapted from SPRINT SARI CRF by ISARIC. Licensed under a Creative Commons Attribution-ShareAlike 4.0 International License by ISARIC on behalf of Oxford University.

MODULE 2: DAILY CASE REPORT FORM

Complete on the day of admission or first COVID-19 investigation, and on the first day of ICU admission (if different from day of admission). In addition, depending on available resources, complete every day for a maximum of 14 days, or for days when biochemical results are available.

LABORATORY RESULTS (on admission, on any admission to ICU, then daily) – complete every line

DATE OF ASSESSMENT *(DD/MM/YYYY):* [_D_][_D_]/[_M_][_M_]/[_2_][_0_][_Y_][_Y_]

Record the worst value between 00:00 to 24:00 on day of assessment (if Not Available write 'N/A'):

LABORATORY RESULTS *(*record units if different from those listed)*

Parameter	Value*	Not done	Parameter	Value*	Not done
Haemoglobin (g/L)		○	Urea (BUN) (mmol/L)		○
WBC count (x10^9/L)		○	Lactate (mmol/L)		○
Lymphocyte count (10^9/L)		○	Creatinine (µmol/L)		○
Neutrophil count (10^9/L)		○	Sodium (mmol/L)		○
Haematocrit (%)		○	Potassium (mmol/L)		○
Platelets (x10^9/L)		○	Procalcitonin (ng/mL)		○
APTT (seconds)*)*		○	CRP (mg/L)		○
APTR		○	LDH (U/L)		○
PT (seconds)		○	Creatine kinase (U/L)		○
INR		○	Troponin I (ng/mL)		○
ALT/SGPT (U/L)		○	D-dimer (mg/L)		○
Total bilirubin (µmol/L)		○	Ferritin (ng/mL)		○
AST/SGOT (U/L)		○	IL-6 (pg/mL)		○
Glucose (mmol/L)		○			

Adapted from SPRINT SARI CRF by ISARIC. Licensed under a Creative Commons Attribution-ShareAlike 4.0 International License by ISARIC on behalf of Oxford University.

MODULE 3: OUTCOME CASE REPORT FORM

TREATMENT: At ANY time during hospitalisation, did the patient receive/undergo:

Any Oxygen therapy? ○YES ○NO ○Unknown **If YES, total duration:** _________days ○Unknown

Maximum O_2 flow volume: ○ <2 L/min ○2-5 L/min ○6-10 L/min ○11-15 L/min ○>15 L/min

Non-invasive ventilation? *(Any)* ○YES ○NO ○Unknown **If YES, total duration:** _________days ○Unknown

Invasive ventilation? *(Any)* ○YES ○NO ○Unknown **If YES, total duration:** _________days ○Unknown

Prone Positioning? ○YES ○NO ○Unknown **If YES, total duration:** _________days ○Unknown

Inhaled Nitric Oxide? ○YES ○NO ○Unknown

Tracheostomy inserted? ○YES ○NO ○Unknown

Extracorporeal support (ECMO)? ○YES ○NO ○Unknown **If YES, total duration:** _________days ○Unknown

Renal replacement therapy (RRT) or dialysis? ○YES ○NO ○Unknown

Inotropes/vasopressors? ○YES ○NO ○Unknown **If YES, total duration:** _________days ○Unknown

ICU or High Dependency Unit admission? ○YES ○NO ○Unknown **If YES, total duration:** _________days ○Unknown

If YES, date of ICU admission: [_D_][_D_]/[_M_][_M_]/[_2_][_0_][_Y_][_Y_] ○Unknown

date of ICU discharge: [_D_][_D_]/[_M_][_M_]/[_2_][_0_][_Y_][_Y_] ○Unknown

COMPLICATIONS: At any time during hospitalisation did the patient experience: *(Unk = Unknown)*

Viral pneumonia/pneumonitis	○YES ○NO ○Unk	Stroke / Cerebrovascular accident	○YES ○NO ○Unk
Bacterial pneumonia	○YES ○NO ○Unk	Meningitis / Encephalitis	○YES ○NO ○Unk
Acute Respiratory Distress Syndrome	○YES ○NO ○Unk	Bacteremia	○YES ○NO ○Unk
If YES, specify: ○ Mild ○ Moderate ○ Severe ○Unk		Coagulation disorder / DIC	○YES ○NO ○Unk
Pneumothorax	○YES ○NO ○Unk	Pulmonary embolism	○YES ○NO ○Unk
Pleural effusion	○YES ○NO ○Unk	Anemia	○YES ○NO ○Unk
Cryptogenic organizing pneumonia (COP)	○YES ○NO ○Unk	Rhabdomyolysis / Myositis	○YES ○NO ○Unk
Bronchiolitis	○YES ○NO ○Unk	Acute renal injury/ Acute renal failure	○YES ○NO ○Unk
Cardiac arrest	○YES ○NO ○Unk	Gastrointestinal haemorrhage	○YES ○NO ○Unk
Myocardial infarction	○YES ○NO ○Unk	Pancreatitis	○YES ○NO ○Unk
Cardiac ischaemia	○YES ○NO ○Unk	Liver dysfunction	○YES ○NO ○Unk
Cardiac arrhythmia	○YES ○NO ○Unk	Hyperglycemia	○YES ○NO ○Unk
Myocarditis / Pericarditis	○YES ○NO ○Unk	Hypoglycemia	○YES ○NO ○Unk
Endocarditis	○YES ○NO ○Unk	Other	
Cardiomyopathy	○YES ○NO ○Unk	If YES specify:	
Congestive heart failure	○YES ○NO ○Unk		
Seizure	○YES ○NO ○Unk		

Adapted from SPRINT SARI CRF by ISARIC. Licensed under a Creative Commons Attribution-ShareAlike 4.0 International License by ISARIC on behalf of Oxford University.

MODULE 3: OUTCOME CASE REPORT FORM

DIAGNOSTICS

Was patient clinically diagnosed with COVID-19? ○YES ○NO ○Unknown

Was pathogen testing done during this illness episode? ○YES *(complete section)* ○NO ○Unknown

Coronavirus: ○Positive ○Negative ○Not done **If Positive:** ○COVID-2019/ SARS-CoV2 ○MERS CoV ○Other CoV: ____________________ ○Unknown

Influenza : ○Positive ○Negative ○Not done **If Positive:** ○A/H3N2 ○A/H1N1pdm09 ○A/H7N9 ○A/H5N1 ○A-not typed ○B ○Other:__________________________ ○Unknown

RSV: ○Positive ○Negative ○Not done

Adenovirus: ○Positive ○Negative ○Not done

Bacteria: ○Positive ○Negative ○Not done **If Positive, specify**: _________________ ________________ ○Unknown

Other pathogen/s detected: ○YES ○NO ○Unknown **If YES, specify all:**________________________________○Unknown

****

Clinical pneumonia diagnosed? ○YES ○NO ○Unknown

Chest X-Ray performed? ○YES ○NO ○Unknown **If Yes: Were infiltrates present?** ○YES ○NO ○Unknown

CT performed? ○YES ○NO ○Unknown **If Yes: Were infiltrates present?** ○YES ○NO ○Unknown

Collection Date *(DD/MM/YYYY)*	Biospecimen Type	Laboratory test Method	Result	Pathogen Tested/Detected
_D_ _D_ /_M_ _M_ /20_Y_ _Y_	○Nasal/NP swab ○Throat swab<br>○Combined nasal/NP+throat swab<br>○Sputum ○BAL ○ETA ○Urine<br>○Feces/rectal swab ○Blood<br>○Other, *Specify:* ____________________	○PCR<br>○Culture<br>○Other, *Specify:*<br>______________	○Positive<br>○Negative<br>○Unknown	________________
_D_ _D_ /_M_ _M_ /20_Y_ _Y_	○Nasal/NP swab ○Throat swab<br>○Combined nasal/NP+throat swab<br>○Sputum ○BAL ○ETA ○Urine<br>○Feces/rectal swab ○Blood<br>○Other, *Specify:* ____________________	○PCR<br>○Culture<br>○Other, *Specify:*<br>______________	○Positive<br>○Negative<br>○Unknown	________________
_D_ _D_ /_M_ _M_ /20_Y_ _Y_	○Nasal/NP swab ○Throat swab<br>○Combined nasal/NP+throat swab<br>○Sputum ○BAL ○ETA ○Urine<br>○Feces/rectal swab ○Blood<br>○Other, *Specify:* ____________________	○PCR<br>○Culture<br>○Other, *Specify:*<br>______________	○Positive<br>○Negative<br>○Unknown	________________
_D_ _D_ /_M_ _M_ /20_Y_ _Y_	○Nasal/NP swab ○Throat swab<br>○Combined nasal/NP+throat swab<br>○Sputum ○BAL ○ETA ○Urine<br>○Faeces/rectal swab ○Blood<br>○Other, *Specify:* ____________________	○PCR<br>○Culture<br>○Other, *Specify:*<br>______________	○Positive<br>○Negative<br>○Unknown	________________
_D_ _D_ /_M_ _M_ /20_Y_ _Y_	○Nasal/NP swab ○Throat swab<br>○Combined nasal/NP+throat swab<br>○Sputum ○BAL ○ETA □Urine<br>○Feces/rectal swab ○Blood<br>○Other, *Specify:* ____________________	○PCR<br>○Culture<br>○Other, *Specify:*<br>______________	○Positive<br>○Negative<br>○Unknown	________________

Adapted from SPRINT SARI CRF by ISARIC. Licensed under a Creative Commons Attribution-ShareAlike 4.0 International License by ISARIC on behalf of Oxford University.

MODULE 3: OUTCOME CASE REPORT FORM

MEDICATION: While hospitalised or at discharge, were any of the following administered? *(Unk=Unknown)*

Antiviral or COVID-19 targeted agent? ○YES ○NO ○Unknown **If YES, specify all agents and duration:**

☐**Ribavirin** **Date commenced** [_D_][_D_]/[_M_][_M_]/[_2_][_0_][_Y_][_Y_] **Duration**: _______ days ○Unk

☐ **Lopinavir/Ritonavir** **Date commenced** [_D_][_D_]/[_M_][_M_]/[_2_][_0_][_Y_][_Y_] **Duration**: _______ days ○Unk

☐**Remdesivir** **Date commenced** [_D_][_D_]/[_M_][_M_]/[_2_][_0_][_Y_][_Y_] **Duration**: _______ days ○Unk

☐**Interferon alpha** **Date commenced** [_D_][_D_]/[_M_][_M_]/[_2_][_0_][_Y_][_Y_] **Duration**: _______ days ○Unk

☐**Interferon beta** **Date commenced** [_D_][_D_]/[_M_][_M_]/[_2_][_0_][_Y_][_Y_] **Duration**: _______ days ○Unk

☐ **Chloroquine/hydroxychloroquine** **Date commenced** [_D_][_D_]/[_M_][_M_]/[_2_][_0_][_Y_][_Y_] **Duration**: _______days ○Unk

☐**Other** _____________________ **Date commenced** [_D_][_D_]/[_M_][_M_]/[_2_][_0_][_Y_][_Y_] **Duration**: _______days ○Unk

****

Antibiotic? ○YES ○NO ○Unk **If yes, specify all:**

Agent:______________________ **Date commenced** [_D_][_D_]/[_M_][_M_]/[_2_][_0_][_Y_][_Y_] **Duration:** ____ days ○Unk

Agent:______________________ **Date commenced** [_D_][_D_]/[_M_][_M_]/[_2_][_0_][_Y_][_Y_] **Duration:** ____ days ○Unk

Agent:______________________ **Date commenced** [_D_][_D_]/[_M_][_M_]/[_2_][_0_][_Y_][_Y_] **Duration:** ____ days ○Unk

****

Corticosteroid? ○YES ○NO ○Unk **If YES, Route:** ☐Oral ☐Intravenous (IV) ☐Inhaled ○Unk

If YES Oral or IV, please provide agent: ___________________________________ **and max. daily dose & unit:**______________

Date commenced [_D_][_D_]/[_M_][_M_]/[_2_][_0_][_Y_][_Y_] ○Unk **Duration**: _______ days ○Unk

****

Heparin? ○YES ○NO ○Unk **If YES, Route:** ☐Subcutaneous ☐Intravenous (IV) ○Unk

If YES: ☐Unfractionated ☐Low molecular weight ☐Fondaparinux ○Unk **Maximum daily dose & unit:**________________

Date commenced [_D_][_D_]/[_M_][_M_]/[_2_][_0_][_Y_][_Y_] ○Unk **Duration**: _______ days ○Unk

****

Antifungal agent? ○YES ○NO ○Unk

****

Other treatments administered for COVID-19 including experimental or compassionate use? ○YES ○NO ○Unk

If yes, specify agent, maximum daily does and duration:

Agent: __________________________ **Maximum daily dose & unit**: ________________________ ○Unk

Date of commencement [_D_][_D_]/[_M_][_M_]/[_2_][_0_][_Y_][_Y_] ○Unk **Duration**: _______ days ○Unk

Agent:__________________________ **Maximum daily dose & unit**: _________________________ ○Unk

Date of commencement [_D_][_D_]/[_M_][_M_]/[_2_][_0_][_Y_][_Y_] ○Unk **Duration**: _______ days ○Unk

OUTCOME

Outcome: ○Discharged alive ○Hospitalised ○Transfer to other facility ○Death ○Palliative discharge ○Unknown

Outcome date: [_D_][_D_]/[_M_][_M_]/[_2_][_0_][_Y_][_Y_] ○Unknown

If Discharged alive:

Ability to self-care at discharge versus before illness: ○Same as before illness ○Worse ○Better ○Unknown

Post-discharge treatment: Oxygen therapy? ○YES ○NO ○Unknown

Adapted from SPRINT SARI CRF by ISARIC. Licensed under a Creative Commons Attribution-ShareAlike 4.0 International License by ISARIC on behalf of Oxford University.

Document S4. Additional case report form regarding mechanical ventilation and ExtraCorporeal Membrane Oxygenation

Appendix B: Data Collection Form ECMOCARD

CORE CASE RECORD FORM (EOT ICU Admis)

1. UPON ICU ADMISSION – Please complete the below data as of the date and time of the patient's admission to the ICU

Is this patient's data collected using Full or Basic daily data forms?

- ◻ Full (forms completed every day of stay)
- ◻ Basic (reduced frequency of daily data collection)

DATE OF ICU ADMISSION: _____ / _____ / _____

1.1 HEIGHT (cm): ___________

If this data has already been entered into the 'Signs and Symptoms' section of the ISARIC CRF, please DO NOT re-enter the data here. Leave this '1.1 Height' box blank.

1.2 BODY WEIGHT (Kg): ___________

If this data has already been entered into the 'Signs and Symptoms" section of the ISARIC CRF, please DO NOT re-enter the data here. Leave this '1.2 Body Weight' box blank.

1.3 Arterial Hypertension

◻ Yes
◻ No

If this data has already been entered into the 'Co-Morbidities & Risk Factors' section of the ISARIC CRF, please DO NOT re-enter the data here. Leave this '1.3 Hypertension' box blank.

1.3a Chronic anti-hypertensive therapy?

◻ Yes
◻ No

1.3b Chronic anti-hypertensive therapy (if 'Yes' to 1.3. Please select up to three)

- ◻ Diuretics
- ◻ Calcium channel blockers
- ◻ ACE inhibitors

If this data has already been entered in the 'Pre-Admission Medication' section of the ISARIC CRF, please DO NOT re-enter the data here. Leave this 'ACE inhibitors' box blank.

☐ Angiotensin II receptor antagonists

If this data has already been entered in the 'Pre-Admission Medication' section of the ISARIC CRF, please DO NOT re-enter the data here. Leave this 'Angiotensin II receptor antagonists' box blank.

☐ Renin inhibitors

☐ Beta blockers

☐ Alpha blockers

☐ Vasodilators

☐ Aldosterone receptor antagonist

☐ Alpha-2 adrenergic receptor agonists

☐ Not applicable

1.4 PRE HOSPITAL ADMISSION CREATININE AVAILABLE?

☐ Yes
☐ No

1.4a PRE-HOSPITAL ADMISSION CREATININE: ___________

1.4a Creatinine units

☐ mg/dL
☐ umol/L

1.5 GASTROINTESTINAL AND PANCREATIC COMORBIDITIES

☐ Yes
☐ No

1.6 HEPATIC AND BILIARY COMORBIDITIES

☐ Yes
☐ No

1.7 HAEMATOLOGIC AND SPLEEN COMORBIDITIES

☐ Yes
☐ No

1.8 IMMUNOLOGICAL AND TRANSPLANT COMORBIDITIES

☐ Yes
☐ No

1.9 ENDOCRINOLOGICAL COMORBIDITIES

☐ Yes
☐ No

1.10 GENITO-URINARY COMORBIDITIES

☐ Yes
☐ No

1.11 CHRONIC ALCOHOL ABUSE

☐ Yes
☐ No

1.12 INTRAVENOUS DRUGS ABUSE

☐ Yes
☐ No

1.13 IMMUNO-COMPETENT

☐ Yes
☐ No

1.14 APACHE II SCORE: __________ (ONLY NUMBERS FROM 0 to 71)

APACHE II score can be calculated at the following link https://www.mdcalc.com/apache-ii-score

☐ Not available

1.15 SOFA SCORE: __________ (ONLY NUMBERS FROM 0 to 24)

SOFA score can be calculated at the following link https://www.mdcalc.com/sequential-organ-failure-assessment-sofa-score

☐ Not available

BLOOD GAS ANALYSIS (Qs 1.16 – 1.21) – Please document the values associated with the 'worst' blood gas analysis in the 6 hours prior to ICU admission. 'Worst' blood gas is defined as the blood gas with the lowest PaO2/FiO2 ratio.

1.16 ARTERIAL pH IN THE 6h BEFORE ICU ADMISSION: __________ (ONLY NUMBERS FROM 6.500 TO 7.600)

Please document the values associated with the 'worst' blood gas analysis in the 6 hours prior to ICU admission. 'Worst' is defined as the blood gas with the lowest PaO2/FiO2 ratio.

□ Not available

1.17 ARTERIAL PARTIAL PRESSURE OF OXYGEN IN THE 6h BEFORE ICU ADMISSION: _________

(ONLY NUMBERS FROM 10-500)

Units: ☐mmHg ☐kPa

Please document the values associated with the 'worst' blood gas analysis in the 6 hours prior to ICU admission. 'Worst' is defined as the blood gas with the lowest PaO2/FiO2 ratio.

□ Not available

1.18 ARTERIAL PARTIAL PRESSURE OF CARBON DIOXIDE IN THE 6h BEFORE ICU ADMISSION: __________

(ONLY NUMBERS FROM 10 TO 100)

Units: ☐mmHg ☐kPa

Please document the values associated with the 'worst' blood gas analysis in the 6 hours prior to ICU admission. 'Worst' is defined as the blood gas with the lowest PaO2/FiO2 ratio.

□ Not available

1.19 ARTERIAL BICARBONATE (HCO_3^-) IN THE 6h BEFORE ICU ADMISSION:________________

Units: ☐mEq/L ☐mmol/L

Please document the values associated with the 'worst' blood gas analysis in the 6 hours prior to ICU admission. 'Worst' is defined as the blood gas with the lowest PaO2/FiO2 ratio.

□ Not available

1.20 ARTERIAL BASE EXCESS IN THE 6h BEFORE ICU ADMISSION: _______________ mmol/L

(ONLY NUMBERS FROM -50 - +50)

Please document the values associated with the 'worst' blood gas analysis in the 6 hours prior to ICU admission. 'Worst' is defined as the blood gas with the lowest PaO2/FiO2 ratio.

□ Not available

1.21 LACTATE IN THE 6h BEFORE ICU ADMISSION: _______________ mmol/L

Please document the values associated with the 'worst' blood gas analysis in the 6 hours prior to ICU admission. 'Worst' is defined as the blood gas with the lowest PaO2/FiO2 ratio.

□ Not available

1.22 Troponin in the last 12 hours: (tick 2 at most)

- □ Troponin T: __________ (☐ng/mL or ☐ng/L) ONLY NUMBERS FROM 0 TO 150
- □ Troponin I: __________ (☐ng/mL or ☐ng/L) ONLY NUMBERS FROM 0 TO 150
- □ High sensitivity troponin T: __________ (☐ng/mL or ☐ng/L) ONLY NUMBERS FROM 0 TO 150
- □ High sensitivity troponin I: __________ (☐ng/mL or ☐ng/L) ONLY NUMBERS FROM 0 TO 150
- □ Not available

1.23 Cardiac BNP in the last 12 hours: __________ (picograms/mL) ONLY NUMBERS BETWEEN 0-30000

1.24 Upon ICU admission, did the patient present with cutaneous manifestations?

- □ Yes
- □ No
- □ Not available

If yes to 1.24a, type of cutaneous manifestations (please select up to three (3) options)

- □ Bullae
- □ Macules
- □ Nodules
- □ Papules
- □ Plaques
- □ Purpura
- □ Pustules
- □ Rash
- □ Scale
- □ Urticaria
- □ Vesicles
- □ Other: __________

If yes to 1.24b, specify the involved regions (please select up to three (3) options):

- □ Face
- □ Truck
- □ Upper limbs
- □ Hands
- □ Lower limbs
- □ Feet

CORE CASE RECORD FORM (EOT Mech Vent)

2. UPON COMMENCEMENT OF MECHANICAL VENTILATION - 'Mechanical ventilation' includes invasive mechanical ventilation via an endotracheal tube or tracheostomy only.
Importantly, this module will be active only when you click 'YES' in the field '1.17 Invasive ventilation' of the ISARIC form.

2.1 DATE OF START OF MECHANICAL VENTILATION: _____ / _____ / _____ (DD/MM/YY)

2.2 SITE OF INTUBATION

☐ Outside hospital
☐ Intensive Care Unit
☐ Emergency Department
☐ Hospital Ward
☐ Different hospital, then patient was transferred
☐ Other

2.3 TYPE OF INTUBATION

☐ Elective
☐ Emergent

2.4 CARDIAC ARREST

☐ Yes
☐ No

2.5 VENTILATORY SUPPORT BEFORE INTUBATION

☐ High-Flow Oxygen Ventilation
☐ Mask non-invasive ventilation
☐ Full Face-mask non-invasive ventilation
☐ Helmet non-invasive ventilation
☐ Simple face mask oxygen therapy
☐ Venturi mask oxygen therapy
☐ Non re-breather face mask oxygen therapy
☐ Nasal prongs oxygen therapy
☐ Other
☐ Not available

BLOOD GAS ANALYSIS (Qs 2.6 – 2.11) – Please document the values associated with the 'worst' blood gas analysis in the 6 hours prior to commencement of mechanical ventilation. 'Worst' blood gas is defined as the blood gas with the lowest PaO2/FiO2 ratio.

2.6 ARTERIAL pH IN THE 6 HOURS BEFORE START OF MV: __________ (ONLY NUMBERS FROM 6.500 TO 7.600)

Please document the values associated with the 'worst' blood gas analysis in the 6 hours prior to commencement of mechanical ventilation. 'Worst' is defined as the blood gas with the lowest PaO2/FiO2 ratio.

□ Not available

2.7 ARTERIAL PARTIAL PRESSURE OF OXYGEN (mmHg) IN THE 6 HOURS BEFORE START OF MV: __________ (ONLY NUMBERS FROM 20 TO 500)

Units: ☐mmHg ☐kPa

Please document the values associated with the 'worst' blood gas analysis in the 6 hours prior to commencement of mechanical ventilation. 'Worst' is defined as the blood gas with the lowest PaO2/FiO2 ratio.

□ Not available

2.8 ARTERIAL PARTIAL PRESSURE OF CARBON DIOXIDE IN THE 6 HOURS BEFORE START OF MV: __________ (ONLY NUMBERS FROM 10 TO 100)

Units: ☐mmHg ☐kPa

Please document the values associated with the 'worst' blood gas analysis in the 6 hours prior to commencement of mechanical ventilation. 'Worst' is defined as the blood gas with the lowest PaO2/FiO2 ratio.

□ Not available

2.9 ARTERIAL HCO_3^- IN THE 6 HOURS BEFORE START OF MV: ___________________(ONLY NUMBERS FROM 1 TO 50)

Units ☐mEq/L ☐mmol/L

Please document the values associated with the 'worst' blood gas analysis in the 6 hours prior to commencement of mechanical ventilation. 'Worst' is defined as the blood gas with the lowest PaO2/FiO2 ratio.

□ Not available

2.10 ARTERIAL Base excess IN THE 6 HOURS BEFORE START OF MV _______________ mmol/L (ONLY NUMBERS FROM -50 TO +50)

Please document the values associated with the 'worst' blood gas analysis in the 6 hours prior to commencement of mechanical ventilation. 'Worst' is defined as the blood gas with the lowest PaO2/FiO2 ratio.

□ Not available

2.11 Lactate IN THE 6 HOURS BEFORE START OF MV _______________ mmol/L

Please document the values associated with the 'worst' blood gas analysis in the 6 hours prior to commencement of mechanical ventilation. 'Worst' is defined as the blood gas with the lowest PaO2/FiO2 ratio.

□ Not available

2.12 USE OF CONTINUOUS RENAL REPLACEMENT THERAPY BEFORE START OF MV

- ☐ Yes
- ☐ No

2.13 USE OF VASOACTIVE DRUGS BEFORE START OF MV

- ☐ Yes
- ☐ No

2.14 USE OF CARDIAC ASSIST DEVICES BEFORE START OF MV

- ☐ Yes
- ☐ No

2.15 ANTIBIOTICs BEFORE START OF MV

- ☐ Amikacin
- ☐ Amoxicillin
- ☐ Amoxicillin + Clavulanate
- ☐ Ampicillin
- ☐ Ampicillin + Sulbactam
- ☐ Atovaquone
- ☐ Azithromycin
- ☐ Aztreonam
- ☐ Bacampicillin
- ☐ Bacitracin
- ☐ Capreomycin
- ☐ Carbenicillin indanyl sodium
- ☐ Cefaclor
- ☐ Cefadroxil
- ☐ Cefamandole
- ☐ Cefazolin
- ☐ Cefdinir
- ☐ Cefditoren
- ☐ Cefepime
- ☐ Cefixime
- ☐ Cefmetazole
- ☐ Cefonicid
- ☐ Cefoperazone
- ☐ Cefotaxime
- ☐ Cefotetan
- ☐ Cefoxitin
- ☐ Cefpodoxime Proxetil
- ☐ Cefprozil
- ☐ Ceftaroline
- ☐ Ceftazidime
- ☐ Ceftazidime/Avibactam
- ☐ Ceftibuten
- ☐ Ceftizoxime
- ☐ Ceftobiprole
- ☐ Ceftolozane/Tazobactam
- ☐ Ceftriaxone
- ☐ Cefuroxime
- ☐ Cephalexin
- ☐ Cephalothin
- ☐ Cephapirin
- ☐ Cephradine
- ☐ Chloramphenicol
- ☐ Cinoxacin
- ☐ Ciprofloxacin
- ☐ Clarithromycin
- ☐ Clindamycin
- ☐ Cloxacillin
- ☐ Colistimethate
- ☐ Cycloserine
- ☐ Daptomycin
- ☐ Demeclocycline
- ☐ Dicloxacillin
- ☐ Dirithromycin
- ☐ Doripenem
- ☐ Doxycycline
- ☐ Enoxacin
- ☐ Ertapenem
- ☐ Erythromycin
- ☐ Fosfomycin
- ☐ Gatifloxacin
- ☐ Gemifloxacin
- ☐ Gentamicin
- ☐ Grepafloxacin
- ☐ Imipenem/Cilastatin
- ☐ Imiquimod
- ☐ Kanamycin
- ☐ Levofloxacin
- ☐ Lincomycin
- ☐ Linezolid
- ☐ Lomefloxacin
- ☐ Loracarbef
- ☐ Mafenide
- ☐ Meropenem
- ☐ Methenamine hippurate
- ☐ Methicillin
- ☐ Metronidazole
- ☐ Mezlocillin
- ☐ Minocycline
- ☐ Moxifloxacin
- ☐ Mupirocin
- ☐ Nafcillin
- ☐ Nalidixic Acid
- ☐ Neomycin
- ☐ Netilmicin
- ☐ Nitrofurantoin
- ☐ Nitrofurazone
- ☐ Norfloxacin
- ☐ Novobiocin
- ☐ Ofloxacin

- ☐ Oxacillin
- ☐ Oxytetracycline
- ☐ Penicillin
- ☐ Piperacillin
- ☐ Piperacillin + Tazobactam
- ☐ Podofilox
- ☐ Polymyxin B
- ☐ Quinupristin + Dalfopristin
- ☐ Retapamulin
- ☐ Rifapentine
- ☐ Rifaximin
- ☐ Saturated Solution of Potassium Iodide (SSKI)
- ☐ Sparfloxacin
- ☐ Spectinomycin
- ☐ Streptomycin
- ☐ Sulfadiazine
- ☐ Sulfamethoxazole
- ☐ Sulfisoxazole
- ☐ Sulphur, precipitated in petrolatum
- ☐ TCA (trichloroacetic acid), BCA (bichloroacetic acid).
- ☐ Teicoplanin
- ☐ Telavancin
- ☐ Telithromycin
- ☐ Terbinafine
- ☐ Tetracycline
- ☐ Ticarcillin
- ☐ Ticarcillin + Clavulanic Acid
- ☐ Tigecycline
- ☐ Tobramycin
- ☐ Trimethoprim
- ☐ Trimethoprim + Sulfamethoxazole
- ☐ Trovafloxacin
- ☐ Vancomycin

CORE CASE RECORD FORM (EOT Start ECMO)

3. UPON COMMENCMENT OF ECMO.
Importantly, this module will be active only when you click 'YES' in the field '1.18 ECLS' of the ISARIC form.

3.1 DATE OF START OF ECMO: ___/ ___/ ___ (DD/MM/YY)

3.2 Is this patient enrolled in the EXCEL study? (Australian sites only)

- ☐ Yes
- ☐ No

3.3 If Yes, what is the patient's EXCEL study number___________________________

3.4 Is this patient enrolled in the ELSO Registry?

- ☐ Yes
- ☐ No

3.5 If yes, what is the patient's ELSO Registry number: _______________________

3.6 LOCATION OF ECMO CANNULATION:

- ☐ Same Hospital
- ☐ Other Hospital, then patient was retrieved and transferred

3.7 Type and Manufacturer of centrifugal blood pump driven circuit: __________ (TEXT)

3.8 Type and Manufacturer of low-resistance oxygenator: __________ (TEXT)

3.9 TYPE OF ECMO:

- ☐ Venous-venous
- ☐ Venous-arterial

3.10 DRAINAGE CANNULA INSERTION SITE:

- ☐ Left femoral vein
- ☐ Left internal jugular vein
- ☐ Right femoral vein
- ☐ Right internal jugular vein

3.10a DRAINAGE CANNULA SIZE recorded

- ☐ Yes
- ☐ No

3.10b DRAINAGE CANNULA SIZE

__________ Fr (ONLY NUMBERS, BETWEEN 5 and 30)

3.11 RETURN CANNULA INSERTION SITE:

- ☐ Left femoral vein
- ☐ Left internal jugular vein
- ☐ Right femoral vein
- ☐ Right internal jugular vein
- ☐ Left femoral artery
- ☐ Right femoral artery

3.11a RETURN CANNULA SIZE recorded

- ☐ Yes
- ☐ No

3.11b RETURN CANNULA SIZE

___________ Fr (ONLY NUMBERS, BETWEEN 5 and 30)

3.12 CARDIAC ARREST BEFORE START OF ECMO

- ☐ Yes
- ☐ No

3.13 USE OF PRONE POSITION BEFORE START OF ECMO:

- ☐ Yes
- ☐ No

3.14 USE OF NEUROMUSCULAR BLOCKADE BEFORE START OF ECMO:

- ☐ Yes
- ☐ No

3.15 USE OF RECRUITMENT MANOEUVRES BEFORE START OF ECMO:

- ☐ Yes
- ☐ No

3.16 USE OF INHALED NITRIC OXIDE BEFORE START OF ECMO:

- ☐ Yes
- ☐ No

3.17 USE OF BICARBONATE BEFORE START OF ECMO

- ☐ Yes
- ☐ No

3.18 VENTILATORY MODE BEFORE START OF ECMO:

- ☐ Synchronized Intermittent Mandatory Ventilation – Volume-Controlled (SIMV-V)
- ☐ Synchronized Intermittent Mandatory Ventilation – Pressure-Controlled (SIMV-P)
- ☐ Volume Controlled Ventilation

- ☐ Pressure Controlled Ventilation
- ☐ Pressure Regulated Volume Control (PRVC)
- ☐ Airway Pressure Release Ventilation (APRV)
- ☐ Pressure Support Ventilation (PSV)
- ☐ Volume Support Ventilation (VSV)
- ☐ High Frequency Oscillatory (HFO)
- ☐ Bilevel Positive Airway Pressure (BiPAP)
- ☐ Continuous Positive Airway Pressure (CPAP)
- ☐ Proportional Assist Ventilation (PAV)
- ☐ Neurally Adjusted Ventilatory Assist (NAVA)
- ☐ Other: __________ (TEXT)

MECHANICAL VENTILATION & BLOOD GAS ANALYSIS (Qs 3.19 - 3.30) – Please document the 'worst' value in the 6 hours before the commencement of ECMO. 'Worst' means the values associated with the arterial blood gas with the lowest PaO2/FiO2 ratio. Please report ventilatory settings associated with the worst arterial blood gas.

3.19 INSPIRATORY FRACTION OF OXYGEN IN THE 6 HOURS BEFORE START OF ECMO: __________ (ONLY NUMBERS, BETWEEN 21 and 100)

Please document the values associated with the 'worst' blood gas analysis in the 6 hours prior to commencement of ECMO. 'Worst' is defined as the blood gas with the lowest PaO2/FiO2 ratio.

☐ Not available

3.20 RESPIRATORY RATE IN THE 6 HOURS BEFORE START OF ECMO (breaths/min): __________ (ONLY NUMBERS, BETWEEN 2 and 60)

Please document the values associated with the 'worst' blood gas analysis in the 6 hours prior to commencement of ECMO. 'Worst' is defined as the blood gas with the lowest PaO2/FiO2 ratio.

☐ Not available

3.21 TIDAL VOLUME (ml/Kg of Ideal Body Weight): __________ (ONLY NUMBERS, BETWEEN 1 and 14)

Please document the values associated with the 'worst' blood gas analysis in the 6 hours prior to commencement of ECMO. 'Worst' is defined as the blood gas with the lowest PaO2/FiO2 ratio.

Ideal Body Weight formula:

Male patients: 50 + (0.91 x [height in cm – 152.4])

Female patients: 45.5 + (0.91 x {height in cm – 152.4])

☐ Not available

3.22 POSITIVE END EXPIRATORY PRESSURE IN THE 6 HOURS BEFORE START OF ECMO (cmH2O): __________ (ONLY NUMBERS, BETWEEN 0 and 25)

Please document the values associated with the 'worst' blood gas analysis in the 6 hours prior to commencement of ECMO. 'Worst' is defined as the blood gas with the lowest PaO2/FiO2 ratio.

□ Not available

3.23 PEAK AIRWAY PRESSURE IN THE 6 HOURS BEFORE START OF ECMO (cmH2O): __________ (ONLY NUMBERS, BETWEEN 0 and 85)

Please document the values associated with the 'worst' blood gas analysis in the 6 hours prior to commencement of ECMO. 'Worst' is defined as the blood gas with the lowest PaO2/FiO2 ratio.

□ Not available

3.24 AIRWAY PLATEAU PRESSURE IN THE 6 HOURS BEFORE START OF ECMO (cmH2O): __________ (ONLY NUMBERS, BETWEEN 0 and 50)

Please document the values associated with the 'worst' blood gas analysis in the 6 hours prior to commencement of ECMO. 'Worst' is defined as the blood gas with the lowest PaO2/FiO2 ratio.

□ Not available

3.25 ARTERIAL pH IN THE 6 HOURS BEFORE START OF ECMO: __________ (ONLY NUMBERS FROM 6.500 TO 7.600)

Please document the values associated with the 'worst' blood gas analysis in the 6 hours prior to commencement of ECMO. 'Worst' is defined as the blood gas with the lowest PaO2/FiO2 ratio.

□ Not available

3.26 ARTERIAL PARTIAL PRESSURE OF OXYGEN IN THE 6 HOURS BEFORE START OF ECMO: __________ (ONLY NUMBERS FROM 20 TO 500)

Please document the values associated with the 'worst' blood gas analysis in the 6 hours prior to commencement of ECMO. 'Worst' is defined as the blood gas with the lowest PaO2/FiO2 ratio.

Units: ☐mmHg ☐kPa

□ Not available

3.27 ARTERIAL PARTIAL PRESSURE OF CARBON DIOXIDE IN THE 6 HOURS BEFORE START OF ECMO: __________ (ONLY NUMBERS FROM 10 TO 150)

Please document the values associated with the 'worst' blood gas analysis in the 6 hours prior to commencement of ECMO. 'Worst' is defined as the blood gas with the lowest PaO2/FiO2 ratio.

Units: ☐mmHg ☐kPa

□ Not available

3.28 ARTERIAL $HCO3^-$ IN THE 6 HOURS BEFORE START OF ECMO: ________________ (ONLY NUMBERS FROM 1 TO 50)

Please document the values associated with the 'worst' blood gas analysis in the 6 hours prior to commencement of ECMO. 'Worst' is defined as the blood gas with the lowest PaO2/FiO2 ratio.

Units: ☐ mEq/L ☐ mmol/L

☐ Not available

3.29 ARTERIAL Base excess IN THE 6 HOURS BEFORE START OF ECMO: ______________mmol/L (ONLY NUMBERS FROM -50 TO +50)

Please document the values associated with the 'worst' blood gas analysis in the 6 hours prior to commencement of ECMO. 'Worst' is defined as the blood gas with the lowest PaO2/FiO2 ratio.

☐ Not available

3.30 Lactate IN THE 6 HOURS BEFORE START OF ECMO: _______________ mmol/L

Please document the values associated with the 'worst' blood gas analysis in the 6 hours prior to commencement of ECMO. 'Worst' is defined as the blood gas with the lowest PaO2/FiO2 ratio.

☐ Not available

3.31 USE OF CONTINUOUS RENAL REPLACEMENT THERAPY BEFORE START OF ECMO:

- ☐ Yes
- ☐ No

3.32 USE OF VASOACTIVE DRUGS BEFORE START OF ECMO:

- ☐ Yes
- ☐ No

3.33 USE OF CARDIAC ASSIST DEVICE BEFORE START OF ECMO:

- ☐ Yes
- ☐ No

3.34 USE OF ANTIBIOTICS BEFORE START OF ECMO:

- ☐ Yes
- ☐ No

3.35 ANTIBIOTICs BEFORE START OF ECMO:

☐ Amikacin	☐ Amoxicillin + Clavulanate	☐ Ampicillin + Sulbactam
☐ Amoxicillin	☐ Ampicillin	☐ Atovaquone

- ☐ Azithromycin
- ☐ Aztreonam
- ☐ Bacampicillin
- ☐ Bacitracin
- ☐ Capreomycin
- ☐ Carbenicillin indanyl sodium
- ☐ Cefaclor
- ☐ Cefadroxil
- ☐ Cefamandole
- ☐ Cefazolin
- ☐ Cefdinir
- ☐ Cefditoren
- ☐ Cefepime
- ☐ Cefixime
- ☐ Cefmetazole
- ☐ Cefonicid
- ☐ Cefoperazone
- ☐ Cefotaxime
- ☐ Cefotetan
- ☐ Cefoxitin
- ☐ Cefpodoxime Proxetil
- ☐ Cefprozil
- ☐ Ceftaroline
- ☐ Ceftazidime
- ☐ Ceftazidime/Avibactam
- ☐ Ceftibuten
- ☐ Ceftizoxime
- ☐ Ceftobiprole
- ☐ Ceftolozane/Tazobactam
- ☐ Ceftriaxone
- ☐ Cefuroxime
- ☐ Cephalexin
- ☐ Cephalothin
- ☐ Cephapirin
- ☐ Cephradine
- ☐ Chloramphenicol
- ☐ Cinoxacin
- ☐ Ciprofloxacin
- ☐ Clarithromycin
- ☐ Clindamycin
- ☐ Cloxacillin
- ☐ Colistimethate
- ☐ Cycloserine
- ☐ Daptomycin
- ☐ Demeclocycline
- ☐ Dicloxacillin
- ☐ Dirithromycin
- ☐ Doripenem
- ☐ Doxycycline
- ☐ Enoxacin
- ☐ Ertapenem
- ☐ Erythromycin
- ☐ Fosfomycin
- ☐ Gatifloxacin
- ☐ Gemifloxacin
- ☐ Gentamicin
- ☐ Grepafloxacin
- ☐ Imipenem/Cilastatin
- ☐ Imiquimod
- ☐ Kanamycin
- ☐ Levofloxacin
- ☐ Lincomycin
- ☐ Linezolid
- ☐ Lomefloxacin
- ☐ Loracarbef
- ☐ Mafenide
- ☐ Meropenem
- ☐ Methenamine hippurate
- ☐ Methicillin
- ☐ Metronidazole
- ☐ Mezlocillin
- ☐ Minocycline
- ☐ Moxifloxacin
- ☐ Mupirocin
- ☐ Nafcillin
- ☐ Nalidixic Acid
- ☐ Neomycin
- ☐ Netilmicin
- ☐ Nitrofurantoin
- ☐ Nitrofurazone
- ☐ Norfloxacin
- ☐ Novobiocin
- ☐ Ofloxacin
- ☐ Oxacillin
- ☐ Oxytetracycline
- ☐ Penicillin
- ☐ Piperacillin
- ☐ Piperacillin + Tazobactam
- ☐ Podofilox
- ☐ Polymyxin B
- ☐ Quinupristin + Dalfopristin
- ☐ Retapamulin
- ☐ Rifapentine
- ☐ Rifaximin
- ☐ Saturated Solution of Potassium Iodide (SSKI)
- ☐ Sparfloxacin
- ☐ Spectinomycin
- ☐ Streptomycin
- ☐ Sulfadiazine
- ☐ Sulfamethoxazole
- ☐ Sulfisoxazole
- ☐ Sulphur, precipitated in petrolatum
- ☐ TCA (trichloroacetic acid), BCA (bichloroacetic acid).
- ☐ Teicoplanin
- ☐ Telavancin
- ☐ Telithromycin
- ☐ Terbinafine
- ☐ Tetracycline
- ☐ Ticarcillin
- ☐ Ticarcillin + Clavulanic Acid
- ☐ Tigecycline
- ☐ Tobramycin
- ☐ Trimethoprim
- ☐ Trimethoprim + Sulfamethoxazole
- ☐ Trovafloxacin
- ☐ Vancomycin

3.36 CHEST X-RAY WITHIN 24h PRE or POST- ECMO CANNULATION:

- ☐ Yes
- ☐ No

3.36a If yes to 3.36, Number of CHEST X-RAY quadrants with infiltrates:

- ☐ 0
- ☐ 1
- ☐ 2
- ☐ 3
- ☐ 4
- ☐ Unknown

4. DAILY CASE RECORD FORM (EOT Daily)

Option 1: 'FULL' daily data

Complete the daily form every day of mechanical ventilation (ie. from mechanical ventilation commencement (intubation) to discontinuation of mechanical ventilation (extubation)). **Please commence this data the day after the patient is intubated.**

Please collect all daily data retrospectively, at least 24h after the day of assessment, since the worst parameters of the 24-h period of assessment need to be identified.

Option 2: 'BASIC' data

Complete this daily form:

1. Mechanical ventilation commencement
2. ECMO commencement
3. Four (4) days after ICU admission (only if the patient is mechanically ventilated or ECMO at that time)
4. Mechanical ventilation discontinuation.
5. ECMO discontinuation

Please collect all daily data retrospectively, at least 24h after the day of assessment, since the worst parameters of the 24-h period of assessment need to be identified.

Importantly, parameters related to mechanical ventilation or ECMO will be active only when you click 'YES' in the field '1.17 Invasive ventilation' or when you click 'YES' in the field '1.18 ECLS', respectively, of the ISARIC "Daily Form".

4.1 DATE: ________________________________

4.2 PATIENT POSITION:

'Full' daily data collection: Patient position applied most predominantly ***in the last 24 hours***

'Basic' daily data collection: Patient position applied most predominantly ***since the last EOT Daily form***

- *If this is the* ***'Four days after ICU admission'*** *timepoint, please collect the position applied most predominantly in the last 24 hours.*

☐ Supine
☐ Prone

4.3 HIGHEST ECMO FLOW RATE IN THE LAST 24h (L/min): __________

4.4 HIGHEST ECMO GAS FLOW RATE IN THE LAST 24h (L/min): __________

4.5 ECMO CIRCUIT CHANGE:

'Full' daily data collection: Circuit change ***in the last 24 hours***

'Basic' daily data collection: Circuit change ***since the last EOT Daily form***

- *If this is the* ***'Four days after ICU admission'*** *timepoint, please answer with reference to the last 24 hours.*

☐ Yes
☐ No

4.6 USE OF NEUROMUSCULAR BLOCKADE:

'Full' daily data collection: Neuromuscular blockade ***in the last 24 hours***

'Basic' daily data collection: Neuromuscular blockade ***since the last EOT Daily form***

- *If this is the* ***'Four days after ICU admission'*** *timepoint, please answer with reference to the last 24 hours.*

☐ Yes
☐ No

4.7 USE OF RECRUITMENT MANOEUVRES:

'Full' daily data collection: Recruitment manoeuvres ***in the last 24 hours***

'Basic' daily data collection: Recruitment manoeuvres ***since the last EOT Daily form***

- *If this is the* ***'Four days after ICU admission'*** *timepoint, please answer with reference to the last 24 hours.*

☐ Yes
☐ No

4.8 USE OF INHALED NITRIC OXIDE:

'Full' daily data collection: Inhaled nitric oxide ***in the last 24 hours***

'Basic' daily data collection: Inhaled nitric oxide ***since the last EOT Daily form***

- *If this is the* ***'Four days after ICU admission'*** *timepoint, please answer with reference to the last 24 hours.*

☐ Yes
☐ No

4.9 MOST FREQUENT VENTILATORY MODE IN THE LAST 24h:

☐ Synchronized Intermittent Mandatory Ventilation – Volume-Controlled (SIMV-V)
☐ Synchronized Intermittent Mandatory Ventilation – Pressure-Controlled (SIMV-P)
☐ Volume Controlled Ventilation
☐ Pressure Controlled Ventilation
☐ Pressure Regulated Volume Control (PRVC)
☐ Airway Pressure Release Ventilation (APRV)
☐ Pressure Support Ventilation (PSV)
☐ Volume Support Ventilation (VSV)
☐ High Frequency Oscillatory (HFO)
☐ Bylevel Positive Airway Pressure (BiPAP)
☐ Continuous Positive Airway Pressure (CPAP)
☐ Proportional Assist Ventilation (PAV)
☐ Neurally Adjusted Ventilatory Assist (NAVA)
☐ Other: __________ (TEXT)

MECHANICAL VENTILATION & BLOOD GAS ANALYSIS (Qs 4.10 – 4.21) – Please document the 'worst' value in the last 24 hours. 'Worst' means the values associated with the arterial blood gas with the lowest PaO2/FiO2 ratio. Please report ventilatory settings associated with the worst arterial blood gas.

4.10 INSPIRATORY FRACTION OF OXYGEN IN THE LAST 24h: __________ (ONLY NUMBERS, BETWEEN 21 and 100)

Please document the values associated with the 'worst' blood gas analysis in the last 24 hours. 'Worst' is defined as the blood gas with the lowest PaO2/FiO2 ratio.

□ Not available

4.11 RESPIRATORY RATE IN THE LAST 24h (breaths/min): __________ (ONLY NUMBERS, BETWEEN 2 and 60)

Please document the values associated with the 'worst' blood gas analysis in the last 24 hours. 'Worst' is defined as the blood gas with the lowest PaO2/FiO2 ratio

□ Not available

4.12 TIDAL VOLUME IN THE LAST 24h (ml/Kg of Ideal Body Weight): __________ (ONLY NUMBERS, BETWEEN 1 and 14)

Please document the values associated with the 'worst' blood gas analysis in the last 24 hours. 'Worst' is defined as the blood gas with the lowest PaO2/FiO2 ratio.

Ideal Body Weight formula:

Male patients: 50 + (0.91 x [height in cm – 152.4])

Female patients: 45.5 + (0.91 x {height in cm – 152.4])

□ Not available

4.13 POSITIVE END EXPIRATORY PRESSURE IN THE LAST 24h (cmH2O): __________ (ONLY NUMBERS, BETWEEN 0 and 25)

Please document the values associated with the 'worst' blood gas analysis in the last 24 hours. 'Worst' is defined as the blood gas with the lowest PaO2/FiO2 ratio.

□ Not available

4.14 AIRWAY PLATEAU PRESSURE IN THE LAST 24h (cmH2O): __________ (ONLY NUMBERS, BETWEEN 0 and 50)

Please document the values associated with the 'worst' blood gas analysis in the last 24 hours. 'Worst' is defined as the blood gas with the lowest PaO2/FiO2 ratio.

□ Not available

4.15 ARTERIAL pH IN THE LAST 24h: __________ (ONLY NUMBERS FROM 6.500 TO 7.600)

Please document the values associated with the 'worst' blood gas analysis in the last 24 hours. 'Worst' is defined as the blood gas with the lowest PaO2/FiO2 ratio.

□ Not available

4.16 ARTERIAL PARTIAL PRESSURE OF OXYGEN IN THE LAST 24h: ____________(ONLY NUMBERS FROM 20 TO 500)

Units: ☐mmHg ☐kPa

Please document the values associated with the 'worst' blood gas analysis in the last 24 hours. 'Worst' is defined as the blood gas with the lowest PaO2/FiO2 ratio.

□ Not available

4.17 ARTERIAL PARTIAL PRESSURE OF CARBON DIOXIDE IN THE LAST 24h: __________ (ONLY NUMBERS FROM 10 TO 100)

Units: ☐mmHg ☐kPa

Please document the values associated with the 'worst' blood gas analysis in the last 24 hours. 'Worst' is defined as the blood gas with the lowest PaO2/FiO2 ratio.

□ Not available

4.18 ARTERIAL HCO_3^- IN THE LAST 24h:________________ (ONLY NUMBERS FROM 1 TO 50)

Units: ☐ mEq/L ☐ mmol/L

Please document the values associated with the 'worst' blood gas analysis in the last 24 hours. 'Worst' is defined as the blood gas with the lowest PaO2/FiO2 ratio.

□ Not available

4.19 ARTERIAL Base excess IN THE LAST 24h: _______________ mmol/L (ONLY NUMBERS FROM -50 TO +50)

Please document the values associated with the 'worst' blood gas analysis in the last 24 hours. 'Worst' is defined as the blood gas with the lowest PaO2/FiO2 ratio.

4.20 Lactate IN THE LAST 24h: _______________ mmol/L

Please document the values associated with the 'worst' blood gas analysis in the last 24 hours. 'Worst' is defined as the blood gas with the lowest PaO2/FiO2 ratio.

If this data has already been entered in the 'Daily Case Report Form – Laboratory Results' section of the ISARIC CRF, please DO NOT re-enter the data here. Please leave '4.20 Lactate' blank.

4.21 CREATININE IN THE LAST 24h : __________

Units: ☐ mg/dL ☐ µmol/L

Please document the values associated with the 'worst' blood gas analysis in the last 24 hours. 'Worst' is defined as the blood gas with the lowest PaO2/FiO2 ratio.

☐ Not available

If this data has already been entered in the 'Daily Case Report Form – Laboratory Results' section of the ISARIC CRF, please DO NOT re-enter the data here. Please leave '4.21 Creatinine' blank.

4.22 USE OF CONTINUOUS RENAL REPLACEMENT THERAPY (CRRT):

*'Full' daily data collection: CRRT **in the last 24 hours***

*'Basic' daily data collection: CRRT **since the last EOT Daily form***

- *If this is the **'Four days after ICU admission'** timepoint, please answer with reference to the last 24 hours.*

☐ Yes
☐ No

4.23 USE OF VASOACTIVE DRUGS IN THE LAST 24h:

☐ Yes
☐ No

4.24 TYPE OF VASOACTIVE DRUG 1:

☐ Dobutamine ☐
☐ Dopamine ☐
☐ Enoximone ☐
☐ Epinephrine: YES ☐ NO ☐
☐ Esmolol ☐
☐ Levosimendan ☐
☐ Metaraminol ☐
☐ Metoprolol ☐
☐ Milrinone ☐
☐ Nicardipine ☐
☐ Nitroglycerin ☐
☐ Nitroprusside ☐
☐ Norepinephrine: YES ☐ NO ☐
☐ Phenylephrine ☐
☐ Tolazoline ☐
☐ Vasopressin ☐

4.25 HIGHEST DOSE OF VASOACTIVE DRUG 1 IN THE LAST 24h (mcg/Kg/min): ___________

4.26 TYPE OF VASOACTIVE DRUG 2:

☐ Dobutamine ☐
☐ Dopamine ☐
☐ Enoximone ☐
☐ Epinephrine: YES ☐ NO ☐
☐ Esmolol ☐
☐ Levosimendan ☐
☐ Metaraminol ☐
☐ Metoprolol ☐
☐ Milrinone ☐

- Nicardipine ☐
- Nitroglycerin ☐
- Nitroprusside ☐
- Norepinephrine: YES ☐ NO ☐
- Phenylephrine ☐
- Tolazoline ☐
- Vasopressin ☐

4.27 HIGHEST DOSE OF VASOACTIVE DRUG 2 IN THE LAST 24h (mcg/Kg/min): __________

4.28 TYPE OF VASOACTIVE DRUG 3:

- Dobutamine ☐
- Dopamine ☐
- Enoximone ☐
- Epinephrine: YES ☐ NO ☐
- Esmolol ☐
- Levosimendan ☐
- Metaraminol ☐
- Metoprolol ☐
- Milrinone ☐
- Nicardipine ☐
- Nitroglycerin ☐
- Nitroprusside ☐
- Norepinephrine: YES ☐ NO ☐
- Phenylephrine ☐
- Tolazoline ☐
- Vasopressin ☐

4.29 HIGHEST DOSE OF VASOACTIVE DRUG 3 IN THE LAST 24h (mcg/Kg/min): __________

4.30 USE OF CARDIAC ASSIST DEVICES:

*'Full' daily data collection: Cardiac assist device use **in the last 24 hours***

*'Basic' daily data collection: Cardiac assist device use **since the last EOT Daily form***

- *If this is the '**Four days after ICU admission'** timepoint, please answer with reference to the last 24 hours.*

- Yes
- No

4.31 USE OF ANTIBIOTICS:

*<u>'Full' daily data collection</u>: Antibiotics administered **in the last 24 hours***

*<u>'Basic' daily data collection</u>: Antibiotics administered **since the last EOT Daily form***

- *If this is the **'Four days after ICU admission'** timepoint, please answer with reference to the last 24 hours.*

- Yes
- No

ANTIBIOTICs:

- ☐ Amikacin
- ☐ Amoxicillin
- ☐ Amoxicillin + Clavulanate
- ☐ Ampicillin
- ☐ Ampicillin + Sulbactam
- ☐ Atovaquone
- ☐ Azithromycin
- ☐ Aztreonam
- ☐ Bacampicillin
- ☐ Bacitracin
- ☐ Capreomycin
- ☐ Carbenicillin indanyl sodium
- ☐ Cefaclor
- ☐ Cefadroxil
- ☐ Cefamandole
- ☐ Cefazolin
- ☐ Cefdinir
- ☐ Cefditoren
- ☐ Cefepime
- ☐ Cefixime
- ☐ Cefmetazole
- ☐ Cefonicid
- ☐ Cefoperazone
- ☐ Cefotaxime
- ☐ Cefotetan
- ☐ Cefoxitin
- ☐ Cefpodoxime Proxetil
- ☐ Cefprozil
- ☐ Ceftaroline
- ☐ Ceftazidime
- ☐ Ceftazidime/Avibactam
- ☐ Ceftibuten
- ☐ Ceftizoxime
- ☐ Ceftobiprole
- ☐ Ceftolozane/Tazobactam
- ☐ Ceftriaxone
- ☐ Cefuroxime
- ☐ Cephalexin
- ☐ Cephalothin
- ☐ Cephapirin
- ☐ Cephradine
- ☐ Chloramphenicol
- ☐ Cinoxacin
- ☐ Ciprofloxacin
- ☐ Clarithromycin
- ☐ Clindamycin
- ☐ Cloxacillin
- ☐ Colistimethate
- ☐ Cycloserine
- ☐ Daptomycin
- ☐ Demeclocycline
- ☐ Dicloxacillin
- ☐ Dirithromycin
- ☐ Doripenem
- ☐ Doxycycline
- ☐ Enoxacin
- ☐ Ertapenem
- ☐ Erythromycin
- ☐ Fosfomycin
- ☐ Gatifloxacin
- ☐ Gemifloxacin
- ☐ Gentamicin
- ☐ Grepafloxacin
- ☐ Imipenem/Cilastatin
- ☐ Imiquimod
- ☐ Kanamycin
- ☐ Levofloxacin
- ☐ Lincomycin
- ☐ Linezolid
- ☐ Lomefloxacin
- ☐ Loracarbef
- ☐ Mafenide
- ☐ Meropenem
- ☐ Methenamine hippurate
- ☐ Methicillin
- ☐ Metronidazole
- ☐ Mezlocillin
- ☐ Minocycline
- ☐ Moxifloxacin
- ☐ Mupirocin
- ☐ Nafcillin
- ☐ Nalidixic Acid
- ☐ Neomycin
- ☐ Netilmicin
- ☐ Nitrofurantoin
- ☐ Nitrofurazone
- ☐ Norfloxacin
- ☐ Novobiocin
- ☐ Ofloxacin
- ☐ Oxacillin
- ☐ Oxytetracycline
- ☐ Penicillin
- ☐ Piperacillin
- ☐ Piperacillin + Tazobactam
- ☐ Podofilox
- ☐ Polymyxin B
- ☐ Quinupristin + Dalfopristin
- ☐ Retapamulin
- ☐ Rifapentine
- ☐ Rifaximin
- ☐ Saturated Solution of Potassium Iodide (SSKI)
- ☐ Sparfloxacin
- ☐ Spectinomycin
- ☐ Streptomycin
- ☐ Sulfadiazine
- ☐ Sulfamethoxazole
- ☐ Sulfisoxazole
- ☐ Sulphur, precipitated in petrolatum
- ☐ TCA (trichloroacetic acid), BCA (bichloroacetic acid).
- ☐ Teicoplanin
- ☐ Telavancin
- ☐ Telithromycin
- ☐ Terbinafine
- ☐ Tetracycline
- ☐ Ticarcillin
- ☐ Ticarcillin + Clavulanic Acid
- ☐ Tigecycline
- ☐ Tobramycin
- ☐ Trimethoprim
- ☐ Trimethoprim + Sulfamethoxazole
- ☐ Trovafloxacin
- ☐ Vancomycin

4.32 Haemoglobin IN THE LAST 24h **g/dL** ___________________

◻ Not available

If this data has already been entered in the 'Daily Case Report Form – Laboratory Results' section of the ISARIC CRF, please DO NOT re-enter the data here. Please leave '4.32 Haemoglobin' blank.

4.33 White Blood Cells IN THE LAST 24h

◻ Not available

If this data has already been entered in the 'Daily Case Report Form – Laboratory Results' section of the ISARIC CRF, please DO NOT re-enter the data here. Please leave '4.33 White Blood Cells' blank.

4.34 White Blood Cells Unit

- ☐ X 10^9/L
- ☐ X 10^3/microL

4.35 AST/SGOT IN THE LAST 24h **U/L** ___________________

◻ Not available

If this data has already been entered in the 'Daily Case Report Form – Laboratory Results' section of the ISARIC CRF, please DO NOT re-enter the data here. Please leave '4.34 AST' blank.

4.36 ALT/SGPT IN THE LAST 24h **U/L** ___________________

◻ Not available

If this data has already been entered in the 'Daily Case Report Form – Laboratory Results' section of the ISARIC CRF, please DO NOT re-enter the data here. Please leave '4.36 ALT' blank.

4.37 ANTICOAGULANTS:

'Full' daily data collection: Anticoagulants administered ***in the last 24 hours***

'Basic' daily data collection: Anticoagulants administered ***since the last EOT Daily form***

- *If this is the* ***'Four days after ICU admission'*** *timepoint, please answer with reference to the last 24 hours.*
- ☐ Yes
- ☐ No

4.38 TYPE OF ANTICOAGULANTS:

'Full' daily data collection: Anticoagulants administered ***in the last 24 hours***

'Basic' daily data collection: Anticoagulants administered ***since the last EOT Daily form***

- *If this is the '****Four days after ICU admission' timepoint,*** *please answer with reference to the last 24 hours.*
- ☐ Continuous infusion of unfractionated heparin
- ☐ Subcutaneous unfractionated heparin only
- ☐ Low molecular heparin

- ☐ Danaparoid Lepirudin
- ☐ Argatroban
- ☐ Hirulog and bivalirudin
- ☐ Desirudin
- ☐ Nafamostat Mesilate
- ☐ Other

4.39 TRANSFUSED PACKED RED BLOOD CELL (PRBC) CONCENTRATE:

*<u>'Full' daily data collection</u>: PRBCs administered **in the last 24 hours***

*<u>'Basic' daily data collection</u>: PRBCs administered **since the last EOT Daily form***

- *If this is the **'Four days after ICU admission'** timepoint, please answer with reference to the last 24 hours*

- ☐ Yes
- ☐ No

4.40 TRANSFUSED PLATELETS CONCENTRATE:

*<u>'Full' daily data collection</u>: Platelets administered **in the last 24 hours***

*<u>'Basic' daily data collection</u>: Platelets administered **since the last EOT Daily form***

- *If this is the **'Four days after ICU admission'** timepoint, please answer with reference to the last 24 hours*

- ☐ Yes
- ☐ No

4.41 TRANSFUSED FRESH FROZEN PLASMA (FFP):

*<u>'Full' daily data collection</u>: FFP administered **in the last 24 hours***

*<u>'Basic' daily data collection</u>: FFP administered **since the last EOT Daily form***

- *If this is the **'Four days after ICU admission'** timepoint, please answer with reference to the last 24 hours*

- ☐ Yes
- ☐ No

4.42 TRANSFUSED CRYOPRECIPITATES:

*<u>'Full' daily data collection</u>: Cryoprecipitate administered **in the last 24 hours***

*<u>'Basic' daily data collection</u>: Cryoprecipitate administered **since the last EOT Daily form***

- *If this is the **'Four days after ICU admission'** timepoint, please answer with reference to the last 24 hours*

- ☐ Yes
- ☐ No

4.43 INFECTION COMPLICATION 1:

*<u>'Full' daily data collection:</u> Infectious complications diagnosed **in the last 24 hours***

*<u>'Basic' daily data collection:</u> Infectious complications diagnosed **since the last EOT Daily form***

- *If this is the **'Four days after ICU admission'** timepoint, please answer with reference to the last 24 hours*

- ☐ Yes
- ☐ No

4.44 INFECTION COMPLICATION 1 DATE OF DIAGNOSIS:

__ __/ __ __/ __ __ __ __ (DD/MM/YYYY)

4.45 SOURCE OF INFECTIOUS COMPLICATION 1

- ☐ Lungs
- ☐ Gastro-intestinal
- ☐ Genito-urinary
- ☐ Skin and soft tissue
- ☐ Central nervous system
- ☐ Osteoarticular and bone
- ☐ Cardiac
- ☐ Bloodstream
- ☐ Not known

4.46 CAUSATIVE PATHOGEN 1:

- ☐ Acinetobacter baumannii
- ☐ Actinomyces
- ☐ Aeromonas
- ☐ Bacillus anthracis
- ☐ Bacillus species
- ☐ Bacteroides fragilis
- ☐ Bacteroides species
- ☐ Bartonella species
- ☐ Bordetella species
- ☐ Borrelia burgdorferi
- ☐ Borrelia species
- ☐ Brucella Species
- ☐ Burkholderia cepacia
- ☐ Burkholderia mallei
- ☐ Burkholderia pseudomallei
- ☐ Campylobacter and related species
- ☐ Campylobacter jejuni
- ☐ Capnocytophaga canimorsus
- ☐ Chlamydia trachomatis
- ☐ Chlamydophila pneumoniae
- ☐ Chlamydophila psittaci
- ☐ Citrobacter species
- ☐ Clostridium botulinum
- ☐ Clostridium difficile
- ☐ Clostridium species
- ☐ Clostridium tetani (Tetanus)
- ☐ Corynebacterium diphtheriae
- ☐ Coxiella burnetii
- ☐ Ehrlichia species
- ☐ Eikenella corrodens
- ☐ Enterobacter species
- ☐ Enterococcus
- ☐ Erysipelothrix rhusiopathiae
- ☐ Escherichia coli
- ☐ Francisella tularensis
- ☐ Haemophilus ducreyi (Chancroid)
- ☐ Haemophilus influenzae
- ☐ Helicobacter cinaedi and related species
- ☐ Helicobacter pylori
- ☐ Klebsiella granulomatis (Antibiotic Guide)
- ☐ Klebsiella species
- ☐ ESBL Klebsiella pneumoniae
- ☐ Lactobacillus
- ☐ Legionella pneumophila
- ☐ Legionella species
- ☐ Leptospira interrogans
- ☐ Listeria monocytogenes
- ☐ Lymphogranuloma venereum (LGV)
- ☐ Methicillin Resistant Staphylococcus aureus
- ☐ Moraxella catarrhalis
- ☐ Morganella
- ☐ Mycobacterium abscessus
- ☐ Mycobacterium avium-complex (MAC, MAI, non-HIV)
- ☐ Mycobacterium chelonae
- ☐ Mycobacterium fortuitum
- ☐ Mycobacterium gordonae
- ☐ Mycobacterium kansasii
- ☐ Mycobacterium leprae
- ☐ Mycobacterium marinum
- ☐ Mycobacterium scrofulaceum
- ☐ Mycobacterium tuberculosis
- ☐ Mycobacterium ulcerans
- ☐ Mycobacterium xenopi
- ☐ Mycoplasma pneumoniae (Antibiotic Guide)
- ☐ Neisseria gonorrhoeae
- ☐ Neisseria meningitidis
- ☐ Nocardia
- ☐ Other atypical mycobacteria
- ☐ Pasteurella multocida
- ☐ Peptostreptococcus/Peptococcus
- ☐ Plesiomonas
- ☐ Propionibacterium species
- ☐ Proteus species
- ☐ Providencia
- ☐ Pseudomonas aeruginosa
- ☐ Rhodococcus equi

- ☐ Rickettsia rickettsii
- ☐ Rickettsia species
- ☐ Salmonella species
- ☐ Serratia species
- ☐ Shigella dysenteriae
- ☐ Shigella species
- ☐ Staphylococci, coagulase negative
- ☐ Staphylococcus aureus
- ☐ Stenotrophomonas maltophilia
- ☐ Streptobacillus moniliformis
- ☐ Streptococcus pneumoniae
- ☐ Streptococcus pyogenes (Group A)
- ☐ Streptococcus species
- ☐ Treponema pallidum (syphilis)
- ☐ Tropheryma whipplei
- ☐ Vancomycin Resistant Enterococcus species
- ☐ Vancomycin Resistant Staphylococcus aureus
- ☐ Vibrio cholerae
- ☐ Vibrio species (noncholera)
- ☐ Yersinia pestis
- ☐ Yersinia species (non-plague)
- ☐ Absidia
- ☐ Aspergillus
- ☐ Basidiobolomycosis
- ☐ Blastomyces dermatitidis
- ☐ Candida albicans
- ☐ Candida glabrata
- ☐ Candida guilliermondii
- ☐ Candida krusei
- ☐ Candida lusitaniae
- ☐ Candida parapsilosis
- ☐ Candida species
- ☐ Candida tropicalis
- ☐ Chromomycosis
- ☐ Coccidioides immitis
- ☐ Cryptococcus neoformans
- ☐ Cunninghamella
- ☐ Dermatophytes
- ☐ Fusarium
- ☐ Histoplasma capsulatum
- ☐ Mucor
- ☐ Mycetoma
- ☐ Pneumocystis carinii
- ☐ Pneumocystis jirovecii
- ☐ Pseudallescheria boydii
- ☐ Rhizomucor
- ☐ Rhizopus
- ☐ Saksanea
- ☐ Sporothrix schenckii
- ☐ Zygomycetes

4.47 INFECTION COMPLICATION 2:

<u>'Full' daily data collection</u>: Infectious complications diagnosed ***in the last 24 hours***

<u>'Basic' daily data collection</u>: Infectious complications diagnosed ***since the last EOT Daily form***

- *If this is the '**Four days after ICU admission'** timepoint, please answer with reference to the last 24 hours*

  - ☐ Yes
  - ☐ No

4.48 INFECTION COMPLICATION 2 DATE OF DIAGNOSIS:

__ __ / __ __ / __ __ __ __ (DD/MM/YYYY)

4.49 SOURCE OF INFECTIOUS COMPLICATION 2:

- ☐ Lungs
- ☐ Gastro-intestinal
- ☐ Genito-urinary
- ☐ Skin and soft tissue
- ☐ Central nervous system
- ☐ Osteoarticular and bone
- ☐ Cardiac
- ☐ Bloodstream
- ☐ Not known

4.50 CAUSATIVE PATHOGEN 2:

- ☐ Acinetobacter baumannii
- ☐ Actinomyces
- ☐ Aeromonas
- ☐ Bacillus anthracis
- ☐ Bacillus species
- ☐ Bacteroides fragilis
- ☐ Bacteroides species
- ☐ Bartonella species
- ☐ Bordetella species
- ☐ Borrelia burgdorferi
- ☐ Borrelia species
- ☐ Brucella Species
- ☐ Burkholderia cepacia
- ☐ Burkholderia mallei
- ☐ Burkholderia pseudomallei
- ☐ Campylobacter and related species
- ☐ Campylobacter jejuni
- ☐ Capnocytophaga canimorsus
- ☐ Chlamydia trachomatis
- ☐ Chlamydophila pneumoniae

- ☐ Chlamydophila psittaci
- ☐ Citrobacter species
- ☐ Clostridium botulinum
- ☐ Clostridium difficile
- ☐ Clostridium species
- ☐ Clostridium tetani (Tetanus)
- ☐ Corynebacterium diphtheriae
- ☐ Coxiella burnetii
- ☐ Ehrlichia species
- ☐ Eikenella corrodens
- ☐ Enterobacter species
- ☐ Enterococcus
- ☐ Erysipelothrix rhusiopathiae
- ☐ Escherichia coli
- ☐ Francisella tularensis
- ☐ Haemophilus ducreyi (Chancroid)
- ☐ Haemophilus influenzae
- ☐ Helicobacter cinaedi and related species
- ☐ Helicobacter pylori
- ☐ Klebsiella granulomatis (Antibiotic Guide)
- ☐ Klebsiella species
- ☐ ESBL Klebsiella pneumoniae
- ☐ Lactobacillus
- ☐ Legionella pneumophila
- ☐ Legionella species
- ☐ Leptospira interrogans
- ☐ Listeria monocytogenes
- ☐ Lymphogranuloma venereum (LGV)
- ☐ Methicillin Resistant Staphylococcus aureus
- ☐ Moraxella catarrhalis
- ☐ Morganella
- ☐ Mycobacterium abscessus
- ☐ Mycobacterium avium-complex (MAC, MAI, non-HIV)
- ☐ Mycobacterium chelonae
- ☐ Mycobacterium fortuitum
- ☐ Mycobacterium gordonae
- ☐ Mycobacterium kansasii
- ☐ Mycobacterium leprae
- ☐ Mycobacterium marinum
- ☐ Mycobacterium scrofulaceum
- ☐ Mycobacterium tuberculosis
- ☐ Mycobacterium ulcerans
- ☐ Mycobacterium xenopi
- ☐ Mycoplasma pneumoniae (Antibiotic Guide)
- ☐ Neisseria gonorrhoeae
- ☐ Neisseria meningitidis
- ☐ Nocardia
- ☐ Other atypical mycobacteria
- ☐ Pasteurella multocida
- ☐ Peptostreptococcus/Peptococcus
- ☐ Plesiomonas
- ☐ Propionibacterium species
- ☐ Proteus species
- ☐ Providencia
- ☐ Pseudomonas aeruginosa
- ☐ Rhodococcus equi
- ☐ Rickettsia rickettsii
- ☐ Rickettsia species
- ☐ Salmonella species
- ☐ Serratia species
- ☐ Shigella dysenteriae
- ☐ Shigella species
- ☐ Staphylococci, coagulase negative
- ☐ Staphylococcus aureus
- ☐ Stenotrophomonas maltophilia
- ☐ Streptobacillus moniliformis
- ☐ Streptococcus pneumoniae
- ☐ Streptococcus pyogenes (Group A)
- ☐ Streptococcus species
- ☐ Treponema pallidum (syphilis)
- ☐ Tropheryma whipplei
- ☐ Vancomycin Resistant Enterococcus species
- ☐ Vancomycin Resistant Staphylococcus aureus
- ☐ Vibrio cholerae
- ☐ Vibrio species (noncholera)
- ☐ Yersinia pestis
- ☐ Yersinia species (non-plague)
- ☐ Absidia
- ☐ Aspergillus
- ☐ Basidiobolomycosis
- ☐ Blastomyces dermatitidis
- ☐ Candida albicans
- ☐ Candida glabrata
- ☐ Candida guilliermondii
- ☐ Candida krusei
- ☐ Candida lusitaniae
- ☐ Candida parapsilosis
- ☐ Candida species
- ☐ Candida tropicalis
- ☐ Chromomycosis
- ☐ Coccidioides immitis
- ☐ Cryptococcus neoformans
- ☐ Cunninghamella
- ☐ Dermatophytes
- ☐ Fusarium
- ☐ Histoplasma capsulatum
- ☐ Mucor
- ☐ Mycetoma
- ☐ Pneumocystis carinii
- ☐ Pneumocystis jirovecii
- ☐ Pseudallescheria boydii
- ☐ Rhizomucor
- ☐ Rhizopus
- ☐ Saksanea
- ☐ Sporothrix schenckii
- ☐ Zygomycetes

4.51 INFECTION COMPLICATION 3:

<u>'Full' daily data collection</u>: Infectious complications diagnosed ***in the last 24 hours***

<u>'Basic' daily data collection</u>: Infectious complications diagnosed ***since the last EOT Daily form***

- *If this is the '**Four days after ICU admission'** timepoint, please answer with reference to the last 24 hours*

- ☐ Yes
- ☐ No

4.52 INFECTION COMPLICATION 3 DATE OF DIAGNOSIS:

__ __ / __ __ / __ __ __ __ (DD/MM/YYYY)

4.53 SOURCE OF INFECTIOUS COMPLICATION 3:

- ☐ Lungs
- ☐ Gastro-intestinal
- ☐ Genito-urinary
- ☐ Skin and soft tissue
- ☐ Central nervous system
- ☐ Osteoarticular and bone
- ☐ Cardiac
- ☐ Bloodstream
- ☐ Not known

4.54 CAUSATIVE PATHOGEN 3:

- ☐ Acinetobacter baumannii
- ☐ Actinomyces
- ☐ Aeromonas
- ☐ Bacillus anthracis
- ☐ Bacillus species
- ☐ Bacteroides fragilis
- ☐ Bacteroides species
- ☐ Bartonella species
- ☐ Bordetella species
- ☐ Borrelia burgdorferi
- ☐ Borrelia species
- ☐ Brucella Species
- ☐ Burkholderia cepacia
- ☐ Burkholderia mallei
- ☐ Burkholderia pseudomallei
- ☐ Campylobacter and related species
- ☐ Campylobacter jejuni
- ☐ Capnocytophaga canimorsus
- ☐ Chlamydia trachomatis
- ☐ Chlamydophila pneumoniae
- ☐ Chlamydophila psittaci
- ☐ Citrobacter species
- ☐ Clostridium botulinum
- ☐ Clostridium difficile
- ☐ Clostridium species
- ☐ Clostridium tetani (Tetanus)
- ☐ Corynebacterium diphtheriae
- ☐ Coxiella burnetii
- ☐ Ehrlichia species
- ☐ Eikenella corrodens
- ☐ Enterobacter species
- ☐ Enterococcus
- ☐ Erysipelothrix rhusiopathiae
- ☐ Escherichia coli
- ☐ Francisella tularensis
- ☐ Haemophilus ducreyi (Chancroid)
- ☐ Haemophilus influenzae
- ☐ Helicobacter cinaedi and related species
- ☐ Helicobacter pylori
- ☐ Klebsiella granulomatis (Antibiotic Guide)
- ☐ Klebsiella species
- ☐ ESBL Klebsiella pneumoniae
- ☐ Lactobacillus
- ☐ Legionella pneumophila
- ☐ Legionella species
- ☐ Leptospira interrogans
- ☐ Listeria monocytogenes
- ☐ Lymphogranuloma venereum (LGV)
- ☐ Methicillin Resistant Staphylococcus aureus
- ☐ Moraxella catarrhalis
- ☐ Morganella
- ☐ Mycobacterium abscessus
- ☐ Mycobacterium avium-complex (MAC, MAI, non-HIV)
- ☐ Mycobacterium chelonae
- ☐ Mycobacterium fortuitum
- ☐ Mycobacterium gordonae
- ☐ Mycobacterium kansasii
- ☐ Mycobacterium leprae
- ☐ Mycobacterium marinum
- ☐ Mycobacterium scrofulaceum
- ☐ Mycobacterium tuberculosis
- ☐ Mycobacterium ulcerans
- ☐ Mycobacterium xenopi
- ☐ Mycoplasma pneumoniae (Antibiotic Guide)
- ☐ Neisseria gonorrhoeae
- ☐ Neisseria meningitidis
- ☐ Nocardia
- ☐ Other atypical mycobacteria
- ☐ Pasteurella multocida
- ☐ Peptostreptococcus/Peptococcus
- ☐ Plesiomonas
- ☐ Propionibacterium species
- ☐ Proteus species
- ☐ Providencia
- ☐ Pseudomonas aeruginosa

- ☐ Rhodococcus equi
- ☐ Rickettsia rickettsii
- ☐ Rickettsia species
- ☐ Salmonella species
- ☐ Serratia species
- ☐ Shigella dysenteriae
- ☐ Shigella species
- ☐ Staphylococci, coagulase negative
- ☐ Staphylococcus aureus
- ☐ Stenotrophomonas maltophilia
- ☐ Streptobacillus moniliformis
- ☐ Streptococcus pneumoniae
- ☐ Streptococcus pyogenes (Group A)
- ☐ Streptococcus species
- ☐ Treponema pallidum (syphilis)
- ☐ Tropheryma whipplei
- ☐ Vancomycin Resistant Enterococcus species
- ☐ Vancomycin Resistant Staphylococcus aureus
- ☐ Vibrio cholerae
- ☐ Vibrio species (noncholera)
- ☐ Yersinia pestis
- ☐ Yersinia species (non-plague)
- ☐ Absidia
- ☐ Aspergillus
- ☐ Basidiobolomycosis
- ☐ Blastomyces dermatitidis
- ☐ Candida albicans
- ☐ Candida glabrata
- ☐ Candida guilliermondii
- ☐ Candida krusei
- ☐ Candida lusitaniae
- ☐ Candida parapsilosis
- ☐ Candida species
- ☐ Candida tropicalis
- ☐ Chromomycosis
- ☐ Coccidioides immitis
- ☐ Cryptococcus neoformans
- ☐ Cunninghamella
- ☐ Dermatophytes
- ☐ Fusarium
- ☐ Histoplasma capsulatum
- ☐ Mucor
- ☐ Mycetoma
- ☐ Pneumocystis carinii
- ☐ Pneumocystis jirovecii
- ☐ Pseudallescheria boydii
- ☐ Rhizomucor
- ☐ Rhizopus
- ☐ Saksanea
- ☐ Sporothrix schenckii
- ☐ Zygomycetes

4.55 HAEMORRHAGIC COMPLICATION 1:

<u>'Full' daily data collection:</u> Haemorrhagic complications diagnosed ***in the last 24 hours***

<u>'Basic' daily data collection</u>: Haemorrhagic complications diagnosed ***since the last EOT Daily form***

- *If this is the '**Four days after ICU admission'** timepoint, please answer with reference to the last 24 hours*

☐ Yes
☐ No

4.56 SOURCE OF HAEMORRHAGIC COMPLICATION 1:

- ☐ Lungs
- ☐ Gastro-intestinal
- ☐ Genito-urinary
- ☐ Skin and soft tissue
- ☐ Central nervous system
- ☐ Osteoarticular and bone
- ☐ Cardiac
- ☐ Bloodstream
- ☐ Not known

4.57 HAEMORRHAGIC COMPLICATION 2:

<u>'Full' daily data collection</u>: Haemorrhagic complications diagnosed ***in the last 24 hours***

<u>'Basic' daily data collection</u>: Haemorrhagic complications diagnosed ***since the last EOT Daily form***

- *If this is the **'Four days after ICU admission'** timepoint, please answer with reference to the last 24 hours*

☐ Yes
☐ No

4.58 SOURCE OF HAEMORRHAGIC COMPLICATION 2:

- ☐ Lungs
- ☐ Gastro-intestinal
- ☐ Genito-urinary
- ☐ Skin and soft tissue
- ☐ Central nervous system
- ☐ Osteoarticular and bone
- ☐ Cardiac
- ☐ Bloodstream
- ☐ Not known

4.59 OTHER NON-HAEMORRHAGIC COMPLICATION:

'Full' daily data collection: Haemorrhagic complications diagnosed ***in the last 24 hours***

'Basic' daily data collection: Haemorrhagic complications diagnosed ***since the last EOT Daily form***

- *If this is the 'Four days after ICU admission' timepoint, please answer with reference to the last 24 hours*

____ ____(TEXT)

4.60 Troponin in the last 24 hours:

- ☐ Troponin T: __________ (☐ ng/mL ☐ ng/L)
- ☐ Troponin I: __________ (☐ ng/mL ☐ ng/L)

  If this data has already been entered in the 'Daily Case Report Form – Laboratory Results' section of the ISARIC CRF, please DO NOT re-enter the data here. Please leave '4.59 Troponin I' blank.
- ☐ High sensitivity troponin T: __________ (☐ ng/mL ☐ ng/L)
- ☐ High sensitivity troponin I: __________ (☐ ng/mL ☐ ng/L)
- ☐ Not available

4.61 Cardiac BNP in the last 24 hours: __________ (picograms/mL) ONLY NUMBERS BETWEEN 0-1000

- ☐ Not available

CORE CASE RECORD FORM (EOT Final)

5 OUTCOMES

5.1 DATE OF ECMO DISCONTINUATION: _____ / _____ / _____

5.2 DATE OF INVASIVE MECHANICAL VENTILATION DISCONTINUATION: _____ / _____ / _____

5.3 DATE OF ICU DISCHARGE: _____ / _____ / _____

5.4 DATE OF HOSPITAL DISCHARGE: _____ / _____ / _____

5.5 DATE OF DEATH: _____ / _____ / _____ ☐ Not applicable

5.6 SITE OF DEATH

☐ ICU

☐ HOSPITAL

☐ OUTSIDE HOSPITAL

☐ Not applicable

5.7 MAIN CAUSE OF ICU DEATH

☐ Respiratory Failure

☐ Cardiac Failure

☐ Liver Failure

☐ Cerebrovascular accident

☐ Septic shock

☐ Haemorrhagic shock

☐ Other

☐ Not applicable

5.8 ALIVE AT 28 DAYS POST ICU ADMISSION?

☐ Yes

☐ No

5.9 FINAL ASSESSMENT NOTES

____

____

5.10 At any time post-ICU admission and until ICU discharge, did the patient present new cutaneous manifestations?

☐ Yes

☐ No

☐ Not available

If yes to 5.10, type of cutaneous manifestations (please select up to three (3) options)

☐ Bullae

- ◻ Macules
- ◻ Nodules
- ◻ Papules
- ◻ Plaques
- ◻ Purpura
- ◻ Pustules
- ◻ Rash
- ◻ Scale
- ◻ Urticaria
- ◻ Vesicles
- ◻ Other: __________

If yes to 5.10, specify the involved regions (please select up to three (3) options):

- ◻ Face
- ◻ Truck
- ◻ Upper limbs
- ◻ Hands
- ◻ Lower limbs
- ◻ Feet

5.11 At any time post ICU admission and until ICU discharge, did the patient have a stroke?

- ◻ Yes
- ◻ No
- ◻ Not available

If yes to 5.11, type of stroke (please select up to two (2) options)

- ◻ Ischemic stroke
- ◻ Intraparenchymal haemorrhage
- ◻ Subarachnoid haemorrhage
- ◻ Hypoxic ischemic brain injury/anoxic brain injury
- ◻ Cerebral venous sinus thrombosis
- ◻ Other
- ◻ Unknown

If yes to 5.11, side of stroke (please select only one)

- ◻ Right side
- ◻ Left side
- ◻ Multifocal
- ◻ Unknown
