# Supplementary material for: Impact of renin–angiotensin–aldosterone system inhibition on mortality in critically ill COVID-19 patients with pre-existing hypertension: a prospective cohort study
Source: BMC Cardiovasc Disord. 2022 Mar 23;22:123. doi: 10.1186/s12872-022-02565-1 (PMC8942148; doi:10.1186/s12872-022-02565-1)
Supplement: Supplementary file 2 — Additional file 2: Figure S1. Multistate model structure.Figure S2. Flow diagram.Figure S3. Stratified baseline survival functions by corticosteroid use from Cox proportional hazards models.Table S1. Complications at any time during hospitalization.Table S2. Expected length of stay estimated from the multistate model.Table S3. Multi-state Cox proportional hazards model. Table S4. Propensity score adjusted estimates for the hazards of death and discharge.Table S5. Sensitivity analysis, Multi-state Cox proportional hazards model.Table S6. Sensitivity analysis, Propensity score adjusted estimates for the hazards of death and discharge. [file 12872_2022_2565_MOESM2_ESM.docx]

SUPPLEMENTAL MATERIAL

**(Additional File 2)**

**Figure S1:** Multistate model structure

**Figure S2:** Flow diagram

**Figure S3:** Stratified baseline survival functions by corticosteroid use from Cox proportional hazards models

**Table S1:** Complications at any time during hospitalization

**Table S2:** Expected length of stay estimated from the multistate model.

**Table S3:** Multi-state Cox proportional hazards model

**Table S4:** Propensity score adjusted estimates for the hazards of death and discharge

**Table S5:** Sensitivity analysis, Multi-state Cox proportional hazards model

**Table S6:** Sensitivity analysis, Propensity score adjusted estimates for the hazards of death and discharge

**Supplemental Figures**

**Figure S1.** Multistate model structure. ICU: intensive care unit


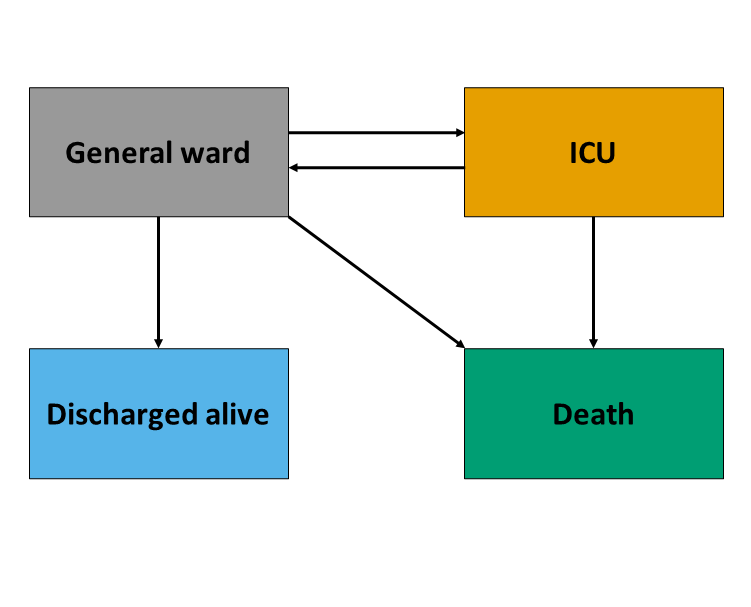


**Figure S2.** Flow diagram. ACEi: angiotensin-converting enzyme inhibitor, ARB: angiotensin II receptor blocker, COVID-19: Coronavirus disease-19


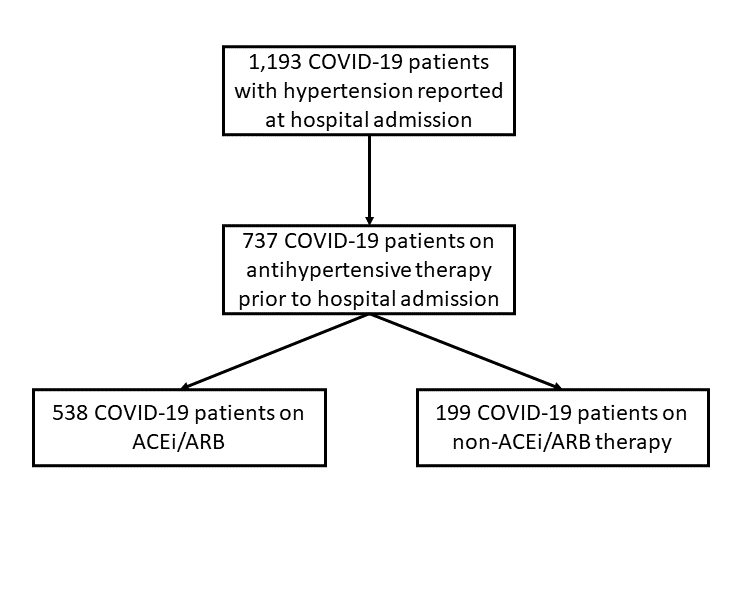


**Figure S3:** Stratified baseline survival functions by corticosteroid use (Blue = Yes, Red = No) from Cox proportional hazards models. Results for both the full Cox proportional hazards model (solid line) and propensity score adjustment (dashed line). A: Died in hospital, B: Discharged alive from hospital


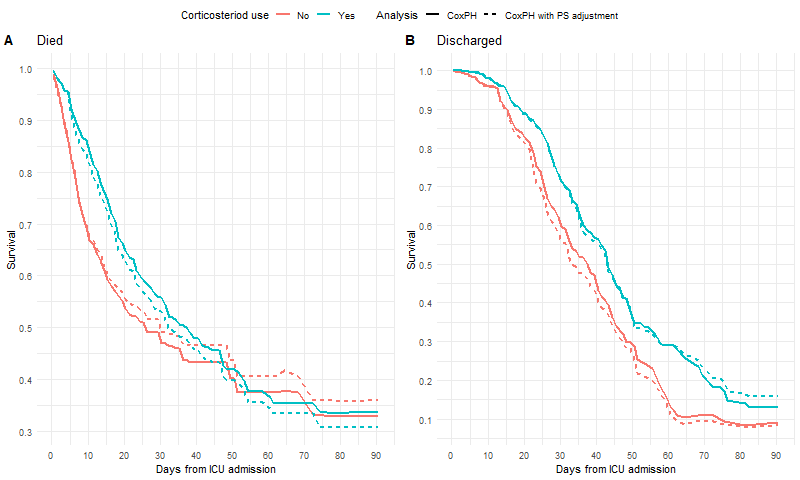


**Supplemental Tables**

**Table S1.** Complications at any time during hospitalization

| **Complication** | **ACEi/ARB**  **n (%)** | **Available number** | **non-ACEi/ARB**  **n (%)** | **Available number** | **p value** |
| --- | --- | --- | --- | --- | --- |
| Stroke | 25 (5) | 461 | 8 (5) | 172 | 0.85 |
| Heart failure | 37 (8) | 454 | 18 (11) | 171 | 0.43 |
| Cardiac arrhythmia | 152 (33) | 459 | 44 (25) | 174 | 0.068 |
| Cardiac ischemia | 21 (5) | 457 | 11 (7) | 169 | 0.31 |
| Cardiac arrest | 78 (17) | 463 | 40 (23) | 172 | 0.065 |

ACEi: angiotensin-converting enzyme inhibitor, ARB: angiotensin II receptor blocker

**Table S2.** Expected length of ICU and hospital stay and mortality risk estimated from the multistate model

|  | Expected Length of stay:  Mean days (95% CI) | | Mortality risk following ICU admission: Mean % (SE) | | |
| --- | --- | --- | --- | --- | --- |
|  | ICU | General ward | 30 day | 60 day | 90 day |
| ACEi/ARB | 21.2 (19.7 to 22.8) | 6.7 (5.9 to 7.6) | 43.5 (2.2) | 50.3 (2.2) | 51.0 (2.2) |
| non-ACEi/ARB | 16.2 (14.1 to 18.6) | 6.4 (5.1 to 7.9) | 51.4 (3.7) | 59.0 (3.6) | 59.0 (3.6) |

This does not include adjustment for baseline covariates. ACEi: angiotensin-converting enzyme inhibitor, ARB: angiotensin II receptor blocker, CI: confidence intervals, ICU: intensive care unit, SE: standard error

**Table S3.** Multi-state Cox proportional hazards model

| Outcome from ICU admission | Characteristic | HR | SE | 95% CI | p value |
| --- | --- | --- | --- | --- | --- |
| Death | Group = ACEi/ARB | 0.74 | 0.12 | 0.58 to 0.94 | 0.015 |
|  | Age (+ 10 years) | 1.244 | 0.053 | (1.121 to 1.38) | <0.001 |
|  | BMI (+ 5kg/m2) | 0.963 | 0.038 | (0.894 to 1.038) | 0.322 |
|  | Sex = Male | 0.886 | 0.118 | (0.703 to 1.118) | 0.309 |
|  | Diabetes | 1.139 | 0.109 | (0.919 to 1.412) | 0.233 |
|  | Smoking | 1.074 | 0.138 | (0.816 to 1.413) | 0.608 |
|  | Chronic cardiac disease | 1.078 | 0.123 | (0.846 to 1.373) | 0.544 |
|  | Chronic kidney disease | 1.307 | 0.137 | (0.998 to 1.711) | 0.051 |
|  | Week of ICU admission (+ 1 week) | 1.142 | 0.037 | (1.062 to 1.227) | <0.001 |
|  | Region = Africa | 1.435 | 0.515 | (0.52 to 3.958) | 0.484 |
|  | Region = Asia | 0.829 | 0.274 | (0.484 to 1.42) | 0.495 |
|  | Region = Australia and New Zealand | 0.292 | 0.740 | (0.068 to 1.251) | 0.097 |
|  | Region = Latin America and the Caribbean | 1.132 | 0.227 | (0.726 to 1.767) | 0.584 |
|  | Region = Northern America | 0.961 | 0.154 | (0.711 to 1.299) | 0.794 |
|  | Ethnicity = Black | 0.999 | 0.191 | (0.687 to 1.454) | 0.997 |
|  | Ethnicity = Latin American | 1.046 | 0.206 | (0.698 to 1.568) | 0.826 |
|  | Ethnicity = South Asian | 3.941 | 0.302 | (2.177 to 4) | <0.001 |
|  | Ethnicity = Other | 1.273 | 0.238 | (0.797 to 2.034) | 0.312 |
| Discharged alive | Group = ACEi/ARB | 0.86 | 0.15 | 0.64 to 1.5 | 0.307 |
|  | Age (+ 10 years) | 0.86 | 0.06 | 0.76 to 0.98 | 0.019 |
|  | BMI (+ 5kg/m2) | 0.98 | 0.04 | 0.92 to 1.07 | 0.769 |
|  | Sex = Male | 0.72 | 0.14 | 0.54 to 0.94 | 0.017 |
|  | Diabetes | 1.05 | 0.13 | 0.82 to 1.34 | 0.417 |
|  | Smoking | 1.12 | 0.14 | 0.85 to 1.47 | 0.417 |
|  | Chronic cardiac disease | 0.90 | 0.16 | 0.66 to 1.23 | 0.510 |
|  | Chronic kidney disease | 1.14 | 0.18 | 0.79 to 1.62 | 0.485 |
|  | Week of ICU admission (+ 1 week) | 0.99 | 0.05 | 0.90 to 1.09 | 0.85 |
|  | Region = Africa | 2.22 | 0.70 | 0.56 to 8.90 | 0.126 |
|  | Region = Asia | 1.06 | 0.40 | 0.48 to 2.34 | 0.878 |
|  | Region = Australia and New Zealand | 2.32 | 0.55 | 0.79 to 6.80 | 0.13 |
|  | Region = Latin America and the Caribbean | 2.08 | 0.24 | 1.29 to 3.35 | 0.003 |
|  | Region = Northern America | 1.94 | 0.17 | 1.38 to 2.73 | <0.001 |
|  | Ethnicity = Black | 0.64 | 0.20 | 0.43 to 0.96 | 0.032 |
|  | Ethnicity = Latin American | 0.73 | 0.21 | 0.48 to 1.11 | 0.137 |
|  | Ethnicity = South Asian | 0.22 | 0.77 | 0.05 to 0.97 | 0.045 |
|  | Ethnicity = Other | 0.63 | 0.32 | 0.33 to 1.20 | 0.159 |

This accounts for competing risks of in-hospital death and hospital discharge up to 90 days from ICU admission. Effect to patient group (ACEi/ARB, non-ACEi/ARB) assumed fixed over time; random effect per recruiting site. Model results are pooled from 10 rounds of multiple imputation using chained equations (MICE). ACEi: angiotensin-converting enzyme inhibitor, ARB: angiotensin II receptor blocker, CI: confidence intervals, ICU: intensive care unit, HR: hazard ratio, SE: standard error

**Table S4.** Propensity score adjusted estimates for the hazards of death and discharge

| Outcome from ICU admission | Group | HR | SE | 95% CI | p value |
| --- | --- | --- | --- | --- | --- |
| Discharged alive | ACEi/ARB | 0.89 | 0.15 | (0.67 to 1.19) | 0.428 |
| Death | ACEi/ARB | 0.73 | 0.12 | (0.58 to 0.93) | 0.009 |

**Table S5.** Sensitivity analysis, Multi-state Cox proportional hazards model

| Outcome from ICU admission | Characteristic | HR | SE | 95% CI | p value |
| --- | --- | --- | --- | --- | --- |
| Death | Group = ACEi | 0.69 | 0.14 | (0.52 to 0.91) | 0.01 |
|  | Group = ARB | 0.77 | 0.14 | (0.58 to 1.01) | 0.062 |
|  | Age + 10 years | 1.26 | 0.05 | (1.13 to 1.4) | <0.001 |
|  | BMI + 5kg/m2 | 0.97 | 0.04 | (0.9 to 1.05) | 0.5 |
|  | Sex = Male | 0.89 | 0.12 | (0.7 to 1.13) | 0.328 |
|  | Diabetes | 1.11 | 0.11 | (0.89 to 1.39) | 0.346 |
|  | Smoking | 1.03 | 0.13 | (0.79 to 1.34) | 0.834 |
|  | Chronic cardiac disease | 1.05 | 0.13 | (0.82 to 1.35) | 0.685 |
|  | Chronic kidney disease | 1.31 | 0.14 | (0.99 to 1.73) | 0.057 |
|  | Week of ICU admission + 1 week | 1.14 | 0.04 | (1.05 to 1.23) | <0.001 |
|  | Region = Africa | 1.30 | 0.50 | (0.49 to 3.48) | 0.595 |
|  | Region = Asia | 0.74 | 0.28 | (0.43 to 1.29) | 0.286 |
|  | Region = Australia and New Zealand | 0.17 | 1.03 | (0.02 to 1.26) | 0.083 |
|  | Region = Latin America and the Caribbean | 1.18 | 0.24 | (0.73 to 1.91) | 0.492 |
|  | Region = Northern America | 0.94 | 0.16 | (0.69 to 1.29) | 0.705 |
|  | Ethnicity = Black | 1.01 | 0.20 | (0.69 to 1.5) | 0.947 |
|  | Ethnicity = Latin American | 1.05 | 0.22 | (0.69 to 1.61) | 0.811 |
|  | Ethnicity = South Asian | 4.24 | 0.31 | (2.3 to 5) | <0.001 |
|  | Ethnicity = Other | 1.38 | 0.24 | (0.86 to 2.22) | 0.176 |
| Discharged alive | Group = ACEi | 0.78 | 0.17 | (0.56 to 1.1) | 0.156 |
|  | Group = ARB | 0.94 | 0.17 | (0.67 to 1.31) | 0.695 |
|  | Age + 10 years | 0.87 | 0.06 | (0.77 to 0.99) | 0.029 |
|  | BMI + 5kg/m2 | 0.98 | 0.04 | (0.91 to 1.06) | 0.612 |
|  | Sex = Male | 0.75 | 0.15 | (0.57 to 1) | 0.05 |
|  | Diabetes | 1.04 | 0.13 | (0.81 to 1.35) | 0.745 |
|  | Smoking | 1.07 | 0.15 | (0.8 to 1.43) | 0.664 |
|  | Chronic cardiac disease | 0.86 | 0.16 | (0.62 to 1.19) | 0.372 |
|  | Chronic kidney disease | 1.15 | 0.19 | (0.79 to 1.66) | 0.464 |
|  | Week of ICU admission + 1 week | 1.00 | 0.05 | (0.91 to 1.11) | 0.939 |
|  | Region = Africa | 2.35 | 0.68 | (0.62 to 5) | 0.21 |
|  | Region = Asia | 1.23 | 0.41 | (0.55 to 2.75) | 0.617 |
|  | Region = Australia and New Zealand | 2.57 | 0.63 | (0.75 to 5) | 0.133 |
|  | Region = Latin America and the Caribbean | 2.34 | 0.27 | (1.38 to 3.97) | 0.002 |
|  | Region = Northern America | 2.13 | 0.18 | (1.49 to 3.03) | <0.001 |
|  | Ethnicity = Black | 0.64 | 0.20 | (0.43 to 0.96) | 0.029 |
|  | Ethnicity = Latin American | 0.69 | 0.22 | (0.45 to 1.06) | 0.09 |
|  | Ethnicity = South Asian | 0.20 | 0.79 | (0.04 to 0.94) | 0.041 |
|  | Ethnicity = Other | 0.59 | 0.34 | (0.3 to 1.15) | 0.122 |

This accounts for competing risks of in-hospital death and hospital discharge up to 90 days from ICU admission. Effect to patient group ACEi/ARB, non-ACEi/ARB assumed fixed over time; random effect per recruiting site. Model results are pooled from 10 rounds of multiple imputation using chained equations MICE. ACEi: angiotensin-converting enzyme inhibitor, ARB: angiotensin II receptor blocker, CI: confidence intervals, ICU: intensive care unit, HR: hazard ratio, SE: standard error

**Table S6.** Sensitivity analysis: Propensity score adjusted estimated for the hazards of death and discharge between non-ACEi/ARB, ACEi and ARB groups

| Outcome from ICU admission | Group | HR | SE | 95% CI | p value |
| --- | --- | --- | --- | --- | --- |
| Death | ACEi | 0.74 | 0.14 | (0.56 to 0.96) | 0.026 |
|  | ARB | 0.75 | 0.14 | (0.57 to 0.97) | 0.031 |
| Discharged alive | ACEi | 0.87 | 0.17 | (0.63 to 1.21) | 0.409 |
|  | ARB | 0.98 | 0.16 | (0.71 to 1.35) | 0.908 |

Cause-specific Cox proportional hazards model weighted by inverse probabilities 1/propensity score of belonging to the ACEi/ARB group, accounting for competing risks. Subject probabilities of belonging to the ACEi/ARB group were estimated as a function of age, sex, BMI, diabetes, smoking, chronic cardiac disease, chronic kidney disease and calendar time. Model results were pooled from 10 rounds of multiple imputation using chained equations MICE. ACEi: angiotensin-converting enzyme inhibitor, ARB: angiotensin II receptor blocker, CI: confidence intervals, ICU: intensive care unit, HR: hazard ratio, SE: standard error
